# ^1^H NMR Spectroscopy Profiling of Metabolic Reprogramming of Chinese Hamster Ovary Cells upon a Temperature Shift during Culture

**DOI:** 10.1371/journal.pone.0077195

**Published:** 2013-10-10

**Authors:** Jane L. Wagstaff, Rosalyn J. Masterton, Jane F. Povey, C. Mark Smales, Mark J. Howard

**Affiliations:** Centre for Molecular Processing and School of Biosciences, University of Kent, Canterbury, Kent, United Kingdom; Spanish National Cancer Center, Spain

## Abstract

We report an NMR based approach to determine the metabolic reprogramming of Chinese hamster ovary cells upon a temperature shift during culture by investigating the extracellular cell culture media and intracellular metabolome of CHOK1 and CHO-S cells during culture and in response to cold-shock and subsequent recovery from hypothermic culturing. A total of 24 components were identified for CHOK1 and 29 components identified for CHO-S cell systems including the observation that CHO-S media contains 5.6 times the level of glucose of CHOK1 media at time zero. We confirm that an NMR metabolic approach provides quantitative analysis of components such as glucose and alanine with both cell lines responding in a similar manner and comparable to previously reported data. However, analysis of lactate confirms a differentiation between CHOK1 and CHO-S and that reprogramming of metabolism in response to temperature was cell line specific. The significance of our results is presented using principal component analysis (PCA) that confirms changes in metabolite profile in response to temperature and recovery. Ultimately, our approach demonstrates the capability of NMR providing real-time analysis to detect reprogramming of metabolism upon cellular perception of cold-shock/sub-physiological temperatures. This has the potential to allow manipulation of metabolites in culture supernatant to improve growth or productivity.

## Introduction

Mammalian cell lines are routinely utilised in the bioprocessing industry to manufacture high-value biotherapeutic proteins and are most often favoured when the target protein has specific post-translational processing requirements that cannot be undertaken by other expression systems [1]. The current mammalian cell line of choice and industrial gold-standard is the Chinese hamster ovary (CHO) cell line which has been used to manufacture a range of biotherapeutic proteins now in the clinic [1]. The development of mammalian cell lines for high level expression of recombinant proteins has progressed such that cell line construction projects now routinely produce cell lines that are capable of delivering gram per litre titres of product in fed-batch cultures [2]. The enhancements to yields has largely been the result of expression vectors manipulation, improved host cell lines, development of medium and feeding regimes, and advanced screening techniques that have dramatically improved the viable cell concentrations obtained in the bioreactor and reduced the time taken to produce stable production cell lines [1]. Another approach used during the manufacturing of recombinant therapeutic proteins from CHO cells to enhance titre is to use a temperature shift strategy whereby the cells are initially cultured *in vitro* at 37°C in order to try and duplicate those conditions found *in vivo* and to allow raid accumulation of biomass after which time the temperature is reduced to 30-34°C [3-8]. The reduction in temperature has been shown, in some cases, to result in the improvement of both cell specific and volumetric productivity, protein folding and product quality. At reduced temperatures the metabolism and energy requirements of the cell also changes [9] although how this reflects changes in net intracellular and extracellular metabolite profiles and the biology that underpins these changes has not been extensively studied.

Despite the significant enhancements in cell culture process developments, specifically the design of culture media and feeding protocols for improved recombinant protein production, such advancements have largely been derived from ‘design-of-experiment’ phenotype observations (e.g. growth and productivity) and subsequent manipulation of media components and feeding without necessarily considering the metabolic state or need of the cells in culture [10,11]. A small number of studies have now begun to use global metabolic profiling to identify changes in the flux of metabolites both intra- and extra-cellularly to highlight changes in the flux of key metabolites, identify potentially limiting metabolites or the buildup of toxic metabolites to inform the development of new media and feeding regimes that reflect the need of the cell. For example, Sellick and colleagues used a GC-MS based approach to profile changes in the intra- and extra-cellular concentrations of key metabolites in a CHO cell line expressing a model monoclonal antibody, subsequently identifying limiting nutrients. Using this knowledge they designed a new feeding approach to replace these nutrients that resulted in a doubling of the antibody titre and an increase in biomass of approximately 35% [12]. Selvarasu et al used a combined *in silico* modeling and metabolic profiling approach to characterize fed-batch CHO cell cultures, identifying key metabolites associated with cell growth limitation related to energy, glutathione and glycerophospholipid synthesis alongside amino acid content in CHO cells that differed from other cell types [13]. Chong and colleagues also used a metabolomics-based approach to time profile extracellular metabolites in duplicate fed-batch bioreactor cultures of recombinant CHO cells producing a monoclonal IgG antibody using HPLC coupled with an LTQ-Orbitrap mass spectrometer. Using this approach they identified two groups of metabolites; those accumulated when the culture entered stationary phase and those that decreased in abundance as the culture progressed [14]. Isotopic labeling with [1,2-(13)C]glucose and [U-(13)C]glutamine coupled with GS-MS has also been used to determine metabolic fluxes in CHO cells [15] whilst Dietmair et al also recently reported using GC-MS to profile CHO cell metabolites in cells with different growth characteristics [16]. 

Bradley et al were the first to use NMR spectroscopy to quantitate metabolites in CHO cell culture medium with the ultimate aim of using such data for the rational optimization of CHO based media to enhance therapeutic protein production. These authors reported on the development of a methodology they termed ‘Fermentanomics’ to generate appropriate spectra whereby 50 components were identified [17]. A subsequent study by Aranibar et al used NMR to profile and quantify 30 key metabolites in a CHO cell line engineered to express a model fusion protein demonstrating that the metabolite measurements correlated with currently accepted endpoint based measures, validating the use of NMR for such an approach [18]. NMR has the advantage over other methods such as GC-MS in that labeling/derivatization of the metabolites is not required which reduces the time taken to process samples and in principle, measurements can be made in real time. Despite these studies, the application of NMR metabolite screening to improving our understanding of CHO cell metabolism has not been widely applied and no global metabolite screening approach has been used to investigate the flux of metabolites upon reduced temperature cultivation of CHO cells. In our studies we have investigated the metabolic profiles in an adherent CHOK1 cell line and a suspension CHO cell line to determine if differences between these growth modes and then followed the metabolic reprogramming and flux in these cell lines upon reduced temperature cultivation. 

## Materials and Methods

### Reagents

All chemicals were purchased from Sigma-Aldrich (Sigma-Aldrich Ltd., U.K.) and were of analytical reagent grade or superior unless otherwise stated. 

### Cell culture techniques

#### Cell lines

Adherent Chinese hamster ovary K1 (CHOK1) cells were purchased from ECACC and maintained in Dulbecco’s Modified Eagle Medium (DMEM) nutrient mix F12 (Gibco, Invitrogen, U.K.) supplemented with 6 mM L-glutamine, 500 μM glutamic acid, 500 μM asparagine, 30 μM adenosine, 30 μM guanosine, 30 μM cytidine, 30 μM uridine, 10 μM thymidine, 1% (v/v) non-essential amino acids (100 x) (Invitrogen, U.K.), and 10% (v/v) dialyzed foetal bovine serum (Lonza, U.K.). The suspension-adapted CHO-S (Invitrogen, UK) cell line was maintained in CD-CHO medium (Invitrogen, UK) supplemented with 8 mM glutamine. Tables of media components and concentrations are listed in Tables S7-S8 in File S1.

#### Timecourse growth curves and sample collection of adherent CHO cells (CHOK1) at various culture temperatures

Twenty-four T25 flat flasks were seeded with 2x10^5^ CHOK1 cells/ml in 10 ml volume and incubated at 37°C, 5% CO_2_. The cells were cultured at 37°C until they reached mid-exponential phase of growth which occurred at 72 h. Seven of the flasks were then shifted to 27°C and seven to 10°C, 5% CO_2_ for 24 h. The remaining flasks stayed at 37°C. After incubation for 24 hours three of the flasks at 27°C and three at 10°C were returned to the 37°C incubator. This approach ensured that all conditions were available for scrutiny in triplicate. At the culture time points 0, 24, 48, 72, 96, 120, 144, 216 h the supernatant was removed from the cells from a flask at each of the temperatures investigated and stored at -80°C in 1 ml aliquots (we harvested all the supernatant from a flask at each time point for the analysis so each time point represents that from a unique flask all seeded and originating from the same initial culture). Culture supernatant was collected by pouring off the media and then centrifugation in a bench top centrifuge to remove cells and cell debris from the media. To collect cells, these were scraped from the plate once the media was removed, an aliquot was cell counted using the Vi-Cell and the remaining cells were quenched. In all cases, time point zero refers to when the cells were seeded initially seeded.

#### Timecourse growth curves and sample collection of suspension CHO cells (CHO-S) at various culture temperatures

Five shake flasks were initially seeded at 0.05 x 10^6^ cells/ml in 100 ml of media. The cells were cultured at 37°C until they reached mid-exponential phase of growth which occurred at 72 h. At this time point one flask was left at 37°C as a control, two were shifted to 27°C and two to 10°C. After a further 24 hours, the 37°C flask remained at this temperature, one of each of the flasks at 27°C and 10°C were left at the respective temperatures, whilst the second of the flasks at 27°C and 10°C were returned to 37°C for rewarming. Cell counts were taken using a Vi-cell instrument (Beckman Coulter, UK) that utilises the trypan blue dye exclusion method and the cell viabilities reported are those determined using this instrument. Supernatant was collected at time points 0 and 24 h and samples were quenched in addition to collecting cell counts and supernatants at 48, 72, 96, 120, 144, and 216 h. After seeding and every measurement the flasks were flushed with 5% CO_2_ before resealing the lid.

#### Whole cell quenching protocol

Whole cells were harvested and prepared for analysis as previously described [19]. Briefly, after completing cell counts, a volume of culture containing 1x10^7^ cells were harvested from the growing culture and metabolism quenched by the addition of a 5x volume of 60% methanol supplemented with 0.85% (W/V) ammonium bicarbonate at a minimum temperature of -40°C. Quenched cells were pelleted by centrifugation and the pellet rinsed before being resuspended in 0.5 mL of 100% methanol, flash frozen on liquid nitrogen and when thawed, pelleted by centrifugation. The supernatant was retained and the extraction process repeated. The pooled 1 mL of extracted metabolites were dried by speed vac and stored at -80°C until NMR sample preparation.

### NMR sample preparation and analysis

#### Preparation of cell culture media

Metabolites within the CHOK1 media were prepared for NMR analysis by methanol:chloroform extraction. This was necessary because residual protein from the serum added to the DMEM medium affected the baseline on the 1D proton NMR experiments and consequently accurate metabolite quantification (Figure S8 in File S1). 600 μL of media were mixed with 1.8 mL of a 2:1 ice cold methanol:chloroform solution and vortexed for 30 seconds. 600 μL of ice cold methanol was added, and the sample vortexed. The solution was centrifuged at 13000 rpm for 20 minutes and the aqueous layer collected and dried using a speed vac (drying time approximately 1 hour). Dried metabolites were stored at -80°C.

#### NMR sample preparation

For the analysis of CHO-S extracellular media, 600 μL of media was combined with 40 μL of D_2_O (Goss Scientific Ltd, UK), 10 μL of DSS (660 µM stock in D_2_O, making final concentration of 10 μM), and 10 μL of a 5% (w/v) sodium azide in D_2_O stock solution. For the analysis of the dried media metabolites and quenched cell extracts the dried material was resuspended in 640 μL of D_2_O (pH corrected to 7 using NaOD (Goss Scientific Ltd, UK)), and 10 μL of each DSS and sodium azide stock also added. All NMR samples were then transferred to a 5 mm Wilmad 535 NMR tube and stored briefly at 4°C before NMR analysis.

#### NMR data collection

All spectra were recorded at 37°C on a UnityINOVA 600 MHz NMR spectrometer (Varian Inc, Palo Alto, CA, USA) fitted with a 5 mm triple resonance HCN probe with z-shield gradients. All spectra were referenced to DSS at 0.00 ppm for ^1^H and to carrier at 53.611 ppm for ^13^C [20]. 1D ^1^H spectra with WATERGATE solvent suppression were recorded over 32768 points, 512 transients and a spectral width of 8004.8 Hz. An acquisition time of 2.047 s and a relaxation delay of 1.5 s. This provides an overall excitation recovery time of 3.547 s and produces a total experimental time of 31 min that enables efficient and fast data acquisition with minimal effect due to relaxation; ^1^H T_1_ values up to 1.5 s will experience an effective magnetization recovery greater than 90%. Data processing by ACD Labs SpecManager V9.0 involved zero-filling to 65536 points and multiplication of 1 Hz exponential line-broadening before Fourier transformation. For Metabolite identification purposes, ^1^H-^1^H TOCSY, g-DQFCOSY and ^1^H-^13^C HSQC experiments were completed for all 0 h and 48 h 37°C samples. ^1^H-^1^H TOCSY spectra with WATERGATE solvent suppression were collected with a width of 8000.0 Hz in both directions over 8192 points, 16 transients and 1024 complex points. An acquisition time of 0.512 s and a relaxation delay of 2 s gave an experiment time of 12 h. Each g-DQFCOSY experiment was run with WATERGATE solvent suppression and spectral widths of 8000.0 Hz in both directions. Spectra were collected over 8192 points, 1024 complex points and 16 transients. An acquisition time of 0.512 s and a relaxation delay of 2 s meant total experimental time for a g-DQFCOSY was 11 h 40 min. WATERGATE solvent suppressed ^1^H-^13^C HSQC spectra were run with spectral widths of 8000 Hz in the ^1^H dimension and 18001.8 Hz in the ^13^C dimension. Data were collected over 2048 points, 256 complex points and 128 transients; therefore with a relaxation delay of 2 s and an acquisition time of 0.128 s, the total experimental time was 19 h, 37 min. 

#### Metabolite identification by 2D NMR analysis

Metabolites present in NMR samples were identified by comparison of related chemical shifts with published NMR data of known metabolites, using the following online resources; the Madison Metabolomics Consortium Database (MMCD) [21] and the Spectral Database for Organic Compounds (SDBS) http://riodb01.ibase.aist.go.jp/sdbs/ (National Institute of Advanced Industrial Science and Technology, October-December 2010). Initial targets for metabolite identification were drawn from the known metabolite components of DMEM medium, and subsequent cross comparisons made to the CD-CHO medium. 

#### 1D ^1^H NMR data analysis

All ^1^H WATERGATE spectra were processed using ACD labs SpecManager V9.0 as described above then automatically phased and baseline corrected using built-in program functions. The binomial nature of the WATERGATE sequence was set to provide null points at ± 4800 Hz (± 8.00 ppm) with a narrow suppression profile of ± 450 Hz (± 0.75 ppm) about the water resonance placed at the NMR carrier frequency. Spectra were then referenced to 4,4-dimethyl-4-disilapentane-1-sulfonic acid (DSS) at 0.00ppm. Treating each cell lines temperature time-course as a group, regions containing no discernable peaks (9.5 to 12.3 ppm, and -3 to -0.5 ppm) were excluded from the integration by assignment to “dark regions”. Automatic intelligent bucket integration was then carried out using a target bucket width of 0.03 ppm splitting the spectra into approximately 250 separate slices. Automatic intelligent bucketing is a feature of the ACD labs SpecManager program that splits the 1D spectrum into sections or buckets. This is not done arbitrarily, but with reference to peaks within the spectra making some sections larger or smaller to fit the entire peak in one section, allowing easier quantification but integration of the peaks within each section. However, some bucket areas did require manual adjustment in order that resonances from the same metabolites were included in the same bucket. As the DSS peak at 0.00 ppm is composed of 9 chemically equivalent protons, the integrated area of this peak is equivalent to 90 μM of protons. The number of protons within each metabolite peak was then accounted for in the final μM concentration. Glucose in solution adopts two major cyclic structural forms, α and β, with 36% of the total α-glucose, therefore the concentration of total glucose was adjusted from the integration of the anomeric α-glucose peak which was adjusted to account for attenuation caused by the WATERGATE solvent suppression.

#### Curve fitting

Quantitative analysis was achieved by curve fitting metabolite concentration with time to a sigmoid function shown below using KaleidaGraph 3.6 (Synergy Software). Each curve was plotted as metabolite concentration (y) and time in hours (x). 

$$y=y_{o}+\frac{ax^{b}}{c^{b}+x^{b}}$$

Where *y*_*o*_ is the initial value of y, y_max_ is the maximum y value achievable from the expression and *a* = (y_max_ - y_o_). Therefore sigmoid curves have positive ‘*a*’ values when the curve rises and negative ‘*a*’ values when the curve falls. ‘*c*’ is defined as the value of ‘x’ at the half point between y_max_ and y_o_ in the expression and ‘*b*’ is known as the Hill coefficient and defines the slope of the curve. Standard errors were taken from the Levenberg-Marquardt fitting routine as the uncertainties.

### PCA Analysis

Standard PCA was undertaken on the quantified extracellular and intracellular metabolites (not including contaminants such as ethanol) using MATLAB software version 11 (The Math Works, MA, USA) to determine any clustering of the metabolite profile or fingerprints of samples. Eight sets of PCA analysis were completed, for the extracellular and intracellular metabolites of CHO-S and CHOK1 cultures with data grouped either by culture temperature condition or by time point to allow easier identification of each sample in the PCA.

## Results

**Figure 1:**
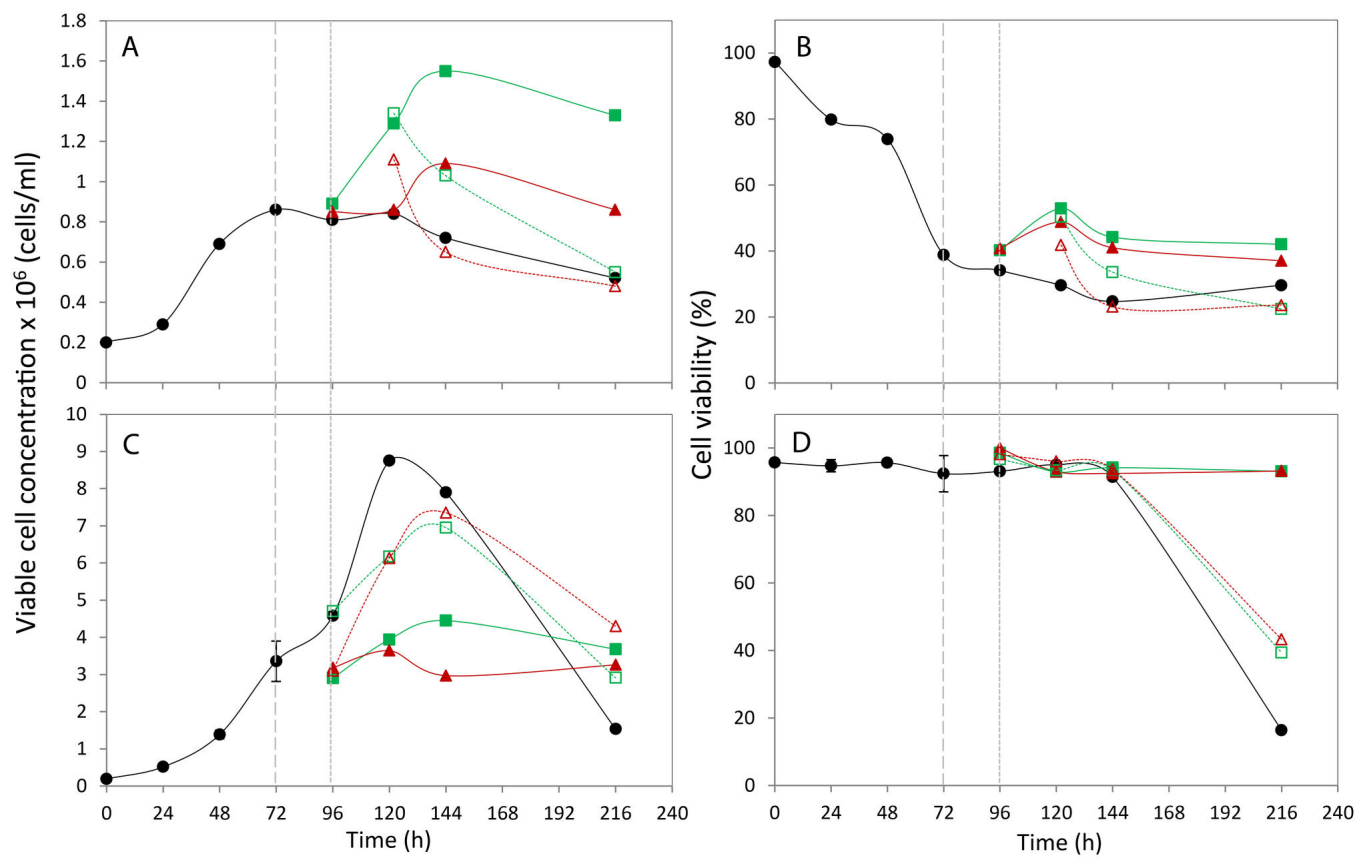
Growth profiles and cell viability of CHOK1 (A + B) and CHOS (C + D). Cells were grown at 37°C (•) or shifted to 27°C (■) or 10°C (▲). The timepoint of culture temperature shifting is indicated by a long dashed grey line. The cells were cultured for a further 24 hours before one flask at 27°C (□) and one at 10°C (△) were recovered to 37°C, the remaining flasks were maintained at the previous culture temperatures. The timepoint of culture temperature recovered is indicated by a short grey dashed line.

The growth data for the CHOK1 and CHO-S cells under the different conditions is reported in Figure 1. The CHO-S suspension cells cultured at 37°C reached the maximum viable cell concentration of 8.75x10^6^ cells/ml at 120 h and remained viable (91.4%) at 144 h (Figure 1 C+D). By 216 h the culture had reached the death/decline phase with 16.4% viability. When the cells were cold-shocked at 72 h during mid-exponential phase of growth a noticeable drop in culture growth was detected with lower maximum viable cell concentrations obtained (4.45x10^6^ cells/ml at 27°C and 3.64x10^6^ cells/ml at 10°C). The maximum growth value for cells cultured at 27°C was recorded at 144 h, 24 h later than in the cells cultured at 37°C and 10°C. Cold-shocked cells remained in the stationary phase of growth and viable (>90%) at the end of the time-course at 216 h. The growth curves for the cells cold-shocked for 24 h and then re-warmed to 37°C sat above the cold-shocked cells and below the 37°C cultured cells with the maximum viable cell concentrations at 144 h of 6.95x10^6^ cells/ml when re-warmed after cold shock at 27°C and 7.35x10^6^ cells/ml in cells recovered after 10°C for 24 h. At the end of the time course the viability of the re-warmed cells had dropped to 39.4% (27°C) and 43.3% (10°C) and the growth profile was in the decline phase but growth had not deteriorated to the same extent as the cells cultured continuously at 37°C. In summary, when the temperature of culture was reduced a drop in cell growth was detected although the cells remained viable for a longer time period. This is thought to be due to fewer cells utilising less nutrients and producing less toxic by-products. Upon re-warming of cold-shocked cells to 37°C the cells appeared to recover their growth although not to the extent reached by cells continuously cultured at 37°C. Further there was a 24 h delay in reaching maximum cell growth.

The CHOK1 adherent cells did not reach as high cell growth numbers as the CHO-S cells, due to contact inhibition of the cells on the surface and limitations of the surface area, with cells cultured at 37°C reaching 0.86x10^6^ cells/ml at 72 h (Figure 1 A+B). At this time point however the viability of the cells had dropped to 38.8%, probably reflecting the process of cell removal with scraping. Trypsin could not be used to remove cells as cells needed to be quenched immediately and trypsin works optimally at 37°C and the use of trypsin requires washing steps that can compromise the metabolite profiles observed. Interestingly, when the CHOK1 cells were cold shocked higher cell growth and viability was reported compared to the 37°C control, with 27°C cells reaching 1.55x10^6^ cells/ml and 10°C cells obtaining 1.09x10^6^ cells/ml in contrast to that observed with the CHO-S cells. However, when the cells were re-warmed to 37°C the amount of viable cells dropped. Using the combined approach of chemical shift assignment of the ^1^H-^1^H TOCSY, DQF-COSY and ^1^H-^13^C HSQC experiments and published metabolites data a total of 24 components of the protein extracted CHOK1 medium were identified, and 34 identified within the CHO-S medium. 

**Figure 2:**
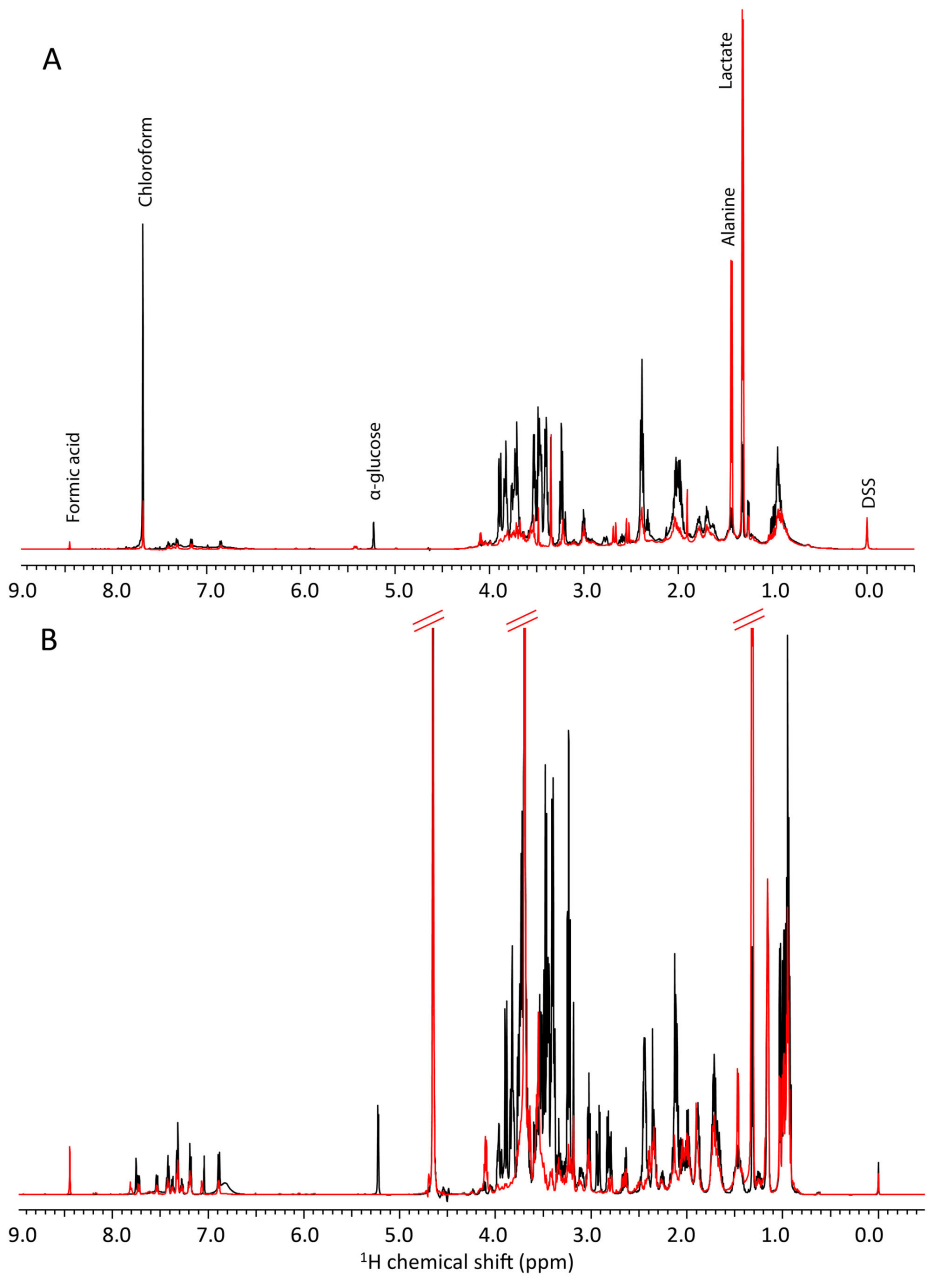
^1^H NMR spectra of cell culture media. The extracellular metabolite profile of (A) CHOK1 and (B) CHOS cultures 1D ^1^H NMR spectra. Samples taken at 0 h are shown in black, and 216 h in red. All spectra are shown on the same intensity scale.

The 0 h 1D proton spectra shown in black in Figure 2 immediately highlight the differences in composition of the two media types considering all data in Figure 2 is reported on the same intensity scale and after identical scan number. Visual observations of the CHOK1 medium (with the serum protein components of the media removed by chloroform:methanol extraction) revealed a more sparsely populated spectrum in comparison to the CHO-S medium. Each sample was spiked with the same concentration of DSS and as the volume of each peak is proportional to the total number of protons that contribute to it, it is clear that the CHO-S medium is formulated with a much higher metabolite concentration than that found in the CHOK1 medium. The protein extraction process also contributed to differences between the two media type’s spectra as after extraction the metabolites were dried to remove the large amount of methanol then re-suspended in deuterium oxide (^2^H_2_O), which cannot be seen in the ^1^H NMR spectra. As a result there is no large solvent peak at 4.65 ppm in the CHOK1 spectra. In contrast, the CHO-S medium spectra displays a residual ^1^H_2_O peak because these samples contain only 10% deuterium oxide for NMR spectrometer locking. However, the uniform presence of the DSS spike peak confirms ^1^H_2_O solvent suppression for CHO-S data does not have an adverse effect on quantitation as the scan number was identical and signal to noise comparable between CHOK1 and CHO-S data. Also, a residual chloroform peak was evident in the CHOK1 spectra at 7.68 ppm as highlighted in Figure 2A. 

Figure 2 reports the 216 h 1D proton spectra for both media types (in red). This illustrates that the majority of metabolites are used by this last time point as the peak heights are reduced. A notable example of this is for the anomeric proton of α-glucose at approximately 5.2 ppm, which is absent in both the CHOK1 and the CHO-S spectra at 216 h, despite there being a higher glucose concentration in the CHO-S medium at 0 h. Not all the metabolites identified are depleted over time, as illustrated by the increased height of the lactate and formic acid peaks in both media. Example NMR spectra of extracted cell metabolites from CHOK1 and CHOS cells cultured at 37°C collected at 72 h after seeding are shown in Figure S9 in File S1.

NMR assignments are shown in Table S5 in File S1. Of all of the media components identified, 24 CHOK1 and 29 CHO-S components were quantifiable by having had at least one proton chemical shift that did not overlap with any other metabolite present, or contained a chemical shift that overlapped with only one quantifiable metabolite, and could therefore be used for quantification purposes. The CHO-S 1D spectra contain a large volume peak at 3.66 ppm that has tentatively been assigned as the proton contribution from polyethylene glycol (PEG) a known surfactant that is added to suspension cell line media to reduce media foaming and shear stress to the cell membranes. Unlike the other identified molecules, quantification of this media component is not possible without knowing the molecular weight distribution of the polymer used and hence the number of contributing protons per molecule. 

**Figure 3:**
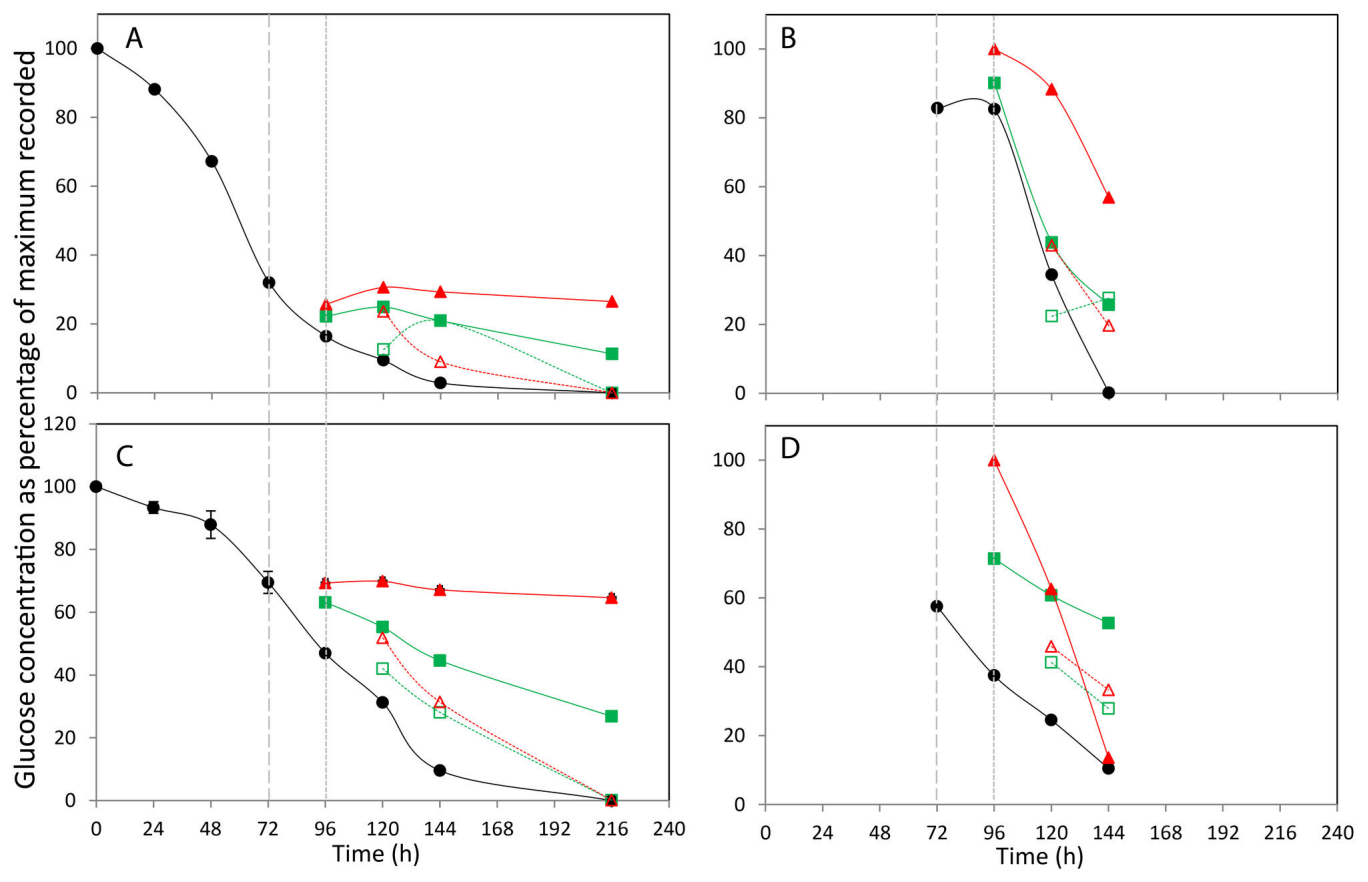
The effect of incubation temperature on extracellular (A+C) and intracellular (B+D) glucose concentration. The concentration of glucose in media (A,C) and lysed cells (B,D) for CHOK1 (A+B) and CHOS (C+D) is plotted as percentage of the maximum of each metabolite recorded within the data set. Shift in culture temperatures is denoted by the wide dashed line, and recovered temperature with the short dashed line. Legend: • 37 °C, ■ 27 °C shift, ▲ 10 °C shift, □ 27 °C recover, △ 10 °C recover.

**Figure 4:**
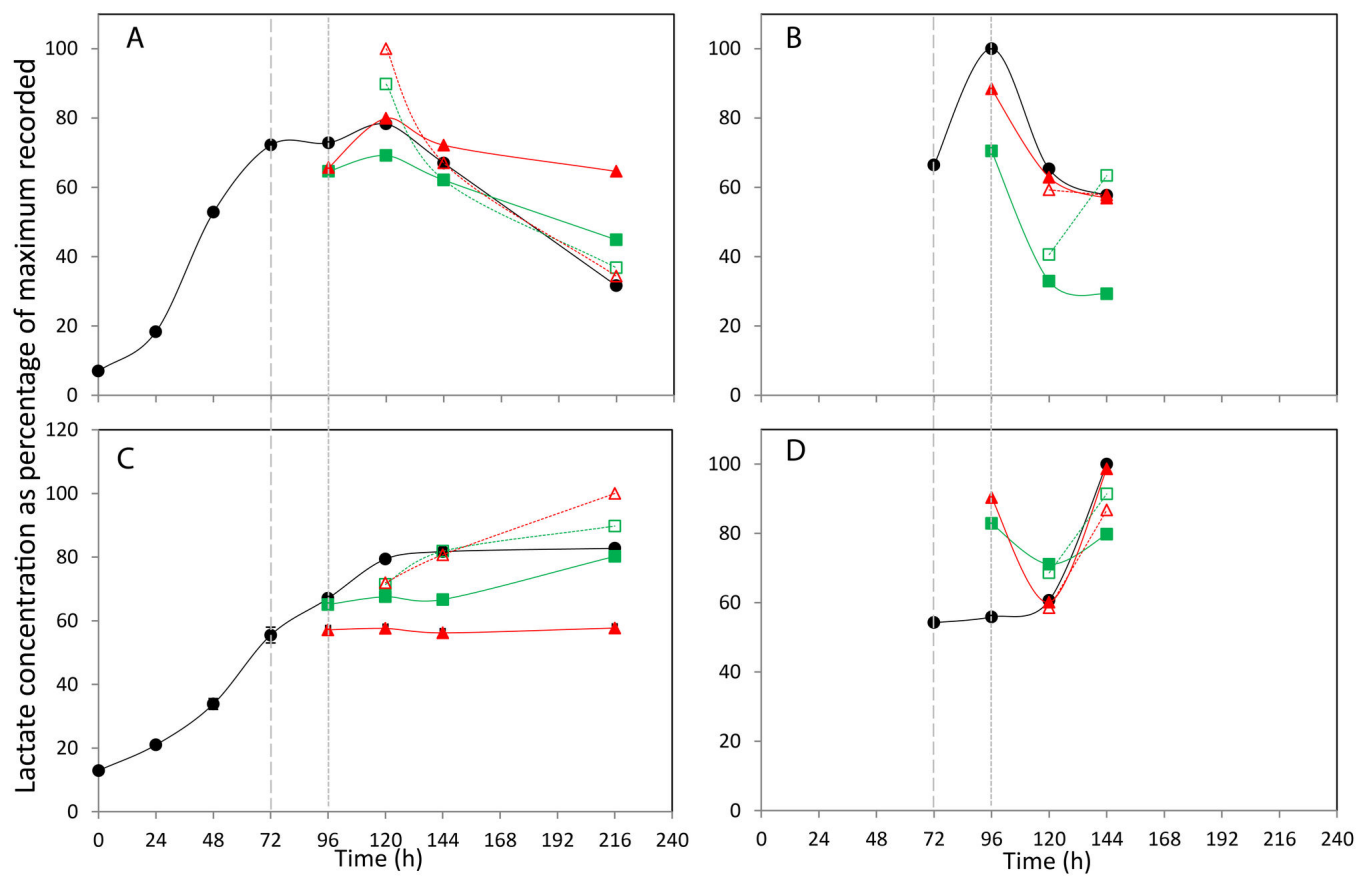
The effect of incubation temperature on extracellular (A+C) and intracellular (B+D) lactate concentration. The concentration of lactate in media (A,C) and lysed cells (B,D) for CHOK1 (A+B) and CHOS (C+D) is plotted as percentage of the maximum of each metabolite recorded within the data set. Shift in culture temperatures is denoted by the wide dashed line, and recovered temperature with the short dashed line. Legend: • 37 °C, ■ 27 °C shift, ▲ 10 °C shift, □ 27 °C recover, △ 10 °C recover.

**Figure 5:**
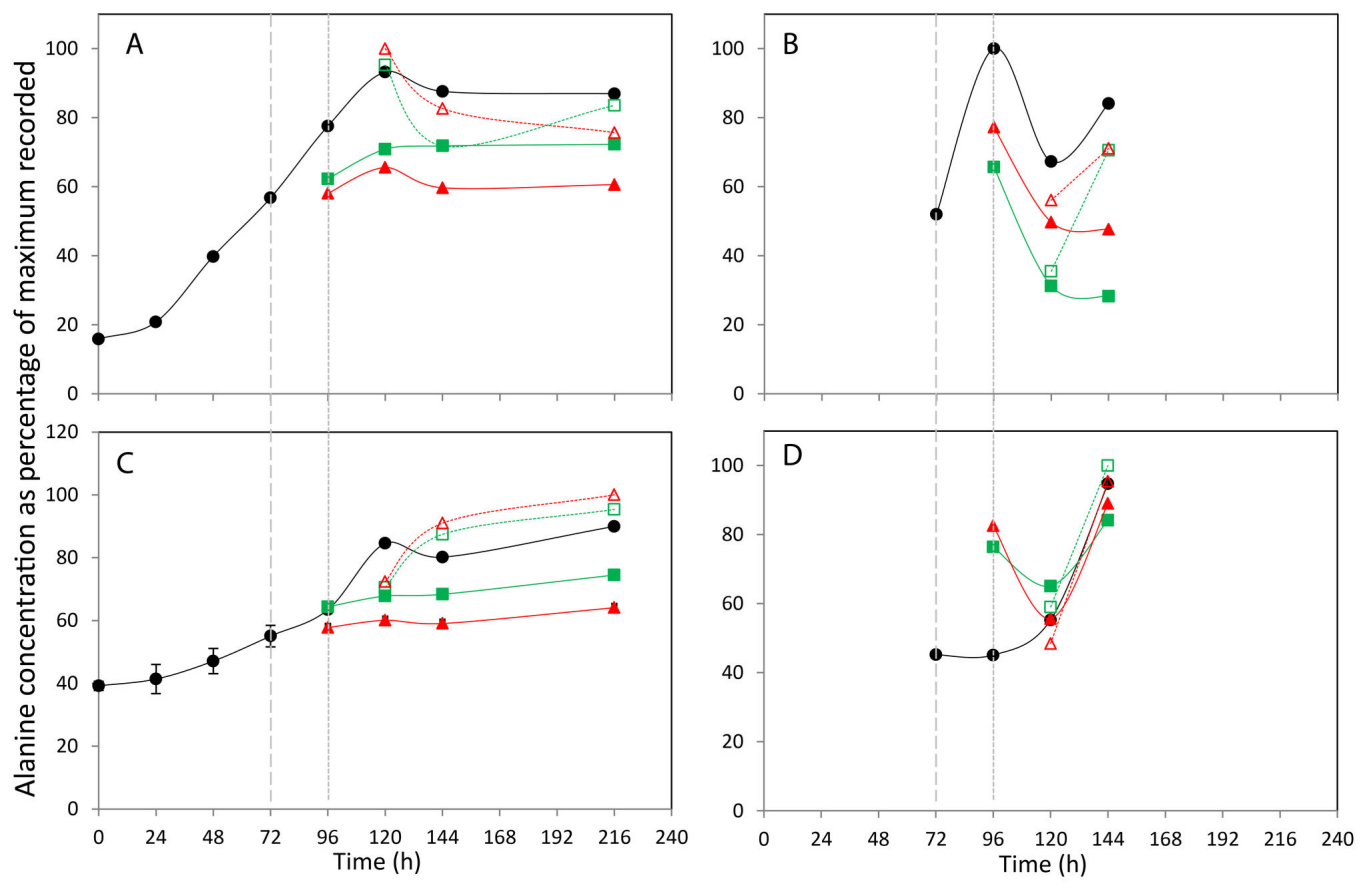
The effect of incubation temperature on extracellular (A+C) and intracellular (B+D) Alanine concentration. The concentration of alanine in media (A,C) and lysed cells (B,D) for CHOK1 (A+B) and CHOS (C+D) is plotted as percentage of the maximum of each metabolite recorded within the data set. Shift in culture temperatures is denoted by the wide dashed line, and recovered temperature with the short dashed line. Legend: • 37 °C, ■ 27 °C shift, ▲ 10 °C shift, □ 27 °C recover, △ 10 °C recover.

**Figure 6:**
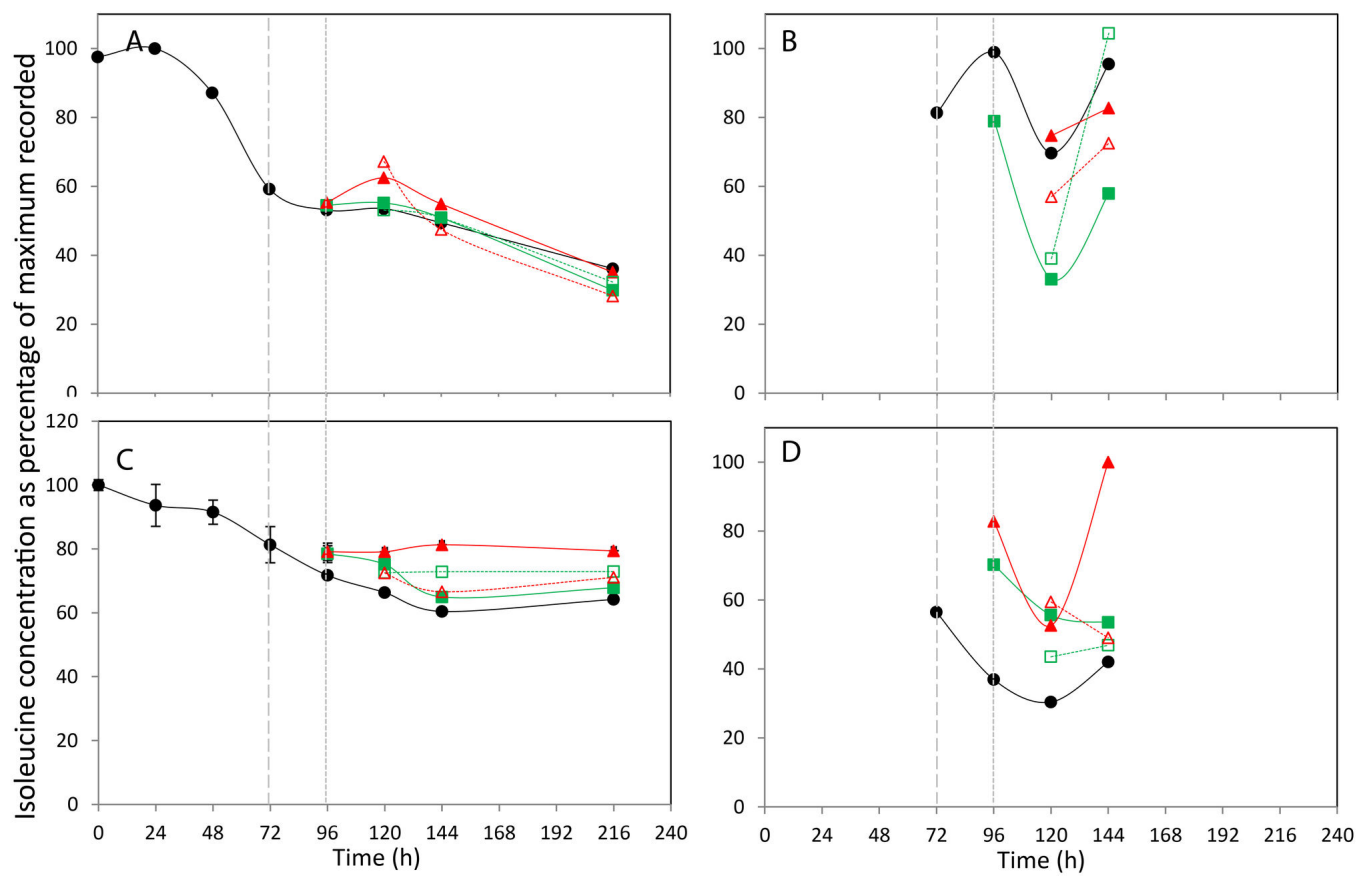
The effect of incubation temperature on extracellular (A+C) and intracellular (B+D) Isoleucine concentration. The concentration of isoleucine in media (A,C) and lysed cells (B,D) for CHOK1 (A+B) and CHOS (C+D) is plotted as percentage of the maximum of each metabolite recorded within the data set. Shift in culture temperatures is denoted by the wide dashed line, and recovered temperature with the short dashed line. Legend: • 37 °C, ■ 27 °C shift, ▲ 10 °C shift, □ 27 °C recover, △ 10 °C recover.

**Figure 7:**
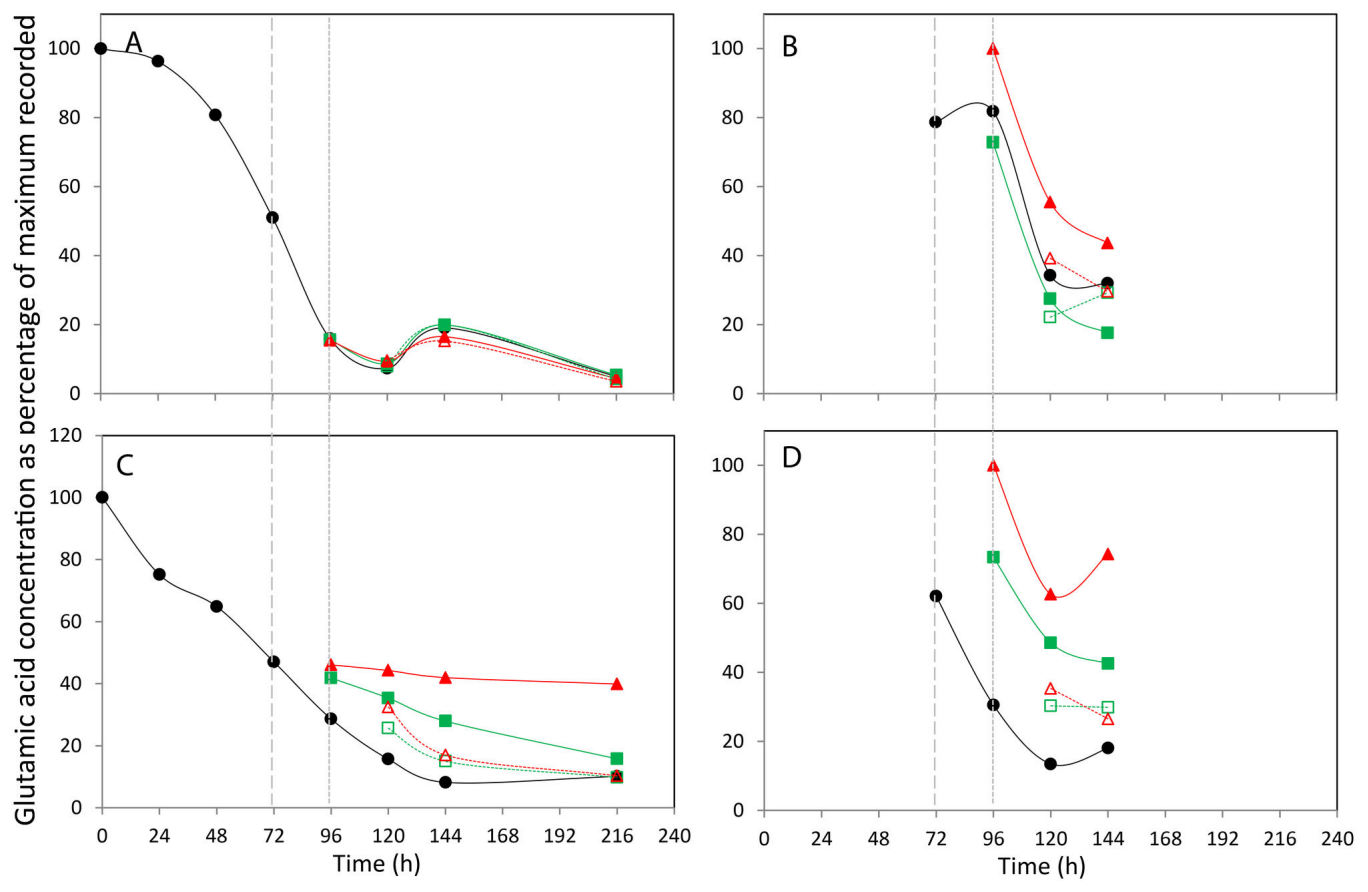
The effect of incubation temperature on extracellular (A+C) and intracellular (B+D) glutamic acid concentration. The concentration of glutamic acid in CHOK1 (A+B) and CHOS (C+D) media and lysed cells is plotted as percentage of the maximum of each metabolite recorded within the data set. Shift in culture temperatures is denoted by the wide dashed line, and recovered temperature with the short dashed line. Legend: • 37 °C, ■ 27 °C shift, ▲ 10 °C shift, □ 27 °C recover, △ 10 °C recover.

The DSS peak area within each spectra represents the equivalent of 90 μM of protons and so by knowing the number of protons within each media component that contribute to the associated peaks within the 1D spectra the molar concentration of each component can be established. The concentrations of each quantifiable metabolite identified within the extracellular media across all time points and growth conditions are listed in Tables S1, S2, S3 and S4 in File S1 with selected metabolite data shown in figures 3, 4, 5, 6, and 7 for glucose, lactate, alanine, isoleucine and glutamic acid respectively. The metabolites identified were selected based upon those that have previously been implicated as changing during mammalian cell culture and where we did observe changes. Figure 3A and 3C shows the effect of incubation temperature on the consumption of glucose over 216 h for the CHOK1 and CHO-S culture media respectively. The 37°C time course for both cell types show that all available glucose had been consumed by 216 h. The normalised data reveals a steady consumption of glucose in both cultures up to the point of the first temperature shift at 72 h. It is of note that at this first temperature shift time point more than 60% of the total glucose has been depleted from the CHOK1 media where as less that 40% of the original glucose has been used in the CHO-S medium. This reflects the large differences in the initial concentrations of 1.3 mM and 73 mM in the CHOK1 and CHO-S media respectively. 24 h after the temperature shifts similar trends of a reduction in glucose usage was observed in both media types and for both shifted temperatures, with the 10°C cultures using less glucose in comparison to the 27°C shifted cells. Cultures of both cell types show very little glucose consumption when shifted and kept at 10°C until the end of the culture and the final concentration of glucose for the 27°C shifted cultures is approximately 50% of that seen at 10°C. Data from temperature recovered cultures again follow a similar trend in both cell types. The recovery of the cells to 37°C highlights an increase in glucose demand relative to the shifted cultures with the 10°C shift and recovered cells having used less glucose than the 27°C shift and recovered cells at 120 h. By the end of the culture glucose concentrations of these cultures matches that of the 37°C control culture (i.e. all glucose depleted). 

The by-product of glucose metabolism, lactate, was quantified using one of its two proton peaks at 1.31 ppm and the normalised lactate concentration changes are plotted for both the CHOK1 and CHO-S cultures in Figure 4A and 4C respectively. The maximum recorded concentrations of lactate in both cell types was observed in the cultures shifted and recover at 10°C, with 2.1 mM at 120 h for the CHOK1 medium and 4 mM for CHO-S medium at 216 h. The general trend for lactate production over the 216 h time course differs between the two cell lines. The CHOK1 37°C culture shows and increase in lactate concentration until 120 h after which the lactate concentration begins to fall: this is mirrored in all temperature time courses, with the recycling of lactate reduced in the 10°C and 27°C shifted cultures. Interestingly the temperature shift and recover cultures both have the highest measured lactate concentration at 120 h, (24 h after the recovery to 37°C) with the 10°C sample having a slightly higher concentration than that of the 27°C sample, with both returning to levels similar to the 37°C control culture at 144 h. In contrast there is no recycling of lactate seen for the CHO-S medium. Lactate levels in the 37°C control steadily increase until 120 h at which point the concentration remained constant. Shifting the culture temperature to 27°C resulted in a reduced level of detectable lactate within the medium, and a greater reduction in lactate concentration was seen in the 10°C culture. Re-warming the temperature shifted cultures produced the highest recorded levels of lactate, with the 10°C shift and recovered culture having the most lactate present at 216 h. 

The concentration profiles for the amino acid alanine were similar for both cell lines and the normalised concentration changes are shown in Figure 5A and 5C for the CHOK1 and CHO-S cell lines respectively. The highest concentrations of alanine were observed in the CHOK1 medium at 120 h (4.7 mM), whereas the highest recorded alanine concentration in the CHO-S medium was at 216 h (3.8 mM), and as with the CHOK1 for the 10°C shift and recovered culture. As with the lactate profiles there is a steady increase in the amount of extracellular alanine over the course of the experiment in all cultures, with shifting to 10°C and 27°C reducing the overall rate of increase in metabolite concentration in comparison to the 37°C control. In the CHOK1 medium, the shift and recover cultures appear to have alanine concentrations between the control and shifted cultures, where as these cultures contained more alanine than the 37°C control in the CHO-S medium. 

The normalised concentration changes of isoleucine within the CHOK1 and CHO-S media are shown in Figure 6A and 6C respectively. Throughout the culture time approximated 0.8 mM used by the CHOK1 cells and 1.2 mM of isoleucine was used by cells within the CHO-S medium. In both 37°C cell type time courses the isoleucine concentration steadily reduces over time. The effect of temperature shifting did not produce similar trends in the two different cell lines. In the CHOK1 extracellular medium, the reduction in isoleucine concentration appears to occur at a similar rate in all culture conditions. In the CHO-S medium, the effect of temperature change is more apparent, with the shift of culture temperature to 10°C reducing isoleucine consumption and the 27°C and 10°C and 27°C shift and recover cultures all having reduced isoleucine demand. 

Finally, the normalised glutamic acid demand from culture media of the CHOK1 and CHO-S cell lines is shown in Figure 7A and 7C respectively. The maximum concentrations of glutamic acid were determined as 11 mM in the CHOK1 medium and 22 mM in the CHO-S medium, both at time point 0 h. As seen with the demand for isoleucine from the extracellular medium, there is no obvious difference in the glutamic acid usage at different culture temperatures of the CHOK1 cells. In the CHO-S cells the culture temperature resulted in reduced glutamic acid demand in the 27°C and 10°C shifted cultures, with the 10°C culture showing the lowest demand of the 5 and the shift and recover cultures having glutamic acid concentrations between those of the shifted and 37°C control cultures at 120 and 144 h until reaching the same concentration as the control temperature cultures at 216 h. 

The concentration changes within the extracellular media of glucose, lactate, alanine, isoleucine and glutamic acid were further investigated by applying curve fits to each time course. This data is shown in Table S6 in File S1 and curve fits are shown in Figures S1, S2, S3, S4, S5 and S6 in File S1. The curve fits highlight, with the exception of the glucose depletion of the CHO-S medium, that all metabolite time courses can be fitted with an increasing or decreasing sigmoidal curve. Curves of this nature suggest that changes in these components suffer from an initial lag followed by a linear production/utilisation phase that is concluded with a plateau phase, most likely as a consequence a production or consumption limit for that component. Significant differences across the media studied were observed through the sigmoid expression values *b* and *c* that are respectively defined as the Hill co-efficient and the half-point value of x between y_max_ and y_o_. The Hill coefficient, *b* demonstrated extremes between 7.4±3.2 and 0.8±0.4 for glucose 10°C shift in CHOK1 and pyruvate 10°C shift in CHO-S respectively. As a general trend for particular metabolites, Hill co-efficient values were higher for 37°C data from CHOK1 or CHO-S and lower for shift and shift-recover data. In contrast, this trend was not observed for Hill coefficient values from CHOK1 lactate and glutamate that remain fairly constant across the experimental series. The half-point *c* showed little variation between conditions for lactate, alanine and glutamate in CHOK1 cells but the same metabolites exhibited longer periods to reach their half-point in CHO-S cells, particularly when subjected to 27°C and 10°C shift-recover conditions. 

At 72, 96, 120, 144 and 168 h, the metabolite concentration within the cells (intracellularly) was also analysed. Metabolism within 1x10^7^ cells was quenched using a cold methanol and ammonium bicarbonate solution, after which the metabolites were extracted using methanol and quantified by NMR data analysis using the same techniques used for the extracellular media. A total of 22 components were identified and quantifiable in the CHOK1 extracted cells and 23 found and quantified in the CHO-S cells, with the µM concentrations of each metabolite listed in the Tables S1, S2, S3 and S4 in File S1. The normalised intracellular concentration changes for glucose, lactate, alanine, isoleucine and glutamic acid found in CHOK1 and CHO-S cultures are shown in figure 3, 4, 5, 6, and 7B and 3-7D respectively. 

The intracellular glucose concentrations (Figure 3B and 3D for CHOK1 and CHO-S respectively) show concentration changes that generally reflect the amount of glucose available in the extracellular medium, as over time the internal concentration of glucose does not remain constant but reduces. Although it may be expected that the majority of glucose intracellularly is glucose-6-phosphate, Sellick et al [12] detected both glucose and glucose-6-phosphate in intracellular CHO cell extracts using GC-MS analysis and hence although some of the glucose detected could possibly be due to contamination from the media, the trends observed suggest that we can detect and monitor intracellular glucose in CHO cells. In both the CHOK1 and CHO-S cells the effect of culture temperature shift to 27°C or 10°C increases the intracellular glucose concentration to levels above those seen in the 24 h after temperature was changed. With the colder temperature change resulting in the largest concentration increase, after 96 h the internal concentration of all cultures begin the reduce following the same trend as the 37°C control cultures. 

The intracellular lactate concentrations of CHOK1 and CHO-S show different profiles over the time course (Figure 4B and 4D respectively). There is an increased amount of internal lactate in all 96 h conditions for the CHOK1 cells over that seen at 72 h after which time all samples have a reducing amount of lactate present. At 96 h the CHO-S temperature shifted cells have a higher internal lactate concentration than the 37°C control culture, however at 120 and 144 h the different temperature cultures have more similar lactate concentration.

For the CHOK1 cells, the internal alanine concentration increases for all culture types from 72 to 96 h, and then falls at 120 h (Figure 5B). The levels of internal alanine then increase in all cultures at 37°C at 144 h but remain level for those cultures at colder temperatures. A similar pattern is repeated here for the CHOK1 internal isoleucine concentrations (Figure 6B). A different pattern is seen in the CHO-S cells with a general trend of an increase in alanine concentration of all cultures over time observed (Figure 5D). The isoleucine concentration within the CHO-S cells generally decreases over the 72 - 120 h period, with the 37°C, 27°C shift and recover and 10°C shifted cells all have an increase in concentration at 144 h (Figure 6D). Finally the internal glutamate concentrations for the CHOK1 and CHO-S cells are shown in Figure 7B and 7D respectively. The profiles of the two cell lines across all incubation temperatures follow a similar pattern of decreased concentration over time, with only the 37°C and 10°C shifted cultures of CHO-S cells having a final increase in concentration at the final time point of 144 h. 

**Figure 8:**
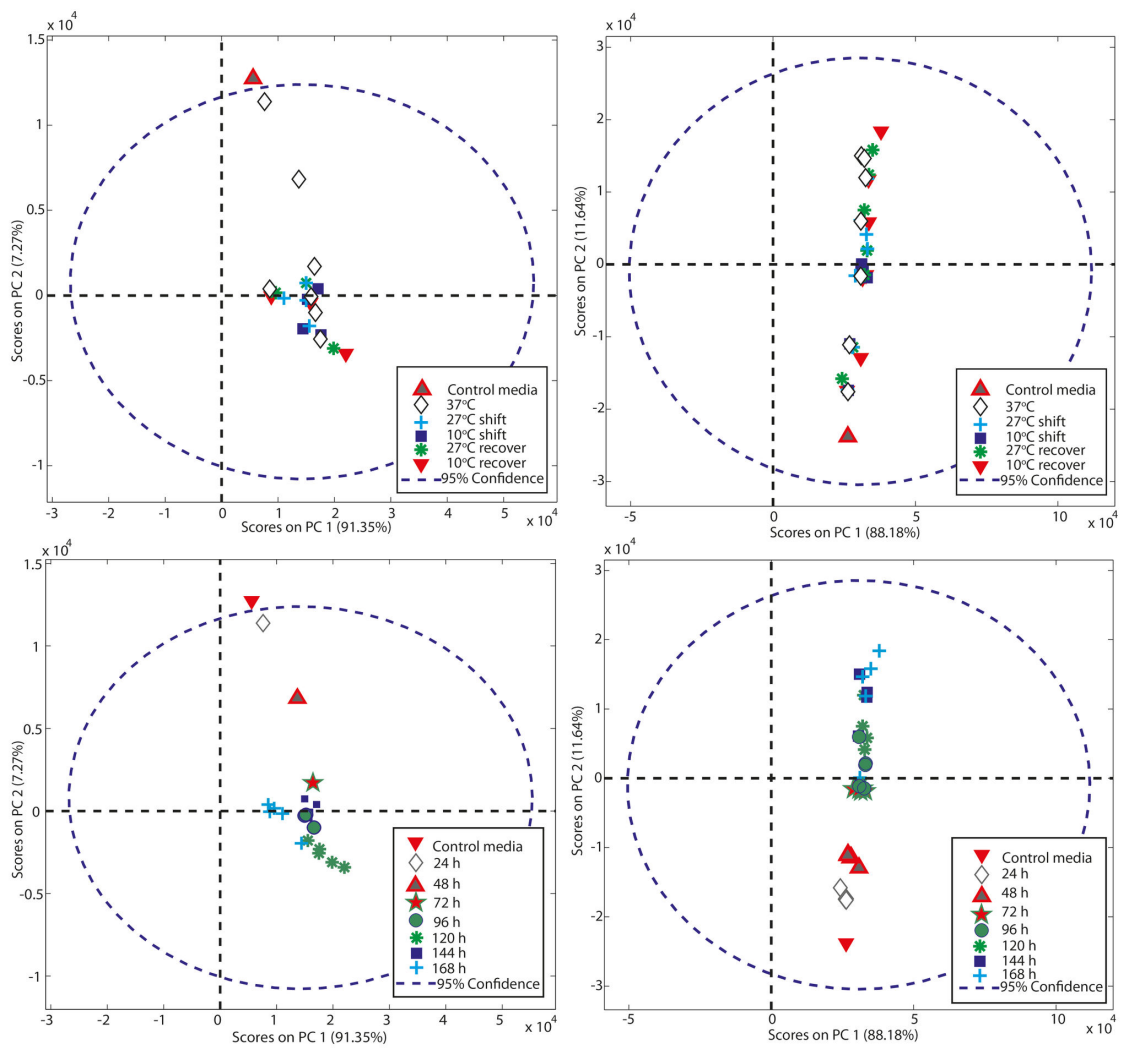
Principal component analysis of extracellular metabolites of CHOK1 (A+B) and CHOS (C+D) cultures. The analysis of the identified extracellular metabolites by PCA is plotted by either culturing conditions (A+C) or timepoint (B+D).

We also applied principal component analysis (PCA), a data compression technique, to the quantified metabolite data, to confirm the statistical rigour of our study and to identify whether any differences or similarities could be identified between the global metabolite profiles (intra- and extra-cellularly) under different culture temperature conditions. The resulting analysis depicted as bivariate scores plot of principal component 1 (PC1) versus principal component 2 (PC2) (Figure 8) showed that as a general rule the global metabolite profile clustered for a given cell line and sampling point and that as the culture temperature was reduced samples clustered away from the 37°C controls indicative of a change in the global metabolite profile. In the extra-cellular samples PC1 accounted for 88% and 91% of the variability for the CHO-S and CHOK1 samples respectively with PC2 accounting for 11.5% and 7.2% of the variability in the CHO-S and CHOK1 samples (Figure 8) and thus almost all variability was covered by PC1 and PC2. Further, there was very little separation between samples in principal component 1 (PC1) and almost all the separation between samples was in PC2 (Figure 8). For the intracellular samples >97% of the variability was in PC1 for the CHO-S cells with approximately 2.5% in PC2 whilst for the CHOK1 samples almost 99% of the variability was in PC1 with less than 1% in PC2 (Figure S7 in File S1). When cells that were cultured at lower temperatures were allowed to return to 37°C, the resulting PCA showed that these samples tended to once again cluster around those from samples at 37°C. Thus, 1D ^1^H NMR analysis and PCA can be used to fingerprint the global metabolome of a cell line under a given culture condition and could be used to identify those cell lines that respond in similar or different manners to perturbations in culture temperature.

## Discussion

Here we report on the use of an NMR based approach to investigate the extracellular cell culture media and intracellular metabolome of CHOK1 and CHO-S cells during culture and in response to cold-shock and subsequent recovery from hypothermic culturing. This is the first such study to apply an NMR metabolomic approach to the study of the metabolome of cultured cells during hypothermic recovery and the subsequent recovery from such culturing conditions. An NMR approach has previously been utilized to investigate changes in metabolism after mild hypothermic treatment of oxygen-glucose deprivation in a neonatal brain slice model of asphyxia, showing that at normal temperatures (37°C) ATP levels were severally reduced but these were restored or maintained by immediate or delayed hypothermia suggesting changes in cellular metabolism under such conditions [22]. A further study by Barba et al used NMR to investigate metabolism upon therapeutic hypothermia in rats and found that hypothermic animals showed reduced levels of alanine and lactate [23]. Our studies reported here using an NMR metabolomics approach show that cultured cells respond in a similar manner with alanine levels reduced although the lactate levels observed were CHO host cell line specific. In both cases the eventual effect was a reduction in lactate although the CHOK1 cell line showed this immediately intracellularly but this was only observed after an initial increase in lactate in the CHO-S cell line. The reprogramming of metabolism in response to temperature was therefore cell line specific as determined using such an NMR based approach.

The power of NMR for metabolomic profiling is that a range of metabolites can be simultaneous measured and monitored to generate a metabolic footprint. Previous studies investigating the extracellular metabolome of cultured cells in response to hypothermic temperature culturing have reported changes in the glucose utilization rate and lactate production [9]. Our NMR generated data reported here confirms this, validating the use of NMR for such studies. Other studies have reported that metabolic based approaches can be used to identify both intra and extracellular metabolites limiting cell growth and energy status and used this information to devise new approaches to improve cell growth and/or recombinant protein yields from in vitro cultured CHO cells [12,13]. Such studies have shown that glucose is depleted at normal culture temperatures and that feeding strategies can prolong growth and recombinant protein productivity. Here, our studies show that upon temperature downshift the intracellular concentration of glucose initially increases, suggesting the glucose transport is not compromised at lower temperatures but that as the energy demands of the cell drop in response to temperature the intracellular levels drop. Indeed, Bradley et al have also used NMR spectroscopy to quantitate metabolites in CHO cell culture medium for cells continually cultured at 37°C, their ultimate aim being the rational optimization of CHO based media to enhance therapeutic protein production [17]. 

The study of Sellick et al that utilized a GC-MS approach reported that intracellular metabolites of CHO cells from glycolysis and the TCA cycle were present during exponential phase but absent or decreased during decline and stationary phase of growth [12]. Their profiling also suggested that when cells entered stationary growth phase there was a switch in metabolism that reflects the redirection of glucose to permit continued ATP generation and maintenance of cellular redox state that ultimately limits growth and synthesis of new macromolecules but allows cell survival or maintenance [12]. To some extent this ‘reprogramming’ of metabolism is initiated here upon perception of cold-shock/sub-physiological temperatures although the two host cell lines metabolic fingerprints respond in different manners to temperature and hence manipulation of the metabolites in culture supernatant to improve growth or productivity should be tailored towards the specific cell line profile as determined by NMR analysis. This reprogramming probably reflects not only the difference in host cell but the mode of growth, suspension versus adherently growing cells. The metabolic reprogramming in response to temperature presumably supports continued cell survival and growth at 27°C, albeit at a slower rate. However, our data does suggest that this metabolic reprogramming at reduced temperature results in a change in glycolytic metabolic flux with reduced glucose depletion in the medium at reduced temperatures and generally reduced intracellular lactate observed over the time course investigated. This would suggest a reduced requirement for energy and ATP as might be expected, Interestingly however, there does appear to be an initial increase in intracellular lactate upon 27°C temperature shift which would suggest that the initial response involves reprogramming to increase glycolytic flux. 

NMR has an advantage over GC-MS based methods to monitor or detect metabolites in that it does not require derivatization of the metabolites for analysis that can introduce artefacts into the analysis. LC-MS is another potential method to investigate metabolites, does not necessarily require such derivatization and has a high sensitivity and selectivity. However, the approach does require a liquid chromatography separation step and an NMR approach does not. As such, NMR metabolite profiling is a powerful method that can complement the more routinely utilised GC-MS and LC-MS analysis techniques, although the NMR approach does require potentially more expensive and less accessible equipment and expertise. Further, with flow through NMR based instrumentation available we suggest that NMR could be used to monitor the extracellular metabolic flux during the culturing of suspension cells in real time to aid in the development of culture conditions and feeding regimes that meet the needs of the cells (here methanol:chloroform extraction is not needed as culture media are typically serum free). Even intracellular metabolic flux can be determined relatively quickly via the sampling and quenching of cells before subsequent NMR analysis to identify those key metabolites whose flux is changing and either depleting or increasing (e.g. lactate) and hence measures taken to intervene and improve the environment or culture metabolite profile. Further, we show that 1D ^1^H NMR analysis can be coupled with PCA to fingerprint the metabolome of a cell line under a given culture condition. The profiles clustered in response to temperature and recovery from temperature shock and such analysis could be used to identify those cell lines that respond in similar or different manners to perturbations in culture temperature. Such an approach will undoubtedly aid in our understanding of the metabolic reprogramming of cultured cells in response to temperature, but also throughout culture and in response to a variety of stresses. 

## Supporting Information

File S1 **File S1 includes Tables S1-S8 and Figures S1-S9.** Table S1. μM concentrations of CHOK1 media metabolites. Table S2. μM concentrations of CHOK1 cell metabolites. Table S3. μM concentrations of CHOS media metabolites where duplicates were taken, standard deviation of sample is shown beneath in parentheses. Table S4. μM concentrations of CHOS cell metabolites where duplicates were taken, standard deviation of sample is shown beneath in parentheses. Table S5. Chemical shift assignments for metabolites. Table S6. Sigmoid curve fit coefficients for all CHOK1 and CHOS data. Table S7. Composition of DMEM media. Table S8. Composition of CD-CHO media. Figure S1. Sigmoid curves for Glucose in CHOK1 and CHOS Media. Figure S2. Sigmoid curves for lactate in CHOK1 and CHOS Media. Figure S3. Sigmoid curves for alanine in CHOK1 and CHOS Media. Figure S4. Sigmoid curves for isoleucine in CHOK1 and CHOS Media. Figure S5. Sigmoid curves for glutamic acid in CHOK1 and CHOS Media. Figure S6. Sigmoid curves for pyruvate in CHOS Media. Figure S7. PCA analysis of intracellular metabolites. Figure S8. Extracting protein from the CHOK1 media sample. Figure S9. 1D 1H spectra of metabolites extracted from quenched cells.
(DOCX)
