# Supplementary material for: 1H NMR Spectroscopy Profiling of Metabolic Reprogramming of Chinese Hamster Ovary Cells upon a Temperature Shift during Culture
Source: PLoS One. 2013 Oct 10;8(10):e77195. doi: 10.1371/journal.pone.0077195 (PMC3795012; doi:10.1371/journal.pone.0077195)
Supplement: File S1 — includes Tables S1-S8 and Figures S1-S9. Table S1. μM concentrations of CHOK1 media metabolites. Table S2. μM concentrations of CHOK1 cell metabolites. Table S3. μM concentrations of CHOS media metabolites where duplicates were taken, standard deviation of sample is shown beneath in parentheses. Table S4. μM concentrations of CHOS cell metabolites where duplicates were taken, standard deviation of sample is shown beneath in parentheses. Table S5. Chemical shift assignments for metabolites. Table S6. Sigmoid curve fit coefficients for all CHOK1 and CHOS data. Table S7. Composition of DMEM media. Table S8. Composition of CD-CHO media. Figure S1. Sigmoid curves for Glucose in CHOK1 and CHOS Media. Figure S2. Sigmoid curves for lactate in CHOK1 and CHOS Media. Figure S3. Sigmoid curves for alanine in CHOK1 and CHOS Media. Figure S4. Sigmoid curves for isoleucine in CHOK1 and CHOS Media. Figure S5. Sigmoid curves for glutamic acid in CHOK1 and CHOS Media. Figure S6. Sigmoid curves for pyruvate in CHOS Media. Figure S7. PCA analysis of intracellular metabolites. Figure S8. Extracting protein from the CHOK1 media sample. Figure S9. 1D 1H spectra of metabolites extracted from quenched cells. (DOCX) [file pone.0077195.s001.docx]

**Table S1: μM concentrations of CHOK1 media metabolites**

|  | Metabolite concentration / μM | | | | | | | |
| --- | --- | --- | --- | --- | --- | --- | --- | --- |
| Isoleucine | 0 h | 24 h | 48 h | 72 h | 96 h | 120 h | 144 h | 216 h |
| 37 °C | 1189.6 | 1219.5 | 1062. 8 | 722.3 | 648.2 | 652.9 | 602.9 | 440.7 |
| 27 °C Shift |  |  |  |  | 664.8 | 673.1 | 620.9 | 364.1 |
| 10 °C Shift |  |  |  |  | 674.5 | 761.9 | 669.6 | 427.9 |
| 27 °C Recover |  |  |  |  |  | 647.9 | 620.9 | 393.1 |
| 10 °C Recover |  |  |  |  |  | 819.5 | 579.2 | 343.8 |
|  |  |  |  |  |  |  |  |  |
| Leucine | 0 h | 24 h | 48 h | 72 h | 96 h | 120 h | 144 h | 216 h |
| 37 °C | 539.3 | 520.6 | 567.3 | 392.7 | 300.3 | 321.7 | 309.8 | 244.0 |
| 27 °C Shift |  |  |  |  | 312.0 | 340.0 | 327.2 | 229.0 |
| 10 °C Shift |  |  |  |  | 335.6 | 405.5 | 417.1 | 326.5 |
| 27 °C Recover |  |  |  |  |  | 336.6 | 327.2 | 235.0 |
| 10 °C Recover |  |  |  |  |  | 396.9 | 316.8 | 184.3 |
|  |  |  |  |  |  |  |  |  |
| Valine | 0 h | 24 h | 48 h | 72 h | 96 h | 120 h | 144 h | 216 h |
| 37 °C | 460.1 | 442.7 | 404.6 | 229.5 | 194.8 | 247.0 | 275.3 | 315.0 |
| 27 °C Shift |  |  |  |  | 205.0 | 239.2 | 254.0 | 242.2 |
| 10 °C Shift |  |  |  |  | 214.0 | 250.2 | 268.4 | 243.2 |
| 27 °C Recover |  |  |  |  |  | 236.9 | 254.0 | 297.8 |
| 10 °C Recover |  |  |  |  |  | 230.1 | 247.3 | 219.5 |
|  |  |  |  |  |  |  |  |  |
| Ethanol | 0 h | 24 h | 48 h | 72 h | 96 h | 120 h | 144 h | 216 h |
| 37 °C | 146.6 | 174.7 | 185.9 | 120.7 | 88.3 | 95.0 | 111.2 | 149.9 |
| 27 °C Shift |  |  |  |  | 109.1 | 107.2 | 106.4 | 112.5 |
| 10 °C Shift |  |  |  |  | 117.4 | 142.0 | 142.8 | 88.6 |
| 27 °C Recover |  |  |  |  |  | 70.4 | 106.4 | 150.5 |
| 10 °C Recover |  |  |  |  |  | 88.6 | 98.1 | 98.9 |
|  |  |  |  |  |  |  |  |  |
| Lactate | 0 h | 24 h | 48 h | 72 h | 96 h | 120 h | 144 h | 216 h |
| 37 °C | 1490.9 | 3929.7 | 11316.6 | 15480.0 | 15611.4 | 16773.9 | 14356.8 | 6773.7 |
| 27 °C Shift |  |  |  |  | 13847.4 | 14827.8 | 13310.7 | 9612 |
| 10 °C Shift |  |  |  |  | 14075.4 | 17128.8 | 15466.2 | 13845 |
| 27 °C Recover |  |  |  |  |  | 19241.1 | 13310.7 | 7883.4 |
| 10 °C Recover |  |  |  |  |  | 21431.7 | 14370 | 7360.2 |
|  |  |  |  |  |  |  |  |  |
| Alanine | 0 h | 24 h | 48 h | 72 h | 96 h | 120 h | 144 h | 216 h |
| 37 °C | 747.4 | 979.1 | 1873.2 | 2671.7 | 3650.4 | 4389.0 | 4124.4 | 4092.3 |
| 27 °C Shift |  |  |  |  | 2930.3 | 3336.3 | 3381.0 | 3402.3 |
| 10 °C Shift |  |  |  |  | 2733.0 | 3087.0 | 2810.0 | 2853.18 |
| 27 °C Recover |  |  |  |  |  | 4484.4 | 3381.0 | 3934.2 |
| 10 °C Recover |  |  |  |  |  | 4708.5 | 3891.0 | 3562.8 |
|  |  |  |  |  |  |  |  |  |
| Arginine | 0 h | 24 h | 48 h | 72 h | 96 h | 120 h | 144 h | 216 h |
| 37 °C | 370.0 | 386.1 | 315.8 | 205.6 | 307.5 | 331.2 | 154.0 | 367.9 |
| 27 °C Shift |  |  |  |  | 272.6 | 290.4 | 160.8 | 267.9 |
| 10 °C Shift |  |  |  |  | 250.4 | 249.5 | 116.2 | 287.1 |
| 27 °C Recover |  |  |  |  |  | 400.2 | 160.8 | 337.6 |
| 10 °C Recover |  |  |  |  |  | 326.0 | 164.5 | 260.8 |
|  |  |  |  |  |  |  |  |  |
| Glutamine | 0 h | 24 h | 48 h | 72 h | 96 h | 120 h | 144 h | 216 h |
| 37 °C | 7400.3 | 6633.1 | 4317.7 | 2046.2 | 3672.1 | 2732.0 | 4528.0 | 3745.4 |
| 27 °C Shift |  |  |  |  | 4197.9 | 2848.2 | 5423.5 | 4234.3 |
| 10 °C Shift |  |  |  |  | 4493.5 | 2835.4 | 6641.4 | 2780.0 |
| 27 °C Recover |  |  |  |  |  | 2847.5 | 5423.5 | 4119.4 |
| 10 °C Recover |  |  |  |  |  | 3093.3 | 4529.6 | 3557.1 |
|  |  |  |  |  |  |  |  |  |
|  |  |  |  |  |  |  |  |  |
|  |  |  |  |  |  |  |  |  |
| Methinonine | 0 h | 24 h | 48 h | 72 h | 96 h | 120 h | 144 h | 216 h |
| 37 °C | 214.5 | 204.0 | 298.1 | 443.0 | 205.9 | 126.6 | 96.3 | 86.6 |
| 27 °C Shift |  |  |  |  | 181.9 | 137.9 | 101.3 | 70.6 |
| 10 °C Shift |  |  |  |  | 247.4 | 228.1 | 125.6 | 108.2 |
| 27 °C Recover |  |  |  |  |  | 230.7 | 101.3 | 82.7 |
| 10 °C Recover |  |  |  |  |  | 211.8 | 110.3 | 66.6 |
|  |  |  |  |  |  |  |  |  |
| glutamic acid | 0 h | 24 h | 48 h | 72 h | 96 h | 120 h | 144 h | 216 h |
| 37 °C | 11178.0 | 10762.2 | 9020.7 | 5702.8 | 1785.5 | 818.6 | 2130.6 | 559.6 |
| 27 °C Shift |  |  |  |  | 1751.6 | 966.3 | 2228.9 | 602.3 |
| 10 °C Shift |  |  |  |  | 1726.7 | 1045.4 | 1845.5 | 495.9 |
| 27 °C Recover |  |  |  |  |  | 886.6 | 2228.9 | 474.9 |
| 10 °C Recover |  |  |  |  |  | 1067.4 | 1703.9 | 399.7 |
|  |  |  |  |  |  |  |  |  |
| Aspartic acid | 0 h | 24 h | 48 h | 72 h | 96 h | 120 h | 144 h | 216 h |
| 37 °C | 1020.0 | 1015.1 | 1269.0 | 922.3 | 926.2 | 1181.1 | 1134.7 | 1525.6 |
| 27 °C Shift |  |  |  |  | 847.7 | 1039.2 | 1033.1 | 1103.0 |
| 10 °C Shift |  |  |  |  | 813.1 | 1007.4 | 904.0 | 982.3 |
| 27 °C Recover |  |  |  |  |  | 1281.0 | 1033.1 | 1409.5 |
| 10 °C Recover |  |  |  |  |  | 1281.0 | 1042.9 | 1217.9 |
|  |  |  |  |  |  |  |  |  |
| Asparagine | 0 h | 24 h | 48 h | 72 h | 96 h | 120 h | 144 h | 216 h |
| 37 °C | 2256.0 | 2223.3 | 2028.7 | 1379.4 | 1491.6 | 1712.7 | 1420.0 | 1742.0 |
| 27 °C Shift |  |  |  |  | 1495.2 | 1696.1 | 1366.2 | 1473.4 |
| 10 °C Shift |  |  |  |  | 1504.2 | 1791.6 | 1408.7 | 1414.3 |
| 27 °C Recover |  |  |  |  |  | 1734.5 | 1366.2 | 1711.2 |
| 10 °C Recover |  |  |  |  |  | 1874.2 | 1339.0 | 1423.8 |
|  |  |  |  |  |  |  |  |  |
| Proline | 0 h | 24 h | 48 h | 72 h | 96 h | 120 h | 144 h | 216 h |
| 37 °C | 824.2 | 1002.2 | 854.2 | 556.9 | 529.5 | 215.1 | 302.1 | 279.8 |
| 27 °C Shift |  |  |  |  | 558.2 | 230.6 | 374.8 | 252.2 |
| 10 °C Shift |  |  |  |  | 567.6 | 269.9 | 367.4 | 349.8 |
| 27 °C Recover |  |  |  |  |  | 260.4 | 374.8 | 278.5 |
| 10 °C Recover |  |  |  |  |  | 288.0 | 339.2 | 212.3 |
|  |  |  |  |  |  |  |  |  |
| methanol | 0 h | 24 h | 48 h | 72 h | 96 h | 120 h | 144 h | 216 h |
| 37 °C | 783.7 | 702.2 | 599.0 | 206.1 | 214.2 | 335.7 | 131.4 | 753.5 |
| 27 °C Shift |  |  |  |  | 129.5 | 362.5 | 146.0 | 613.7 |
| 10 °C Shift |  |  |  |  | 165.2 | 176.8 | 148.2 | 294.8 |
| 27 °C Recover |  |  |  |  |  | 687.0 | 146.0 | 724.7 |
| 10 °C Recover |  |  |  |  |  | 805.3 | 110.9 | 693.8 |
|  |  |  |  |  |  |  |  |  |
| Threonine | 0 h | 24 h | 48 h | 72 h | 96 h | 120 h | 144 h | 216 h |
| 37 °C | 199.1 | 140.8 | 165.7 | 155.2 | 174.5 | 163.5 | 127.9 | 139.6 |
| 27 °C Shift |  |  |  |  | 159.0 | 161.6 | 138.5 | 117.0 |
| 10 °C Shift |  |  |  |  | 165.7 | 148.5 | 138.9 | 167.3 |
| 27 °C Recover |  |  |  |  |  | 215.5 | 138.5 | 122.7 |
| 10 °C Recover |  |  |  |  |  | 186.6 | 147.0 | 111.9 |
|  |  |  |  |  |  |  |  |  |
| Glucose | 0 h | 24 h | 48 h | 72 h | 96 h | 120 h | 144 h | 216 h |
| 37 °C | 1317.1 | 1160.7 | 885.1 | 421.7 | 216.2 | 124.5 | 37.7 | 0.1 |
| 27 °C Shift |  |  |  |  | 292.7 | 328.0 | 275.7 | 149.1 |
| 10 °C Shift |  |  |  |  | 339.3 | 403.4 | 386.3 | 349.1 |
| 27 °C Recover |  |  |  |  |  | 165.9 | 275.7 | 0.1 |
| 10 °C Recover |  |  |  |  |  | 310.7 | 118.8 | 0.0 |
|  |  |  |  |  |  |  |  |  |
|  |  |  |  |  |  |  |  |  |
|  |  |  |  |  |  |  |  |  |
|  |  |  |  |  |  |  |  |  |
| Tyrosine | 0 h | 24 h | 48 h | 72 h | 96 h | 120 h | 144 h | 216 h |
| 37 °C | 268.2 | 159.0 | 113.70 | 96.9 | 77.8 | 89.2 | 87.3 | 87.1 |
| 27 °C Shift |  |  |  |  | 66.1 | 82.1 | 79.4 | 62.5 |
| 10 °C Shift |  |  |  |  | 75.1 | 104.5 | 103.7 | 106.9 |
| 27 °C Recover |  |  |  |  |  | 97.2 | 79.4 | 50.6 |
| 10 °C Recover |  |  |  |  |  | 115.0 | 84.6 | 76.0 |
|  |  |  |  |  |  |  |  |  |
| Histidine | 0 h | 24 h | 48 h | 72 h | 96 h | 120 h | 144 h | 216 h |
| 37 °C | 154.4 | 61.8 | 40.8 | 34.2 | 29.2 | 29.0 | 28.9 | 36.3 |
| 27 °C Shift |  |  |  |  | 18.0 | 24.2 | 22.2 | 0 |
| 10 °C Shift |  |  |  |  | 28.0 | 21.9 | 36.8 | 59.4 |
| 27 °C Recover |  |  |  |  |  | 19.0 | 22.2 | 0 |
| 10 °C Recover |  |  |  |  |  | 26.3 | 16.6 | 13.6 |
|  |  |  |  |  |  |  |  |  |
| Phenylalanine | 0 h | 24 h | 48 h | 72 h | 96 h | 120 h | 144 h | 216 h |
| 37 °C | 176.8 | 150.0 | 123.8 | 95.7 | 78.9 | 84.0 | 79.9 | 44.6 |
| 27 °C Shift |  |  |  |  | 69.9 | 83.7 | 80.4 | 32.8 |
| 10 °C Shift |  |  |  |  | 66.6 | 89.5 | 91.3 | 103.5 |
| 27 °C Recover |  |  |  |  |  | 97.9 | 80.4 | 35.4 |
| 10 °C Recover |  |  |  |  |  | 120.0 | 59.7 | 54.1 |
|  |  |  |  |  |  |  |  |  |
| Tryptophan | 0 h | 24 h | 48 h | 72 h | 96 h | 120 h | 144 h | 216 h |
| 37 °C | 71.5 | 0 | 0 | 12.8 | 5.6 | 0 | 0 | 0 |
| 27 °C Shift |  |  |  |  | 0 | 1.3 | 3.5 | 0 |
| 10 °C Shift |  |  |  |  | 0 | 17.8 | 13.5 | 3.4 |
| 27 °C Recover |  |  |  |  |  | 11.7 | 3.5 | 0 |
| 10 °C Recover |  |  |  |  |  | 11.0 | 0 | 0 |
|  |  |  |  |  |  |  |  |  |
| chloroform | 0 h | 24 h | 48 h | 72 h | 96 h | 120 h | 144 h | 216 h |
| 37 °C | 6092.3 | 1923.2 | 720.0 | 2090.4 | 1891.5 | 1351.4 | 1782.1 | 970.4 |
| 27 °C Shift |  |  |  |  | 2266.0 | 1057.3 | 1490.7 | 1285.7 |
| 10 °C Shift |  |  |  |  | 2794.4 | 4394.7 | 3544.8 | 903.9 |
| 27 °C Recover |  |  |  |  |  | 2575.5 | 1490.7 | 1107.8 |
| 10 °C Recover |  |  |  |  |  | 2023.0 | 1133.2 | 746.7 |
|  |  |  |  |  |  |  |  |  |
| Formic acid | 0 h | 24 h | 48 h | 72 h | 96 h | 120 h | 144 h | 216 h |
| 37 °C | 10.9 | 20.1 | 60.5 | 56.4 | 74.0 | 99.1 | 112.1 | 128.1 |
| 27 °C Shift |  |  |  |  | 63.4 | 76.2 | 93.5 | 115.0 |
| 10 °C Shift |  |  |  |  | 50.9 | 62.5 | 60.0 | 50.8 |
| 27 °C Recover |  |  |  |  |  | 103.2 | 93.5 | 116.1 |
| 10 °C Recover |  |  |  |  |  | 108.6 | 94.4 | 107.9 |
|  |  |  |  |  |  |  |  |  |
| Lyscine | 0 h | 24 h | 48 h | 72 h | 96 h | 120 h | 144 h | 216 h |
| 37 °C | 595.6 | 602.0 | 442.6 | 305.3 | 273.1 | 330.3 | 401.4 | 625.4 |
| 27 °C Shift |  |  |  |  | 236.9 | 285.1 | 383.4 | 512.7 |
| 10 °C Shift |  |  |  |  | 220.4 | 248.7 | 324.0 | 564.7 |
| 27 °C Recover |  |  |  |  |  | 380.2 | 383.4 | 594.9 |
| 10 °C Recover |  |  |  |  |  | 341.8 | 396.5 | 492.8 |
|  |  |  |  |  |  |  |  |  |
| Cysteine | 0 h | 24 h | 48 h | 72 h | 96 h | 120 h | 144 h | 216 h |
| 37 °C | 546.1 | 658.4 | 706.6 | 485.4 | 438.0 | 370.9 | 382.5 | 497.5 |
| 27 °C Shift |  |  |  |  | 436.8 | 377.0 | 413.8 | 383.0 |
| 10 °C Shift |  |  |  |  | 461.9 | 437.10 | 460.9 | 406.8 |
| 27 °C Recover |  |  |  |  |  | 428.8 | 413.8 | 448.1 |
| 10 °C Recover |  |  |  |  |  | 422.4 | 409.1 | 325.0 |

**Table S2: μM concentrations of CHOK1 cell metabolites**

|  | Metabolite concentration / μM | | | | | | | |
| --- | --- | --- | --- | --- | --- | --- | --- | --- |
| Isoleucine | 0 h | 24 h | 48 h | 72 h | 96 h | 120 h | 144 h | 216 h |
| 37 °C |  |  |  | 218.5 | 265.5 | 187.1 | 256.5 |  |
| 27 °C Shift |  |  |  |  | 212.0 | 88.9 | 155.5 |  |
| 10 °C Shift |  |  |  |  | 268.6 | 200.7 | 222.2 |  |
| 27 °C Recover |  |  |  |  |  | 104.9 | 280.4 |  |
| 10 °C Recover |  |  |  |  |  | 153.1 | 194.8 |  |
|  |  |  |  |  |  |  |  |  |
| Leucine | 0 h | 24 h | 48 h | 72 h | 96 h | 120 h | 144 h | 216 h |
| 37 °C |  |  |  | 92.6 | 113.0 | 134.7 | 263.2 |  |
| 27 °C Shift |  |  |  |  | 90.7 | 74.6 | 123.9 |  |
| 10 °C Shift |  |  |  |  | 117.3 | 154.1 | 257.9 |  |
| 27 °C Recover |  |  |  |  |  | 76.3 | 240.2 |  |
| 10 °C Recover |  |  |  |  |  | 124.1 | 217.4 |  |
|  |  |  |  |  |  |  |  |  |
| Valine | 0 h | 24 h | 48 h | 72 h | 96 h | 120 h | 144 h | 216 h |
| 37 °C |  |  |  | 62.0 | 100.7 | 85.9 | 210.7 |  |
| 27 °C Shift |  |  |  |  | 71.6 | 40.8 | 74.4 |  |
| 10 °C Shift |  |  |  |  | 84.9 | 67.3 | 149.5 |  |
| 27 °C Recover |  |  |  |  |  | 49.5 | 155.8 |  |
| 10 °C Recover |  |  |  |  |  | 68.5 | 114.9 |  |
|  |  |  |  |  |  |  |  |  |
| Ethanol | 0 h | 24 h | 48 h | 72 h | 96 h | 120 h | 144 h | 216 h |
| 37 °C |  |  |  | 26.6 | 43.4 | 62.8 | 67.7 |  |
| 27 °C Shift |  |  |  |  | 32.0 | 18.6 | 27.0 |  |
| 10 °C Shift |  |  |  |  | 37.3 | 37.7 | 53.8 |  |
| 27 °C Recover |  |  |  |  |  | 30.5 | 53.1 |  |
| 10 °C Recover |  |  |  |  |  | 35.4 | 25.7 |  |
|  |  |  |  |  |  |  |  |  |
| Lactate | 0 h | 24 h | 48 h | 72 h | 96 h | 120 h | 144 h | 216 h |
| 37 °C |  |  |  | 3806.4 | 5727.6 | 3738.6 | 3302.7 |  |
| 27 °C Shift |  |  |  |  | 4036.2 | 1883.7 | 1675.8 |  |
| 10 °C Shift |  |  |  |  | 5062.2 | 3601.8 | 3255.9 |  |
| 27 °C Recover |  |  |  |  |  | 2325.5 | 3630.3 |  |
| 10 °C Recover |  |  |  |  |  | 3394.8 | 3307.5 |  |
|  |  |  |  |  |  |  |  |  |
| Alanine | 0 h | 24 h | 48 h | 72 h | 96 h | 120 h | 144 h | 216 h |
| 37 °C |  |  |  | 692.3 | 1331.0 | 895.1 | 1118.8 |  |
| 27 °C Shift |  |  |  |  | 873.8 | 416.1 | 375.8 |  |
| 10 °C Shift |  |  |  |  | 1027.6 | 662.0 | 634.7 |  |
| 27 °C Recover |  |  |  |  |  | 472.6 | 939.2 |  |
| 10 °C Recover |  |  |  |  |  | 746.3 | 945.8 |  |
|  |  |  |  |  |  |  |  |  |
| Arginine | 0 h | 24 h | 48 h | 72 h | 96 h | 120 h | 144 h | 216 h |
| 37 °C |  |  |  | 227.8 | 269.5 | 128.3 | 202.8 |  |
| 27 °C Shift |  |  |  |  | 202.8 | 72.2 | 55.1 |  |
| 10 °C Shift |  |  |  |  | 280. 1 | 164.0 | 152.1 |  |
| 27 °C Recover |  |  |  |  |  | 69.0 | 151.3 |  |
| 10 °C Recover |  |  |  |  |  | 143.8 | 155.8 |  |
|  |  |  |  |  |  |  |  |  |
| Glutamine | 0 h | 24 h | 48 h | 72 h | 96 h | 120 h | 144 h | 216 h |
| 37 °C |  |  |  | 1430.4 | 1692.5 | 950.9 | 869.1 |  |
| 27 °C Shift |  |  |  |  | 1605.0 | 753.1 | 511.2 |  |
| 10 °C Shift |  |  |  |  | 2127.9 | 1447.4 | 1002.3 |  |
| 27 °C Recover |  |  |  |  |  | 666.2 | 750.3 |  |
| 10 °C Recover |  |  |  |  |  | 1133.6 | 777.8 |  |
|  |  |  |  |  |  |  |  |  |
| Methionine | 0 h | 24 h | 48 h | 72 h | 96 h | 120 h | 144 h | 216 h |
| 37 °C |  |  |  | 44.0 | 103.4 | 16.7 | 50.8 |  |
| 27 °C Shift |  |  |  |  | 71.3 | 15.9 | 21.3 |  |
| 10 °C Shift |  |  |  |  | 86.6 | 22.4 | 31.8 |  |
| 27 °C Recover |  |  |  |  |  | 22.0 | 31.6 |  |
| 10 °C Recover |  |  |  |  |  | 25.9 | 38.0 |  |
|  |  |  |  |  |  |  |  |  |
| Glutamic acid | 0 h | 24 h | 48 h | 72 h | 96 h | 120 h | 144 h | 216 h |
| 37 °C |  |  |  | 1583.2 | 1646.6 | 690.5 | 643.7 |  |
| 27 °C Shift |  |  |  |  | 1465.5 | 553.2 | 354.5 |  |
| 10 °C Shift |  |  |  |  | 2012.6 | 1116.0 | 878.6 |  |
| 27 °C Recover |  |  |  |  |  | 446.4 | 588.6 |  |
| 10 °C Recover |  |  |  |  |  | 789.0 | 596.6 |  |
|  |  |  |  |  |  |  |  |  |
| Aspartic acid | 0 h | 24 h | 48 h | 72 h | 96 h | 120 h | 144 h | 216 h |
| 37 °C |  |  |  | 391.5 | 555.7 | 334.1 | 423.3 |  |
| 27 °C Shift |  |  |  |  | 420.9 | 188.6 | 180.2 |  |
| 10 °C Shift |  |  |  |  | 475.9 | 339.3 | 304.7 |  |
| 27 °C Recover |  |  |  |  |  | 176.2 | 314.8 |  |
| 10 °C Recover |  |  |  |  |  | 299.6 | 375.2 |  |
|  |  |  |  |  |  |  |  |  |
| Asparagine | 0 h | 24 h | 48 h | 72 h | 96 h | 120 h | 144 h | 216 h |
| 37 °C |  |  |  | 428.7 | 711.2 | 473.0 | 428.5 |  |
| 27 °C Shift |  |  |  |  | 519.5 | 275.3 | 197.4 |  |
| 10 °C Shift |  |  |  |  | 686.1 | 549.5 | 407.4 |  |
| 27 °C Recover |  |  |  |  |  | 252.1 | 360.1 |  |
| 10 °C Recover |  |  |  |  |  | 482.8 | 400.6 |  |
|  |  |  |  |  |  |  |  |  |
| Choline | 0 h | 24 h | 48 h | 72 h | 96 h | 120 h | 144 h | 216 h |
| 37 °C |  |  |  | 15.2 | 19.3 | 18.2 | 19.5 |  |
| 27 °C Shift |  |  |  |  | 15.6 | 9.2 | 10.8 |  |
| 10 °C Shift |  |  |  |  | 22.5 | 22.6 | 20.2 |  |
| 27 °C Recover |  |  |  |  |  | 8.4 | 15.4 |  |
| 10 °C Recover |  |  |  |  |  | 14.8 | 14.0 |  |
|  |  |  |  |  |  |  |  |  |
| Methanol | 0 h | 24 h | 48 h | 72 h | 96 h | 120 h | 144 h | 216 h |
| 37 °C |  |  |  | 470.5 | 3728.7 | 304.5 | 254.7 |  |
| 27 °C Shift |  |  |  |  | 536.6 | 56.6 | 163.8 |  |
| 10 °C Shift |  |  |  |  | 3020.4 | 432.5 | 396.8 |  |
| 27 °C Recover |  |  |  |  |  | 81.2 | 369.0 |  |
| 10 °C Recover |  |  |  |  |  | 276.3 | 456.1 |  |
|  |  |  |  |  |  |  |  |  |
| Threonine | 0 h | 24 h | 48 h | 72 h | 96 h | 120 h | 144 h | 216 h |
| 37 °C |  |  |  | 6.9 | 14.3 | 0 | 18.9 |  |
| 27 °C Shift |  |  |  |  | 7.1 | 4.7 | 2.2 |  |
| 10 °C Shift |  |  |  |  | 15.2 | 6.7 | 6.1 |  |
| 27 °C Recover |  |  |  |  |  | 0 | 5.4 |  |
| 10 °C Recover |  |  |  |  |  | 9.6 | 0 |  |
|  |  |  |  |  |  |  |  |  |
| Glucose | 0 h | 24 h | 48 h | 72 h | 96 h | 120 h | 144 h | 216 h |
| 37 °C |  |  |  | 80.3 | 80.0 | 33.4 | 0.2 |  |
| 27 °C Shift |  |  |  |  | 87.4 | 42.5 | 24.9 |  |
| 10 °C Shift |  |  |  |  | 97.0 | 85.6 | 55.1 |  |
| 27 °C Recover |  |  |  |  |  | 21.7 | 26.8 |  |
| 10 °C Recover |  |  |  |  |  | 41.6 | 19.1 |  |
|  |  |  |  |  |  |  |  |  |
|  |  |  |  |  |  |  |  |  |
|  |  |  |  |  |  |  |  |  |
|  |  |  |  |  |  |  |  |  |
| Tyrosine | 0 h | 24 h | 48 h | 72 h | 96 h | 120 h | 144 h | 216 h |
| 37 °C |  |  |  | 32.0 | 43.3 | 28.0 | 21.1 |  |
| 27 °C Shift |  |  |  |  | 34.4 | 12.6 | 9.1 |  |
| 10 °C Shift |  |  |  |  | 42.8 | 30.9 | 21.7 |  |
| 27 °C Recover |  |  |  |  |  | 10.2 | 17.7 |  |
| 10 °C Recover |  |  |  |  |  | 22.0 | 19.3 |  |
|  |  |  |  |  |  |  |  |  |
| Histidine | 0 h | 24 h | 48 h | 72 h | 96 h | 120 h | 144 h | 216 h |
| 37 °C |  |  |  | 11.1 | 19.7 | 13.0 | 11.4 |  |
| 27 °C Shift |  |  |  |  | 16.2 | 3.8 | 0.0 |  |
| 10 °C Shift |  |  |  |  | 14.0 | 12.4 | 3.2 |  |
| 27 °C Recover |  |  |  |  |  | 0.0 | 0.0 |  |
| 10 °C Recover |  |  |  |  |  | 5.6 | 4.8 |  |
|  |  |  |  |  |  |  |  |  |
| Phenylalaine | 0 h | 24 h | 48 h | 72 h | 96 h | 120 h | 144 h | 216 h |
| 37 °C |  |  |  | 31.0 | 39.2 | 17.7 | 26.1 |  |
| 27 °C Shift |  |  |  |  | 31.6 | 17.3 | 6.1 |  |
| 10 °C Shift |  |  |  |  | 43.4 | 29.7 | 17.3 |  |
| 27 °C Recover |  |  |  |  |  | 14.2 | 22.5 |  |
| 10 °C Recover |  |  |  |  |  | 26.5 | 20.5 |  |
|  |  |  |  |  |  |  |  |  |
| Formic acid | 0 h | 24 h | 48 h | 72 h | 96 h | 120 h | 144 h | 216 h |
| 37 °C |  |  |  | 20.5 | 29.6 | 25.0 | 31.6 |  |
| 27 °C Shift |  |  |  |  | 23.9 | 23.4 | 9.4 |  |
| 10 °C Shift |  |  |  |  | 23.2 | 23.5 | 17.5 |  |
| 27 °C Recover |  |  |  |  |  | 17.3 | 14.2 |  |
| 10 °C Recover |  |  |  |  |  | 22.7 | 31.9 |  |
|  |  |  |  |  |  |  |  |  |
| Lysine | 0 h | 24 h | 48 h | 72 h | 96 h | 120 h | 144 h | 216 h |
| 37 °C |  |  |  | 138.1 | 232.3 | 22.8 | -92.8 |  |
| 27 °C Shift |  |  |  |  | 161.1 | 39.5 | -63.5 |  |
| 10 °C Shift |  |  |  |  | 207.2 | 36.0 | -124.0 |  |
| 27 °C Recover |  |  |  |  |  | 42.2 | -108.3 |  |
| 10 °C Recover |  |  |  |  |  | 67.1 | -71.9 |  |
|  |  |  |  |  |  |  |  |  |
| Cysteine | 0 h | 24 h | 48 h | 72 h | 96 h | 120 h | 144 h | 216 h |
| 37 °C |  |  |  | 190.2 | 280.1 | 29.7 | 157.9 |  |
| 27 °C Shift |  |  |  |  | 191.7 | 19.3 | 80.5 |  |
| 10 °C Shift |  |  |  |  | 283.0 | 22.3 | 142.1 |  |
| 27 °C Recover |  |  |  |  |  | 24.0 | 109.4 |  |
| 10 °C Recover |  |  |  |  |  | 28.6 | 103.8 |  |

**Table S3: μM concentrations of CHOS media metabolites** where duplicates were taken, standard deviation of sample is shown beneath in parentheses.

|  | Metabolite concentration / μM | | | | | | | |
| --- | --- | --- | --- | --- | --- | --- | --- | --- |
| 2-methylbutanote | 0 h | 24 h | 48 h | 72 h | 96 h | 120 h | 144 h | 216 h |
| 37 °C | 137.7  (4.3) | 105.4  (58.5) | 96.0  (20.6) | 87.4  (31.5) | 108.6 | 96.3 | 139.1 | 196.5 |
| 27 °C Shift |  |  |  |  | 67.9  (35.5) | 101.1 | 83.9 | 95.6 |
| 10 °C Shift |  |  |  |  | 74.1  (27.7) | 45.0 | 83.1 | 85.2 |
| 27 °C Recover |  |  |  |  |  | 134.5 | 166.3 | 277.3 |
| 10 °C Recover |  |  |  |  |  | 47.8 | 114.3 | 203.4 |
|  |  |  |  |  |  |  |  |  |
| Butyrate | 0 h | 24 h | 48 h | 72 h | 96 h | 120 h | 144 h | 216 h |
| 37 °C | 73.7  (15.3) | 60.4  (30.9) | 60.6  (7.6) | 57.3  (16.6) | 54.9 | 60.0 | 78.6 | 96.2 |
| 27 °C Shift |  |  |  |  | 52.2  (23.7) | 71.2 | 61.9 | 84.4 |
| 10 °C Shift |  |  |  |  | 47.2  (7.3) | 36.2 | 62.6 | 54.7 |
| 27 °C Recover |  |  |  |  |  | 118.4 | 162.4 | 221.3 |
| 10 °C Recover |  |  |  |  |  | 37.9 | 83.2 | 128.8 |
|  |  |  |  |  |  |  |  |  |
| Isovalerate | 0 h | 24 h | 48 h | 72 h | 96 h | 120 h | 144 h | 216 h |
| 37 °C | 122.3 (10.4) | 111.2  (49.6) | 110.1 (18.8) | 107.4 (26.5) | 141.7 | 145.4 | 179.5 | 216.7 |
| 27 °C Shift |  |  |  |  | 95.1  (20.4) | 118.0 | 99.1 | 128.9 |
| 10 °C Shift |  |  |  |  | 90.0  (19.7) | 69.2 | 91.6 | 83.2 |
| 27 °C Recover |  |  |  |  |  | 150.6 | 197.2 | 248.8 |
| 10 °C Recover |  |  |  |  |  | 96.4 | 145.8 | 216.2 |
|  |  |  |  |  |  |  |  |  |
| Isoleucine | 0 h | 24 h | 48 h | 72 h | 96 h | 120 h | 144 h | 216 h |
| 37 °C | 3299.0 (55.8) | 3088.9 (215.7) | 3018.5 (124.2) | 2681.7 (186.6) | 2366.3 | 2188.5 | 1993.2 | 2117.0 |
| 27 °C Shift |  |  |  |  | 2587.6 (82.9) | 2480.0 | 2142.7 | 2237.2 |
| 10 °C Shift |  |  |  |  | 2609.6  (89.4) | 2609.4 | 2681.1 | 2618.8 |
| 27 °C Recover |  |  |  |  |  | 2392.7 | 2402.9 | 2404.7 |
| 10 °C Recover |  |  |  |  |  | 2390.1 | 2196.2 | 2344.2 |
|  |  |  |  |  |  |  |  |  |
| Leucine | 0 h | 24 h | 48 h | 72 h | 96 h | 120 h | 144 h | 216 h |
| 37 °C | 6802.9 (17.5) | 6371.5 (149.0) | 6338.4 (305.5) | 5779.1 (283.7) | 4794.8 | 4547.7 | 3839.0 | 4174.5 |
| 27 °C Shift |  |  |  |  | 5711.6 (1.7) | 5373.2 | 4844.6 | 4758.8 |
| 10 °C Shift |  |  |  |  | 5802.3 (156.8) | 5815.8 | 5705.1 | 5664.0 |
| 27 °C Recover |  |  |  |  |  | 4898.3 | 4751.6 | 4595.4 |
| 10 °C Recover |  |  |  |  |  | 5274.0 | 4741.8 | 4646.6 |
|  |  |  |  |  |  |  |  |  |
| Valine | 0 h | 24 h | 48 h | 72 h | 96 h | 120 h | 144 h | 216 h |
| 37 °C | 5340.0 (25.9) | 5205.7 (173.9) | 5274.8 (262.6) | 4902.1 (189.3) | 4033.2 | 3858.3 | 3177.0 | 3663.9 |
| 27 °C Shift |  |  |  |  | 4958.0 (126.8) | 4727.7 | 4355.4 | 4205.1 |
| 10 °C Shift |  |  |  |  | 4957.4  (40.1) | 5050.2 | 4771.5 | 4850.4 |
| 27 °C Recover |  |  |  |  |  | 4076.1 | 3918.9 | 3879.9 |
| 10 °C Recover |  |  |  |  |  | 4514.7 | 4025.1 | 3841.2 |
|  |  |  |  |  |  |  |  |  |
| Ethanol | 0 h | 24 h | 48 h | 72 h | 96 h | 120 h | 144 h | 216 h |
| 37 °C | 10515.8 (69.6) | 10383.0 (257.5) | 10680.2 (441.1) | 10849.0 (462.3) | 10191.6 | 14586.9 | 9801.6 | 10654.5 |
| 27 °C Shift |  |  |  |  | 10866.3 (19.5) | 11266.2 | 9904.8 | 10875.6 |
| 10 °C Shift |  |  |  |  | 10617.8 (188.6) | 11152.2 | 10935.3 | 11117.7 |
| 27 °C Recover |  |  |  |  |  | 11311.2 | 11334.3 | 11212.5 |
| 10 °C Recover |  |  |  |  |  | 14112.6 | 11049.9 | 11510.4 |
|  |  |  |  |  |  |  |  |  |
| Lactate | 0 h | 24 h | 48 h | 72 h | 96 h | 120 h | 144 h | 216 h |
| 37 °C | 5140.9 (34.8) | 8343.7 (144.2) | 13447.5 (666.9) | 22052.2 (985.8) | 26644.5 | 31557.0 | 32490.0 | 32907.0 |
| 27 °C Shift |  |  |  |  | 25872.5 (177.7) | 26854.8 | 26505.9 | 31899.0 |
| 10 °C Shift |  |  |  |  | 22715.7 (580.5) | 22893.0 | 22342.8 | 22941.9 |
| 27 °C Recover |  |  |  |  |  | 28393.5 | 32544.0 | 35691.0 |
| 10 °C Recover |  |  |  |  |  | 28608.6 | 32088.0 | 39756.0 |
|  |  |  |  |  |  |  |  |  |
| Alanine | 0 h | 24 h | 48 h | 72 h | 96 h | 120 h | 144 h | 216 h |
| 37 °C | 1506.4 (57.3) | 1589.4 (50.1) | 1809.1 (72.5) | 2114.9 (82.5) | 2437.5 | 3249.3 | 3080.1 | 3456.3 |
| 27 °C Shift |  |  |  |  | 2472.4 (20.9) | 2604.9 | 2626.3 | 2861.3 |
| 10 °C Shift |  |  |  |  | 2217.9 (43.6) | 2308.2 | 2268.6 | 2461.9 |
| 27 °C Recover |  |  |  |  |  | 2710.9 | 3358.5 | 3664.2 |
| 10 °C Recover |  |  |  |  |  | 2780.0 | 3495.3 | 3842.4 |
|  |  |  |  |  |  |  |  |  |
| Arginine | 0 h | 24 h | 48 h | 72 h | 96 h | 120 h | 144 h | 216 h |
| 37 °C | 3585.1 (56.8) | 3452.8 (178.3) | 3384.5 (154.5) | 3035.1 (131.8) | 2541.7 | 2348.5 | 1926.1 | 2240.2 |
| 27 °C Shift |  |  |  |  | 2994.9 (35.3) | 2883.4 | 2596.5 | 2558.4 |
| 10 °C Shift |  |  |  |  | 3078.7  (83.4) | 3094.7 | 3035.4 | 3065.1 |
| 27 °C Recover |  |  |  |  |  | 2494.6 | 2345.3 | 2371.4 |
| 10 °C Recover |  |  |  |  |  | 2835.5 | 2468.1 | 2423.8 |
|  |  |  |  |  |  |  |  |  |
| Glutamine | 0 h | 24 h | 48 h | 72 h | 96 h | 120 h | 144 h | 216 h |
| 37 °C | 18178.0 (245.8) | 14252.9 (496.7) | 11993.8 (815.8) | 8289.2 (552.0) | 5400.5 | 3563.1 | 2382.8 | 2987.3 |
| 27 °C Shift |  |  |  |  | 7480.6 (46.4) | 6443.6 | 5275.4 | 3919.4 |
| 10 °C Shift |  |  |  |  | 8790.5 (438.1) | 8288.4 | 8295.6 | 8103.4 |
| 27 °C Recover |  |  |  |  |  | 5154.6 | 3749.8 | 3314.2 |
| 10 °C Recover |  |  |  |  |  | 6546.8 | 4134.3 | 3295.1 |
|  |  |  |  |  |  |  |  |  |
| Methonine | 0 h | 24 h | 48 h | 72 h | 96 h | 120 h | 144 h | 216 h |
| 37 °C | 3098.8 (111.9) | 2502.3 (93.9) | 2431.4 (89.6) | 2151.8 (96.1) | 1566.7 | 1173.1 | 813.8 | 904.1 |
| 27 °C Shift |  |  |  |  | 2005.8 (26.5) | 1764.2 | 1498.1 | 1134.8 |
| 10 °C Shift |  |  |  |  | 1995.3 (3.7) | 2073.5 | 1909.3 | 1843.8 |
| 27 °C Recover |  |  |  |  |  | 1455.8 | 1120.7 | 951.4 |
| 10 °C Recover |  |  |  |  |  | 1595.0 | 1121.7 | 935.8 |
|  |  |  |  |  |  |  |  |  |
| Pyruvate | 0 h | 24 h | 48 h | 72 h | 96 h | 120 h | 144 h | 216 h |
| 37 °C | 1859.4 (29.9) | 1620.9 (12.4) | 1549.6 (95.3) | 1443.9 (59.6) | 1033.0 | 961.2 | 764.6 | 987.7 |
| 27 °C Shift |  |  |  |  | 1258.9 (14.5) | 1153.1 | 1061.9 | 975.1 |
| 10 °C Shift |  |  |  |  | 1341.7 (51.6) | 1251.1 | 1136.2 | 1037.2 |
| 27 °C Recover |  |  |  |  |  | 1101.0 | 1027.7 | 1024.3 |
| 10 °C Recover |  |  |  |  |  | 1176.7 | 1054.1 | 944.6 |
|  |  |  |  |  |  |  |  |  |
| Glutamic acid | 0 h | 24 h | 48 h | 72 h | 96 h | 120 h | 144 h | 216 h |
| 37 °C | 22009.7 (236.3) | 16536.1 (347.6) | 14278.1 (861.6) | 10350.7 (344.6) | 6306.1 | 3455.1 | 1800.7 | 2222.1 |
| 27 °C Shift |  |  |  |  | 9204.3 (144.9) | 7783.2 | 6146.6 | 3469.3 |
| 10 °C Shift |  |  |  |  | 10139.4 (450.0) | 9740.7 | 9232.2 | 8779.6 |
| 27 °C Recover |  |  |  |  |  | 5645.2 | 3300.8 | 2154.7 |
| 10 °C Recover |  |  |  |  |  | 7136.6 | 3740.9 | 2266.1 |
|  |  |  |  |  |  |  |  |  |
| Aspartic acid | 0 h | 24 h | 48 h | 72 h | 96 h | 120 h | 144 h | 216 h |
| 37 °C | 1325.4 (28.72) | 1505.6 (75.8) | 1657.2 (108.8) | 1826.6 (118.4) | 1851.8 | 2041.3 | 1731.2 | 1536.3 |
| 27 °C Shift |  |  |  |  | 1927.2 (76.7) | 2002.3 | 1985.5 | 2176.6 |
| 10 °C Shift |  |  |  |  | 1947.4 (17.9) | 1995.9 | 1938.2 | 2073.8 |
| 27 °C Recover |  |  |  |  |  | 2094.8 | 2187.5 | 1740.1 |
| 10 °C Recover |  |  |  |  |  | 2141.5 | 2297.1 | 1809.3 |
|  |  |  |  |  |  |  |  |  |
| Asparagine | 0 h | 24 h | 48 h | 72 h | 96 h | 120 h | 144 h | 216 h |
| 37 °C | 11856.6 (360.4) | 11925.7 (744.9) | 11661.1 (225.3) | 10402.6 (658.3) | 8325.4 | 7235.9 | 5960.6 | 6810.8 |
| 27 °C Shift |  |  |  |  | 10023.8 (166.0) | 9648.0 | 8434.5 | 8196.9 |
| 10 °C Shift |  |  |  |  | 10129.1 (246.2) | 10463.4 | 10374.3 | 10270.8 |
| 27 °C Recover |  |  |  |  |  | 8588.1 | 8037.5 | 7902.1 |
| 10 °C Recover |  |  |  |  |  | 8257.5 | 7285.8 | 7032.4 |
|  |  |  |  |  |  |  |  |  |
|  |  |  |  |  |  |  |  |  |
| Proline | 0 h | 24 h | 48 h | 72 h | 96 h | 120 h | 144 h | 216 h |
| 37 °C | 9194.4 (514.6) | 9583.7 (1061.4) | 9234.2 (976.0) | 8318.1 (714.3) | 6798.3 | 6734.6 | 5522.9 | 5625.6 |
| 27 °C Shift |  |  |  |  | 7494.8 (251.9) | 7531.6 | 6484.1 | 6474.2 |
| 10 °C Shift |  |  |  |  | 8607.4 (801.2) | 7887.6 | 7993.6 | 7869.2 |
| 27 °C Recover |  |  |  |  |  | 7122.9 | 7084.4 | 6437.2 |
| 10 °C Recover |  |  |  |  |  | 8654.9 | 7887.3 | 7299.6 |
|  |  |  |  |  |  |  |  |  |
| ethanolamine | 0 h | 24 h | 48 h | 72 h | 96 h | 120 h | 144 h | 216 h |
| 37 °C | 2687.8 (215.6) | 2745.6 (313.3) | 2748.5 (173.1) | 2626.8 (315.0) | 2344.9 | 2305.8 | 2010.7 | 2135.7 |
| 27 °C Shift |  |  |  |  | 2648.1 (9.3) | 2736.1 | 2381.9 | 2401.3 |
| 10 °C Shift |  |  |  |  | 2413.6 (291.2) | 2722.5 | 2666.5 | 2639.8 |
| 27 °C Recover |  |  |  |  |  | 2530.0 | 2659.3 | 2534.9 |
| 10 °C Recover |  |  |  |  |  | 1900.1 | 1843.1 | 1803.9 |
|  |  |  |  |  |  |  |  |  |
| Threonine | 0 h | 24 h | 48 h | 72 h | 96 h | 120 h | 144 h | 216 h |
| 37 °C | 503.0  (4.2) | 204.0  (118.9) | 420.3 (140.9) | 508.2 (155.0) | 557.2 | 332.4 | 192.0 | 490.8 |
| 27 °C Shift |  |  |  |  | 471.3  (5.7) | 576.2 | 534.6 | 604.7 |
| 10 °C Shift |  |  |  |  | 648.2 (201.0) | 683.1 | 385.0 | 611.2 |
| 27 °C Recover |  |  |  |  |  | 374.7 | 227.1 | 617.6 |
| 10 °C Recover |  |  |  |  |  | 450.5 | 459.0 | 398.7 |
|  |  |  |  |  |  |  |  |  |
| Hyrdoxy proline | 0 h | 24 h | 48 h | 72 h | 96 h | 120 h | 144 h | 216 h |
| 37 °C | 243.0 (38.9) | 50.3  (63.7) | 214.5 (99.3) | 336.8 (117.2) | 586.6 | 164.8 | 109.2 | 291.1 |
| 27 °C Shift |  |  |  |  | 189.5  (19.6) | 481.2 | 364.5 | 451.0 |
| 10 °C Shift |  |  |  |  | 440.6 (258.4) | 466.6 | 245.1 | 430.9 |
| 27 °C Recover |  |  |  |  |  | 226.2 | 146.9 | 374.5 |
| 10 °C Recover |  |  |  |  |  | 169.4 | 225.2 | 212.6 |
|  |  |  |  |  |  |  |  |  |
| Glucose | 0 h | 24 h | 48 h | 72 h | 96 h | 120 h | 144 h | 216 h |
| 37 °C | 7475.0 (84.9) | 6976.4 (137.6) | 6569.7 (325.8) | 5193.1 (261.5) | 3509.7 | 2334.0 | 714.7 | 0 |
| 27 °C Shift |  |  |  |  | 4719.6 (28.5) | 4130.3 | 3335.4 | 2002.6 |
| 10 °C Shift |  |  |  |  | 5181.0 (22.9) | 5221.0 | 5017.7 | 4830.2 |
| 27 °C Recover |  |  |  |  |  | 3143.1 | 2101.3 | 11.0 |
| 10 °C Recover |  |  |  |  |  | 3875.3 | 2345.7 | 7.0 |
|  |  |  |  |  |  |  |  |  |
| Tyrosine | 0 h | 24 h | 48 h | 72 h | 96 h | 120 h | 144 h | 216 h |
| 37 °C | 2210.2 (59.2) | 1932.3 (72.7) | 1923.4 (78.7) | 1665.1 (49.5) | 1183.3 | 876.3 | 590.4 | 691.4 |
| 27 °C Shift |  |  |  |  | 1528.0 (51.6) | 1356.3 | 1116.5 | 848.3 |
| 10 °C Shift |  |  |  |  | 1598.7 (13.3) | 1623.6 | 1502.9 | 1460.2 |
| 27 °C Recover |  |  |  |  |  | 1061.4 | 869.4 | 698.3 |
| 10 °C Recover |  |  |  |  |  | 1225.9 | 884.1 | 754.1 |
|  |  |  |  |  |  |  |  |  |
| Histidine | 0 h | 24 h | 48 h | 72 h | 96 h | 120 h | 144 h | 216 h |
| 37 °C | 1805.5 (4.4) | 1730.8 (71.4) | 1704.1 (85.4) | 1543.0 (79.9) | 1213.2 | 1139.0 | 914.0 | 1063.4 |
| 27 °C Shift |  |  |  |  | 1480.8 (3.8) | 1414.4 | 1241.6 | 1085.7 |
| 10 °C Shift |  |  |  |  | 1531.7 (38.3) | 1538.5 | 1455.2 | 1451.4 |
| 27 °C Recover |  |  |  |  |  | 1281.3 | 1290.3 | 1092.3 |
| 10 °C Recover |  |  |  |  |  | 1361.0 | 1242.1 | 1253.8 |
|  |  |  |  |  |  |  |  |  |
| Phenylalanine | 0 h | 24 h | 48 h | 72 h | 96 h | 120 h | 144 h | 216 h |
| 37 °C | 1658.0 (5.5) | 1481.2 (31.6) | 1493.2 (70.9) | 1341.1 (57.2) | 1050.0 | 911.8 | 696.9 | 786.7 |
| 27 °C Shift |  |  |  |  | 1298.6 (16.2) | 1194.6 | 1056.6 | 949.6 |
| 10 °C Shift |  |  |  |  | 1343.7 (30.1) | 1354.8 | 1283.9 | 1276.4 |
| 27 °C Recover |  |  |  |  |  | 1025.7 | 913.8 | 801.5 |
| 10 °C Recover |  |  |  |  |  | 1153.6 | 937.0 | 851.6 |
|  |  |  |  |  |  |  |  |  |
| Tryptophan | 0 h | 24 h | 48 h | 72 h | 96 h | 120 h | 144 h | 216 h |
| 37 °C | 2216.7 (7.6) | 1786.6 (60.4) | 2027.1 (152.0) | 2134.2 (92.4) | 1536.5 | 1237.6 | 883.8 | 927.7 |
| 27 °C Shift |  |  |  |  | 1978.5 (27.5) | 1792.6 | 1479.3 | 1147.4 |
| 10 °C Shift |  |  |  |  | 1933.7 (7.6) | 2103.4 | 1805.9 | 1665.4 |
| 27 °C Recover |  |  |  |  |  | 1515.2 | 1236.6 | 938.1 |
| 10 °C Recover |  |  |  |  |  | 1742.0 | 1253.8 | 1039.2 |
|  |  |  |  |  |  |  |  |  |
| Formic acid | 0 h | 24 h | 48 h | 72 h | 96 h | 120 h | 144 h | 216 h |
| 37 °C | 20.1  (0.4) | 97.5  (6.5) | 209.9  (9.1) | 408.8 (18.6) | 672.9 | 1130.3 | 1394.2 | 1863.7 |
| 27 °C Shift |  |  |  |  | 527.4  (10.1) | 624.1 | 696.6 | 997.6 |
| 10 °C Shift |  |  |  |  | 425.4  (1.8) | 452.6 | 448.4 | 475.9 |
| 27 °C Recover |  |  |  |  |  | 822.4 | 1333.0 | 1787.5 |
| 10 °C Recover |  |  |  |  |  | 717.7 | 1163.9 | 1659.5 |
|  |  |  |  |  |  |  |  |  |
| Nicotinamide | 0 h | 24 h | 48 h | 72 h | 96 h | 120 h | 144 h | 216 h |
| 37 °C | 24.1  (0.3) | 23.5  (1.2) | 23.1  (2.7) | 22.5  (5.1) | 15.3 | 17.8 | 18.7 | 20.3 |
| 27 °C Shift |  |  |  |  | 22.7  (1.6) | 23.2 | 22.3 | 20.9 |
| 10 °C Shift |  |  |  |  | 22.8  (4.4) | 25.3 | 17.9 | 13.3 |
| 27 °C Recover |  |  |  |  |  | 20.4 | 22.5 | 21.2 |
| 10 °C Recover |  |  |  |  |  | 25.3 | 20.6 | 14.1 |
|  |  |  |  |  |  |  |  |  |
| Cysteine | 0 h | 24 h | 48 h | 72 h | 96 h | 120 h | 144 h | 216 h |
| 37 °C | 2011.9 (219.2) | 2212.6 (152.4) | 2152.7 (259.4) | 2001.0 (125.6) | 1205.7 | 903.9 | 809.6 | 924.7 |
| 27 °C Shift |  |  |  |  | 2000.9 (82.9) | 1913.3 | 1809.2 | 1728.2 |
| 10 °C Shift |  |  |  |  | 2086.8 (156.7) | 1980.9 | 1762.2 | 1883.7 |
| 27 °C Recover |  |  |  |  |  | 1499.9 | 1314.2 | 1422.3 |
| 10 °C Recover |  |  |  |  |  | 2024.4 | 1710.6 | 1570.1 |
|  |  |  |  |  |  |  |  |  |
| Lysine | 0 h | 24 h | 48 h | 72 h | 96 h | 120 h | 144 h | 216 h |
| 37 °C | 1765.8 (397.8) | 1995.7 (343.2) | 1964.7 (211.2) | 1686.2 (323.8) | 1868.0 | 1743.9 | 1293.8 | 1830.6 |
| 27 °C Shift |  |  |  |  | 1330.1 (138.9) | 1410.6 | 1124.2 | 1118.2 |
| 10 °C Shift |  |  |  |  | 1522.3 (15.2) | 1454.9 | 1558.4 | 1485.9 |
| 27 °C Recover |  |  |  |  |  | 1314.0 | 1256.0 | 1321. 6 |
| 10 °C Recover |  |  |  |  |  | 1139.5 | 1139.0 | 1149.4 |

**Table S4: μM concentrations of CHOS cell metabolites** where duplicates were taken, standard deviation of sample is shown beneath in parentheses.

|  | Metabolite concentration / μM | | | | | | | |
| --- | --- | --- | --- | --- | --- | --- | --- | --- |
| Isoleucine | 0 h | 24 h | 48 h | 72 h | 96 h | 120 h | 144 h | 216 h |
| 37 °C |  |  |  | 220.2 (28.7) | 144.0 | 118.5 | 163.8 |  |
| 27 °C Shift |  |  |  |  | 273.8 (13.8) | 216.8 | 208.6 |  |
| 10 °C Shift |  |  |  |  | 322.7 (57.5) | 204.9 | 389.7 |  |
| 27 °C Recover |  |  |  |  |  | 169.6 | 182.7 |  |
| 10 °C Recover |  |  |  |  |  | 231.7 | 191.1 |  |
|  |  |  |  |  |  |  |  |  |
| Leucine | 0 h | 24 h | 48 h | 72 h | 96 h | 120 h | 144 h | 216 h |
| 37 °C |  |  |  | 389.0 (44.8) | 273.8 | 236.9 | 302.6 |  |
| 27 °C Shift |  |  |  |  | 492.5 (19.9) | 402.0 | 383.6 |  |
| 10 °C Shift |  |  |  |  | 622.2 (105.2) | 406.0 | 471.7 |  |
| 27 °C Recover |  |  |  |  |  | 328.5 | 328.3 |  |
| 10 °C Recover |  |  |  |  |  | 330.9 | 331.5 |  |
|  |  |  |  |  |  |  |  |  |
| Valine | 0 h | 24 h | 48 h | 72 h | 96 h | 120 h | 144 h | 216 h |
| 37 °C |  |  |  | 276.0 (35.8) | 202.7 | 175.2 | 240.1 |  |
| 27 °C Shift |  |  |  |  | 366.3 (5.9) | 290.5 | 304.1 |  |
| 10 °C Shift |  |  |  |  | 491.3 (91.8) | 315.0 | 364.8 |  |
| 27 °C Recover |  |  |  |  |  | 227.0 | 268.9 |  |
| 10 °C Recover |  |  |  |  |  | 200.2 | 257.9 |  |
|  |  |  |  |  |  |  |  |  |
| Ethanol | 0 h | 24 h | 48 h | 72 h | 96 h | 120 h | 144 h | 216 h |
| 37 °C |  |  |  | 617.1 (88.5) | 503.6 | 534.2 | 753. 8 |  |
| 27 °C Shift |  |  |  |  | 834.5 (75.3) | 678.6 | 672.8 |  |
| 10 °C Shift |  |  |  |  | 1052.1 (175.9) | 691.0 | 806.5 |  |
| 27 °C Recover |  |  |  |  |  | 603.9 | 737.9 |  |
| 10 °C Recover |  |  |  |  |  | 614.6 | 742.4 |  |
|  |  |  |  |  |  |  |  |  |
| Lactate | 0 h | 24 h | 48 h | 72 h | 96 h | 120 h | 144 h | 216 h |
| 37 °C |  |  |  | 1369.0 (165.8) | 1410.2 | 1531.0 | 2524.0 |  |
| 27 °C Shift |  |  |  |  | 2090.6 (121.8) | 1795.0 | 2012.1 |  |
| 10 °C Shift |  |  |  |  | 2278.8 (315.2) | 1517.3 | 2487.8 |  |
| 27 °C Recover |  |  |  |  |  | 1731.5 | 2306.9 |  |
| 10 °C Recover |  |  |  |  |  | 1476.6 | 2188.4 |  |
|  |  |  |  |  |  |  |  |  |
| Alanine | 0 h | 24 h | 48 h | 72 h | 96 h | 120 h | 144 h | 216 h |
| 37 °C |  |  |  | 131.3 (20.1) | 130.9 | 160.5 | 275.0 |  |
| 27 °C Shift |  |  |  |  | 221.7 (7.7) | 188.9 | 244.4 |  |
| 10 °C Shift |  |  |  |  | 239.7 (39.4) | 161.5 | 258.4 |  |
| 27 °C Recover |  |  |  |  |  | 171.3 | 290.3 |  |
| 10 °C Recover |  |  |  |  |  | 140.6 | 277.0 |  |
|  |  |  |  |  |  |  |  |  |
| Arginine | 0 h | 24 h | 48 h | 72 h | 96 h | 120 h | 144 h | 216 h |
| 37 °C |  |  |  | 179.0 (27.8) | 105.1 | 93.9 | 210.1 |  |
| 27 °C Shift |  |  |  |  | 235.5 (5.2) | 185.3 | 271.7 |  |
| 10 °C Shift |  |  |  |  | 300.2 (42.3) | 208.4 | 318.8 |  |
| 27 °C Recover |  |  |  |  |  | 139.3 | 246.7 |  |
| 10 °C Recover |  |  |  |  |  | 156.1 | 240.1 |  |
|  |  |  |  |  |  |  |  |  |
| Glutamine | 0 h | 24 h | 48 h | 72 h | 96 h | 120 h | 144 h | 216 h |
| 37 °C |  |  |  | 584.3 (87.6) | 282.2 | 169.9 | 269.3 |  |
| 27 °C Shift |  |  |  |  | 707.4 (60.2) | 523.7 | 608.8 |  |
| 10 °C Shift |  |  |  |  | 979.0 (127.5) | 650.2 | 616.6 |  |
| 27 °C Recover |  |  |  |  |  | 344.6 | 405.4 |  |
| 10 °C Recover |  |  |  |  |  | 368.5 | 368.0 |  |
|  |  |  |  |  |  |  |  |  |
| Methionine | 0 h | 24 h | 48 h | 72 h | 96 h | 120 h | 144 h | 216 h |
| 37 °C |  |  |  | 126.7 (18.7) | 81.4 | 48.8 | 58.88 |  |
| 27 °C Shift |  |  |  |  | 164.1 (4.9) | 116.0 | 100.0 |  |
| 10 °C Shift |  |  |  |  | 211.4 (54.7) | 111.7 | 170.2 |  |
| 27 °C Recover |  |  |  |  |  | 77.9 | 79.02 |  |
| 10 °C Recover |  |  |  |  |  | 89.9 | 74.5 |  |
|  |  |  |  |  |  |  |  |  |
| Glutamic acid | 0 h | 24 h | 48 h | 72 h | 96 h | 120 h | 144 h | 216 h |
| 37 °C |  |  |  | 603.8 (82.2) | 297.2 | 130.9 | 175.3 |  |
| 27 °C Shift |  |  |  |  | 712.8 (33.3) | 472.0 | 413.9 |  |
| 10 °C Shift |  |  |  |  | 971.9 (148.3) | 608.4 | 721.9 |  |
| 27 °C Recover |  |  |  |  |  | 294.6 | 290.4 |  |
| 10 °C Recover |  |  |  |  |  | 343.5 | 258.0 |  |
|  |  |  |  |  |  |  |  |  |
| Aspartic acid | 0 h | 24 h | 48 h | 72 h | 96 h | 120 h | 144 h | 216 h |
| 37 °C |  |  |  | 94.9 (20.9) | 59.2 | 43.0 | 90.4 |  |
| 27 °C Shift |  |  |  |  | 129.2 (19.9) | 102.0 | 71.0 |  |
| 10 °C Shift |  |  |  |  | 194.4 (1.7) | 124.1 | 287.0 |  |
| 27 °C Recover |  |  |  |  |  | 50.3 | 107.1 |  |
| 10 °C Recover |  |  |  |  |  | 70.8 | 102.3 |  |
|  |  |  |  |  |  |  |  |  |
| Asparagine | 0 h | 24 h | 48 h | 72 h | 96 h | 120 h | 144 h | 216 h |
| 37 °C |  |  |  | 659.2 (76.0) | 399.3 | 301.8 | 417.2 |  |
| 27 °C Shift |  |  |  |  | 812.2 (26.0) | 681.6 | 491.9 |  |
| 10 °C Shift |  |  |  |  | 886.8 (22.3) | 623.0 | 1028.8 |  |
| 27 °C Recover |  |  |  |  |  | 451.1 | 486.2 |  |
| 10 °C Recover |  |  |  |  |  | 453.7 | 511.3 |  |
|  |  |  |  |  |  |  |  |  |
| Choline | 0 h | 24 h | 48 h | 72 h | 96 h | 120 h | 144 h | 216 h |
| 37 °C |  |  |  | 44.9 (12.9) | 30.2 | 23.4 | 32.2 |  |
| 27 °C Shift |  |  |  |  | 36.9 (22.6) | 53.4 | 44.1 |  |
| 10 °C Shift |  |  |  |  | 78.4  (9.6) | 52.8 | 87.2 |  |
| 27 °C Recover |  |  |  |  |  | 8.8 | 38.6 |  |
| 10 °C Recover |  |  |  |  |  | 45.2 | 40.7 |  |
|  |  |  |  |  |  |  |  |  |
| Methanol | 0 h | 24 h | 48 h | 72 h | 96 h | 120 h | 144 h | 216 h |
| 37 °C |  |  |  | 2250.4 (743.8) | 549.6 | 188.1 | 186.6 |  |
| 27 °C Shift |  |  |  |  | 1508.5 (659.5) | 781.3 | 262.2 |  |
| 10 °C Shift |  |  |  |  | 4388.9 (2997.7) | 1154.9 | 108018.0 |  |
| 27 °C Recover |  |  |  |  |  | 430.3 | 132.1 |  |
| 10 °C Recover |  |  |  |  |  | 439.9 | 144.5 |  |
|  |  |  |  |  |  |  |  |  |
| Threonine | 0 h | 24 h | 48 h | 72 h | 96 h | 120 h | 144 h | 216 h |
| 37 °C |  |  |  | 15.3  (3.5) | 5.4 | 0 | 0 |  |
| 27 °C Shift |  |  |  |  | 17.4  (9.9) | 6.4 | 9.3 |  |
| 10 °C Shift |  |  |  |  | 7.4  (7.3) | 0.1 | 0 |  |
| 27 °C Recover |  |  |  |  |  | 1.0 | 4.4 |  |
| 10 °C Recover |  |  |  |  |  | 0 | 1.9 |  |
|  |  |  |  |  |  |  |  |  |
| Glucose | 0 h | 24 h | 48 h | 72 h | 96 h | 120 h | 144 h | 216 h |
| 37 °C |  |  |  | 189.0 (29.0) | 123.0 | 80.5 | 34.4 |  |
| 27 °C Shift |  |  |  |  | 234.4 (4.9) | 199.4 | 173.0 |  |
| 10 °C Shift |  |  |  |  | 328.4 (65.6) | 205.4 | 44.5 |  |
| 27 °C Recover |  |  |  |  |  | 135.5 | 91.5 |  |
| 10 °C Recover |  |  |  |  |  | 150.7 | 109.3 |  |
|  |  |  |  |  |  |  |  |  |
| Tyrosine | 0 h | 24 h | 48 h | 72 h | 96 h | 120 h | 144 h | 216 h |
| 37 °C |  |  |  | 44.1  (5.4) | 29.9 | 23.5 | 32.6 |  |
| 27 °C Shift |  |  |  |  | 52.1  (0.4) | 45.8 | 43.1 |  |
| 10 °C Shift |  |  |  |  | 71.5 (10.8) | 44.6 | 48.2 |  |
| 27 °C Recover |  |  |  |  |  | 33.8 | 33.0 |  |
| 10 °C Recover |  |  |  |  |  | 35.7 | 32.6 |  |
|  |  |  |  |  |  |  |  |  |
| Histidine | 0 h | 24 h | 48 h | 72 h | 96 h | 120 h | 144 h | 216 h |
| 37 °C |  |  |  | 36.5  (2.5) | 25.6 | 20.1 | 29.0 |  |
| 27 °C Shift |  |  |  |  | 48.64 (3.1) | 30.8 | 41.4 |  |
| 10 °C Shift |  |  |  |  | 57.4  (6.4) | 34.7 | 32.7 |  |
| 27 °C Recover |  |  |  |  |  | 31.8 | 32.2 |  |
| 10 °C Recover |  |  |  |  |  | 30.7 | 33.2 |  |
|  |  |  |  |  |  |  |  |  |
| Phenylalanine | 0 h | 24 h | 48 h | 72 h | 96 h | 120 h | 144 h | 216 h |
| 37 °C |  |  |  | 58.5  (5.4) | 40.4 | 34.0 | 46.9 |  |
| 27 °C Shift |  |  |  |  | 76.4  (2.8) | 66.2 | 67.8 |  |
| 10 °C Shift |  |  |  |  | 98.8 (14.8) | 64.4 | 74.7 |  |
| 27 °C Recover |  |  |  |  |  | 53.4 | 53.9 |  |
| 10 °C Recover |  |  |  |  |  | 52.3 | 52.0 |  |
|  |  |  |  |  |  |  |  |  |
| Tryptophan | 0 h | 24 h | 48 h | 72 h | 96 h | 120 h | 144 h | 216 h |
| 37 °C |  |  |  | 37.9  (5.2) | 28.8 | 21.8 | 37.7 |  |
| 27 °C Shift |  |  |  |  | 49.42 (0.7) | 43.0 | 43.5 |  |
| 10 °C Shift |  |  |  |  | 66.82 (13.3) | 43.6 | 52.7 |  |
| 27 °C Recover |  |  |  |  |  | 37.4 | 37.0 |  |
| 10 °C Recover |  |  |  |  |  | 33.2 | 41.4 |  |
|  |  |  |  |  |  |  |  |  |
| Formic acid | 0 h | 24 h | 48 h | 72 h | 96 h | 120 h | 144 h | 216 h |
| 37 °C |  |  |  | 13.0  (2.9) | 23.0 | 30.6 | 50.2 |  |
| 27 °C Shift |  |  |  |  | 18.3  (2.6) | 27.9 | 38.3 |  |
| 10 °C Shift |  |  |  |  | 22.0  (4.3) | 13.7 | 22.7 |  |
| 27 °C Recover |  |  |  |  |  | 21.9 | 44.1 |  |
| 10 °C Recover |  |  |  |  |  | 20.4 | 43.9 |  |
|  |  |  |  |  |  |  |  |  |
| Lysine | 0 h | 24 h | 48 h | 72 h | 96 h | 120 h | 144 h | 216 h |
| 37 °C |  |  |  | 115.7 (32.7) | 88.7 | 90.5 | 56.0 |  |
| 27 °C Shift |  |  |  |  | 145.3 (3.5) | 104.7 | 54.0 |  |
| 10 °C Shift |  |  |  |  | 201.8 (12.3) | 161.6 | -84.3 |  |
| 27 °C Recover |  |  |  |  |  | 78.6 | 74.2 |  |
| 10 °C Recover |  |  |  |  |  | 85.4 | 53.3 |  |
|  |  |  |  |  |  |  |  |  |
| Cysteine | 0 h | 24 h | 48 h | 72 h | 96 h | 120 h | 144 h | 216 h |
| 37 °C |  |  |  | 178.5 (16.8) | 75.4 | 52.4 | 77.7 |  |
| 27 °C Shift |  |  |  |  | 154.7 (14.9) | 126.0 | 35.6 |  |
| 10 °C Shift |  |  |  |  | 198.6 (35.1) | 110.0 | 421.4 |  |
| 27 °C Recover |  |  |  |  |  | 79.3 | 84.2 |  |
| 10 °C Recover |  |  |  |  |  | 164.7 | 100.3 |  |

**Table S5: Chemical shift assignments for metabolites**

| Metabolite | ^1^H (^13^C) chemical shift / ppm |
| --- | --- |
| Acetate | 1.899 (26.01) |
| Alanine | 1.463* (18.89), 3.704 (56.68) |
| Arginine | 1.638* (26.56), 3.231 (43.21), 3.720 (56.42) |
| Asparagine | 2.793 (38.24), 2.908* (38.25), 3.922 (54.36) |
| Aspartic acid | 2.662* (39.39), 2.805 (39.39), 3.890 (55.17) |
| Butyrate | 0.865* (14.83), 2.033 (49.96) |
| Chloroform | 7.680* |
| Choline | 3.197* (56.50) |
| Cysteine | 3.091** (31.05), 3.178 (31.00), 3.945 (54.43) |
| Ethanol | 1.155* (18.73), 3.656 (60.06) |
| Ethanol amine | 3.232 (43.33), 3.604* (57.21) |
| Formic acid | 8.444* |
| α-Glucose | 3.394 (72.33), 3.524 (74.31), 3.704 (75.67), 3.759 (63.36), 3.815 (74.09), 3.833 (63.36), 5.221* (94.78) |
| β-Glucose | 3.231 (76.94), 3.395 (72.34), 3.459 (78.53), 3.460 (78.54), 3.714 (63.52), 3.886 (63.522) |
| Glutamic acid | 2.107 (29.37), 2.423* (33.57), 2.447 (33.57), 3.732 (54.07) |
| Glutamine | 2.029* (29.78), 2.105 (29.80), 2.340 (36.17), 3.762 (54.13) |
| Glycine | 3.720 (56.42) |
| Histidine | 3.257 (29.07), 7.009*, 7.157 |
| Hydroxy proline | 2.140 (40.14), 2.411 (40.14), 3.331 (55.74), 3.472 (55.74), 4.326* (62.25) |
| Isoleucine | 0.921* (13.74), 0.991 (17.33), 1.250 (27.05), 1.460 (27.02), 1.954 (38.58), 3.643 (62.36) |
| Isovalerate | 0.885* (27.13), 2.596 (51.07) |
| Lactate | 1.3128* (22.79), 4.099 (71.03) |
| Leucine | 0.938 (23.58), 0.952* (24.66), 1.656 (42.65), 1.714 (42.66), 1.716 (26.79), 3.726 (57.72) |
| Lysine | 1.443 (24.00), 1.484 (24.00), 1.710** (29.09), 1.875 (30.53), 3.017 (42.02) |
| Methanol | 3.357* (50.20) |
| Methionine | 2.086 (32.87), 2.125* (16.56), 2.163 (32.87), 2.628 (31.54), 3.834 (52.90) |
| 2-Methylbutanote | 0.825* |
| Nicotinamide | 7.590, 8.239, 8.704, 8.929* |
| Phenylalanine | 3.087 (39.37), 3.256 (39.39), 3.947 (58.95), 7.318, 7.356, 7.414* |
| Proline | 1.995 (26.33), 2.054, (31.51), 2.329 (31.49), 3.328* (48.86), 3.406 (48.88), 4.113 (64.16) |
| Pyruvate | 2.358* (29.12) |
| Serine | 3.668 (65.10), 4.153 (61.25) |
| Succinate | 2.376 (36.13) |
| Threonine | 1.302 (22.15) 3.413 (63.52), 4.167* (60.93) |
| Tryptophan | 3.279 (29.20), 3.465 (29.20), 4.031 (58.03), 7.200, 7.272, 7.536*, 7.734 |
| Tyrosine | 3.017 (38.46), 3.172 (38.44), 6.869*, 7.173 |
| Valine | 0.973 (19.33), 1.025* (20.72), 2.249 (31.82), 3.583 (63.15) |

Shifts marked with an * denote the chemical shifts used in metabolite concentration evaluation. Where available, shifts within parentheses show the ^13^C atom bonded to the preceding proton. Chemical shifts marked ** were those that overlapped with one other, from a metabolite that could be quantified and so concentration determined by subtraction, leucine concentration subtracted from Lysine and Phenylalanine subtracted from cysteine.

**Table S6:** Sigmoid curve fit coefficients for all CHOK1 and CHOS data

|  |  |  | **CHOK1** |  |  |  | **CHOS** |  |  |
| --- | --- | --- | --- | --- | --- | --- | --- | --- | --- |
| **Metabolite** | **conditions** | **y_o_** | **a** | **b** | **c** | **y_o_** | **a** | **b** | **c** |
| **Glucose** | **37°C** | 1280±30 | -1310±60 | 3.1±0.4 | 60.2±2.5 | **7660±160** | **-158390** | **1.4±0.3** | **1229±6676** |
|  | **27°C shift** | 1280±60 | -1080±100 | 3.5±1.0 | 53.2±4.3 | **7480±330** | **-8820±430** | **1.5±0.5** | **157±100** |
|  | **10°C shift** | 1240±40 | -875±60 | 7.4±3.2 | 50.4±2.3 | **7580±260** | **-3630±1411** | **1.2±0.6** | **75±50** |
|  | **27°C R** | 1300±90 | -1265±60 | 2.6±0.8 | 57.7±6.9 | **7230±240** | **-10663±2600** | **2.1±0.5** | **153±35** |
|  | **10°C R** | 1320±76 | -1380±170 | 2.2±0.6 | 62.6±8.3 | **7310±380** | **-11280±5310** | **2.3±0.9** | **165±68** |
|  |  |  |  |  |  |  |  |  |  |
| **Lactate** | **37°C** | 1665±1140 | 14246±1430 | 3.9±1.3 | 38.3±4.4 | 5800±880 | 31550±3575 | 2.7±0.5 | 73.3±6.7 |
|  | **27°C shift** | 1630±1211 | 12820±1460 | 4.3±1.5 | 35.0±4.6 | 6061±1168 | 22470±2650 | 3.3±1.0 | 58.5±5.4 |
|  | **10°C shift** | 1538±1249 | 14536±1676 | 3.6±1.2 | 38.4±4.9 | 5620±1280 | 18860±3290 | 2.7±1.1 | 53.4±9.5 |
|  | **27°C R** | 1605±2690 | 14290±3520 | 3.7±2.8 | 38.2±10.5 | 5180±1345 | 32470±6490 | 2.2±0.6 | 72.6±14.2 |
|  | **10°C R** | 1520±3295 | 15790±4850 | 3.2±3.9 | 41.0±13.4 | 4930±1460 | 41153±17034 | 1.6±0.6 | 98.8±51.6 |
|  |  |  |  |  |  |  |  |  |  |
| **Alanine** | **37°C** | 840±210 | 3505±370 | 3.6±1.0 | 64.9±5.8 | 1670±110 | 1801±245 | 4.6±1.6 | 95.3±8.3 |
|  | **27°C shift** | 745±75 | 2755±125 | 2.9±0.4 | 54.7±2.5 | 1540±74 | 1370±175 | 3.1±0.9 | 81.7±8.4 |
|  | **10°C shift** | 800±120 | 2120±165 | 4.2±1.3 | 46.9±3.3 | 1495±85 | 900±145 | 3.4±1.6 | 59.3±8.4 |
|  | **27°C R** | 780±450 | 3295±850 | 2.9±1.9 | 63.3±16.4 | 1495±115 | 2790±670 | 2.3±0.7 | 122±26.0 |
|  | **10°C R** | 830±532 | 3220±950 | 3.3±2.6 | 63.9±16.4 | 1570±150 | 2807±784 | 3.0±1.2 | 125.2±25.8 |
|  |  |  |  |  |  |  |  |  |  |
| **Isoleucine** | **37°C** | 1220±60 | -764±102 | 3.5±1.4 | 63.1±7.9 | 3330±60 | -1325±115 | 3.6±0.8 | 71.7±5.1 |
|  | **27°C shift** | 1220±90 | -900±280 | 2.1±1.1 | 80.5±25.0 | 3300±130 | -1068±215 | 3.0±1.7 | 54.2±10.4 |
|  | **10°C shift** | 1230±105 | -790±290 | 2.2±1.5 | 73.3±26.6 | 3320±150 | -750±240 | 2.0±1.6 | 44.4±18.0 |
|  | **27°C R** | 1230±80 | -805±180 | 2.5±1.2 | 70.9±14.5 | 3325±210 | -1150±175 | 0.4±1.4 | 71.3±10.2 |
|  | **10°C R** | 1230±130 | -1255±1195 | 1.5±1.3 | 133±165 | 3170±130 | -882±235 | 4.4±3.4 | 74.7±14.7 |
|  |  |  |  |  |  |  |  |  |  |
| **Glutamate** | **37°C** | 10900±560 | -10080±880 | 5.0±1.5 | 68.2±4.6 | 21850±1400 | -23930±4450 | 1.6±0.5 | 65.7±16.3 |
|  | **27°C shift** | 10900±570 | -9996±890 | 5.0±1.5 | 67.8±4.6 | 22100±770 | -27955±8235 | 1.0±0.3 | 109±66 |
|  | **10°C shift** | 10915±480 | -10190±760 | 4.9±1.2 | 68.4±3.9 | 21590±390 | -13380±670 | 1.7±0.2 | 33.4±2.5 |
|  | **27°C R** | 10900±590 | -10090±920 | 4.9±1.5 | 68.1±4.8 | 21600±1200 | -32980±16240 | 1.0±0.4 | 137±133 |
|  | **10°C R** | 10925±460 | -10300±730 | 4.8±1.1 | 68.7±3.8 | 21985±1520 | -30090±15385 | 1.2±0.5 | 116±105 |
|  |  |  |  |  |  |  |  |  |  |
| **Pyruvate** | **37°C** | N/A | N/A | N/A | N/A | 1815±1125 | -955±285 | 2.1±1.2 | 61.0±20.0 |
|  | **27°C shift** | N/A | N/A | N/A | N/A | 1865±48 | -1272±365 | 1.2±0.4 | 103.4±48.8 |
|  | **10°C shift** | N/A | N/A | N/A | N/A | 1803±52 | -2219±3626 | 0.8±0.4 | 466±1616 |
|  | **27°C R** | N/A | N/A | N/A | N/A | 1845±100 | -1165±590 | 1.4±0.8 | 94.9±70.6 |
|  | **10°C R** | N/A | N/A | N/A | N/A | 1785±72 | -970±265 | 2.8±1.2 | 102.4±21.9 |

Red data for media CHOS glucose highlights the poor sigmoidal fit tha can also be seen in data S7

**Table S7: Composition of DMEM media**

Below is listed the composition of the DMEM F-12 media used for culturing of the CHOK1 cells as reported by the manufacturer Invitrogen. Where the concentration a component has been determined by NMR it has also been listed.

| **Components** | **mM (reported)** | **mM (by NMR)** |
| --- | --- | --- |
| Glycine | 0.25 |  |
| L-Alanine | 0.05 | 0.075 |
| L-Arginine hydrochloride | 0.699 | 0.370 |
| L-Asparagine-H_2_O | 0.05 |  |
| L-Aspartic acid | 0.05 | 1.000 |
| L-Cysteine hydrochloride-H_2_O | 0.0998 |  |
| L-Cystine 2HCl | 0.1 | 0.546 |
| L-Glutamic Acid | 0.05 |  |
| L-Histidine hydrochloride-H_2_O | 0.15 | 0.154 |
| L-Isoleucine | 0.416 | 1.189 |
| L-Leucine | 0.451 | 0.539 |
| L-Lysine hydrochloride | 0.499 | 0.595 |
| L-Methionine | 0.116 |  |
| L-Phenylalanine | 0.215 | 0.176 |
| L-Proline | 0.15 |  |
| L-Serine | 0.25 |  |
| L-Threonine | 0.449 | 0.200 |
| L-Tryptophan | 0.0442 | 0.076 |
| L-Tyrosine disodium salt dihydrate | 0.214 | 0.270 |
| L-Valine | 0.452 | 0.460 |
| Biotin | 0.0000143 |  |
| Choline chloride | 0.0641 |  |
| D-Calcium pantothenate | 0.0047 |  |
| Folic Acid | 0.00601 |  |
| Niacinamide | 0.0166 |  |
| Pyridoxine hydrochloride | 0.00971 |  |
| Riboflavin | 0.000582 |  |
| Thiamine hydrochloride | 0.00644 |  |
| Vitamin B12 | 0.000502 |  |
| i-Inositol | 0.07 |  |
| Calcium Chloride (CaCl_2_) (anhyd.) | 1.05 |  |
| Cupric sulfate (CuSO_4_-5H_2_O) | 0.0000052 |  |
| Ferric Nitrate (Fe(NO_3_)_3_"9H_2_O) | 0.000124 |  |
| Ferric sulfate (FeSO_4_-7H_2_O) | 0.0015 |  |
| Magnesium Chloride (anhydrous) | 0.301 |  |
| Magnesium Sulfate (MgSO_4_) (anhyd.) | 0.407 |  |
| Potassium Chloride (KCl) | 4.16 |  |
| Sodium Bicarbonate (NaHCO_3_) | 14.29 |  |
| Sodium Chloride (NaCl) | 120.61 |  |
| Sodium Phosphate dibasic (Na_2_HPO_4_) anhydrous | 0.5 |  |
| Sodium Phosphate monobasic (NaH_2_PO4-H_2_O) | 0.453 |  |
| Zinc sulfate (ZnSO_4_-7H_2_O) | 0.0015 |  |
| D-Glucose (Dextrose) | 17.51 |  |
| Hypoxanthine Na | 0.015 |  |
| Linoleic Acid | 0.00015 |  |
| Lipoic Acid | 0.00051 |  |
| Phenol Red | 0.0215 |  |
| Putrescine 2HCl | 0.000503 |  |
| Sodium Pyruvate | 0.5 |  |
| Thymidine | 0.00151 |  |

**Table S8: Composition of CD-CHO media**

Below is listed the composition of the CD-CHO media used for culturing of the CHOS cells as determined by NMR. The composition of CD-CHO media is not reported by the manufacturer Invitrogen.

| **Components** | **mM (by NMR)** |  |
| --- | --- | --- |
| L-Alanine | 1.506 |  |
| L-Arginine hydrochloride | 3.585 |  |
| L-Asparagine-H_2_O | 11.856 |  |
| L-Aspartic acid | 1.325 |  |
| L-Cysteine | 2.011 |  |
| L-Glutamic Acid | 22.009 |  |
| L- Glutamine | 18.178 |  |
| L-Histidine | 1.805 |  |
| L-Isoleucine | 3.299 |  |
| L-Leucine | 6.803 |  |
| L-Lysine | 1.765 |  |
| L-Methionine | 3.098 |  |
| L-Phenylalanine | 1.658 |  |
| L-Proline | 9.194 |  |
| L-Threonine | 0.503 |  |
| L-Tryptophan | 2.216 |  |
| L-Tyrosine disodium salt dihydrate | 2.210 |  |
| L-Valine | 5.340 |  |
| Niacinamide | 0.024 |  |
| 2-Methyl butanote | 0.138 |  |
| Butyrate | 0.073 |  |
| Isovalerate | 0.122 |  |
| Glucose | 7.475 |  |
| Lactate | 5.140 |  |
| Pyruvate | 1.859 |  |
| Ethanolamine | 2.687 |  |
| Hydroxyproline | 0.243 |  |
| Polyethylene glycol | Not quantified |  |
| Formic Acid | 0.020 |  |

**Figure S1:** Sigmoid curves for Glucose in CHOK1 and CHOS Media


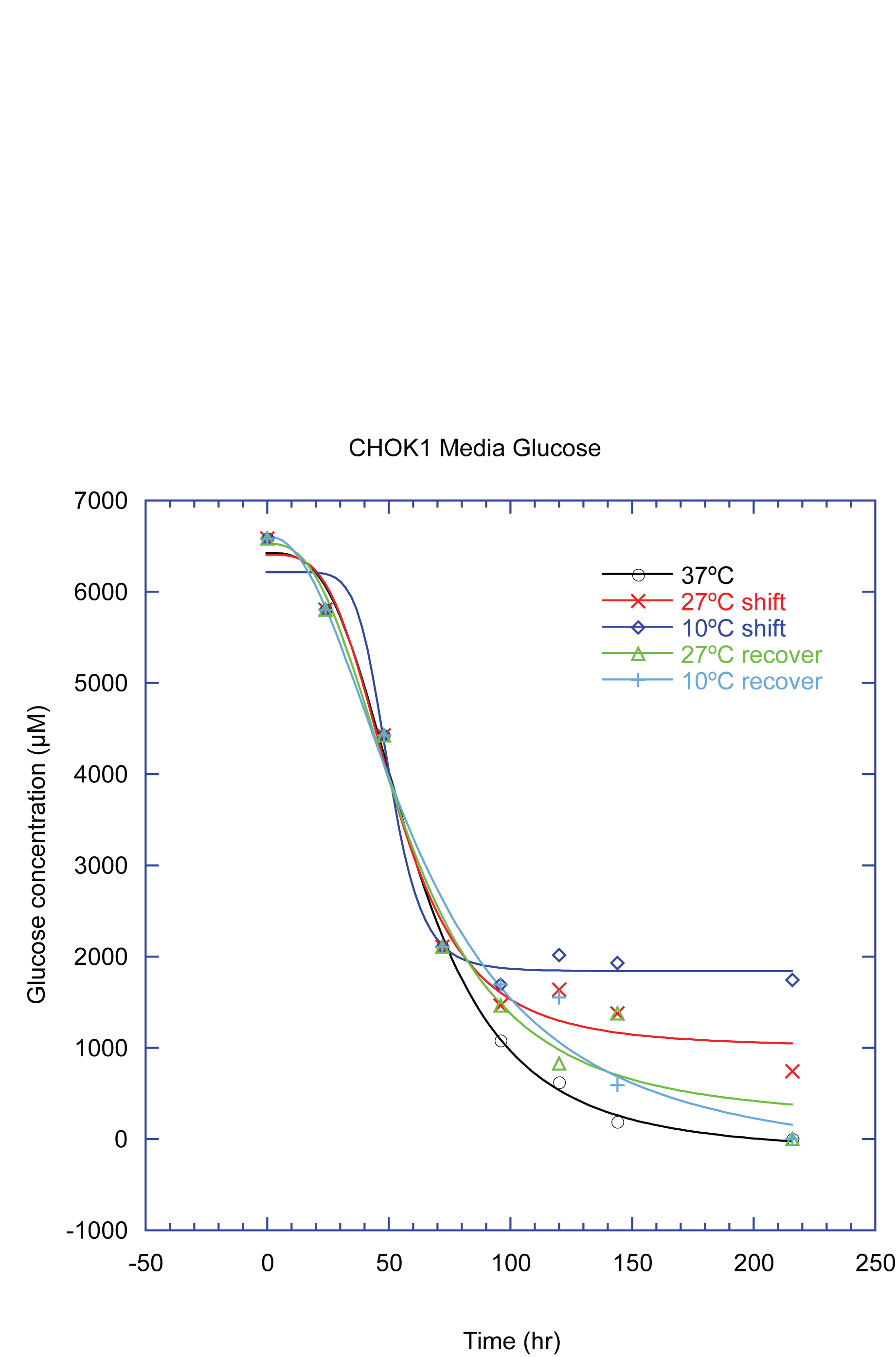


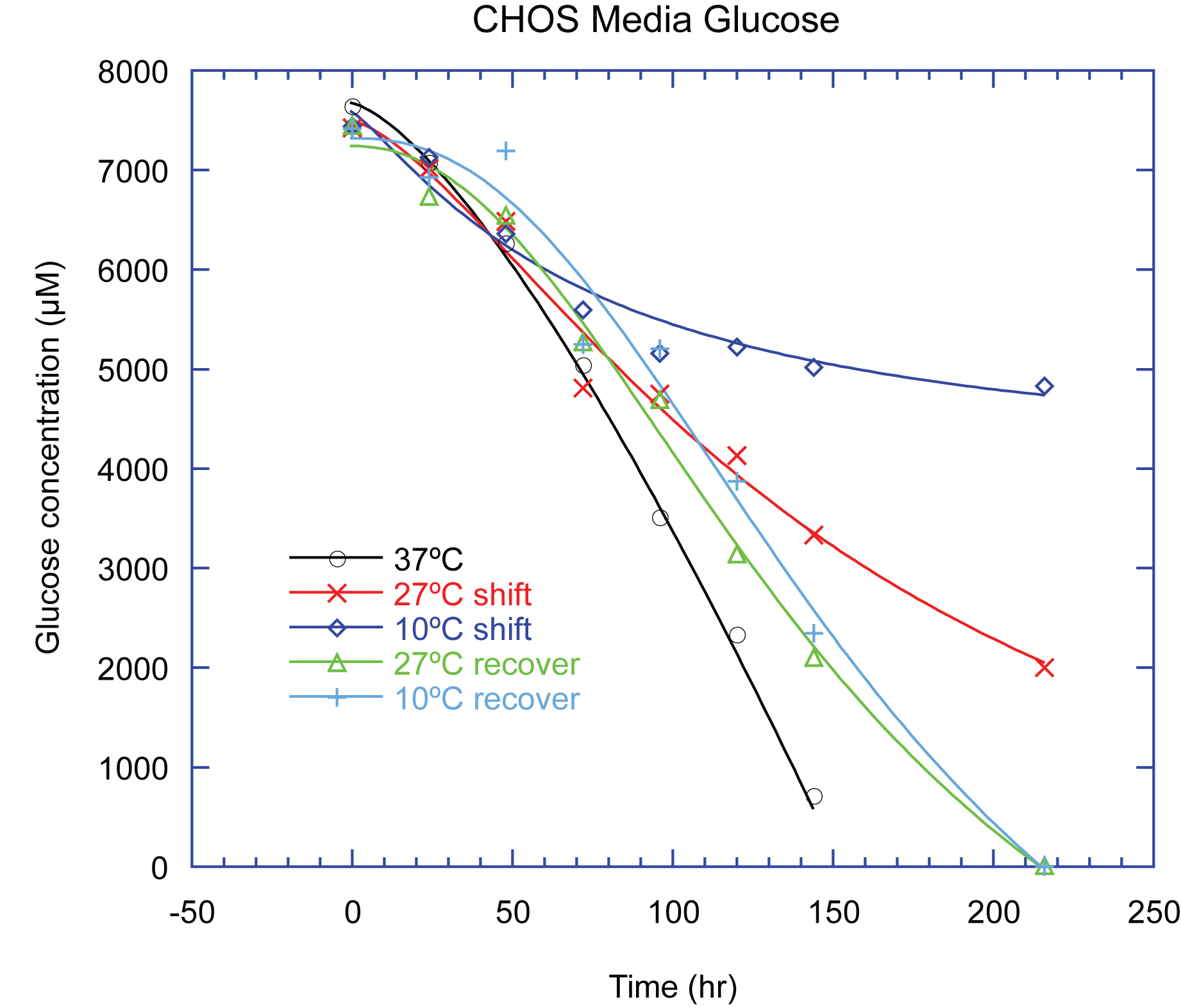


The utilisation of glucose by CHOS is not clearly defined as a sigmoidal process, unlike CHOK1. This is reflected in the large errors in the data S7 and highlighted in RED.

**Figure S2:** Sigmoid curves for lactate in CHOK1 and CHOS Media


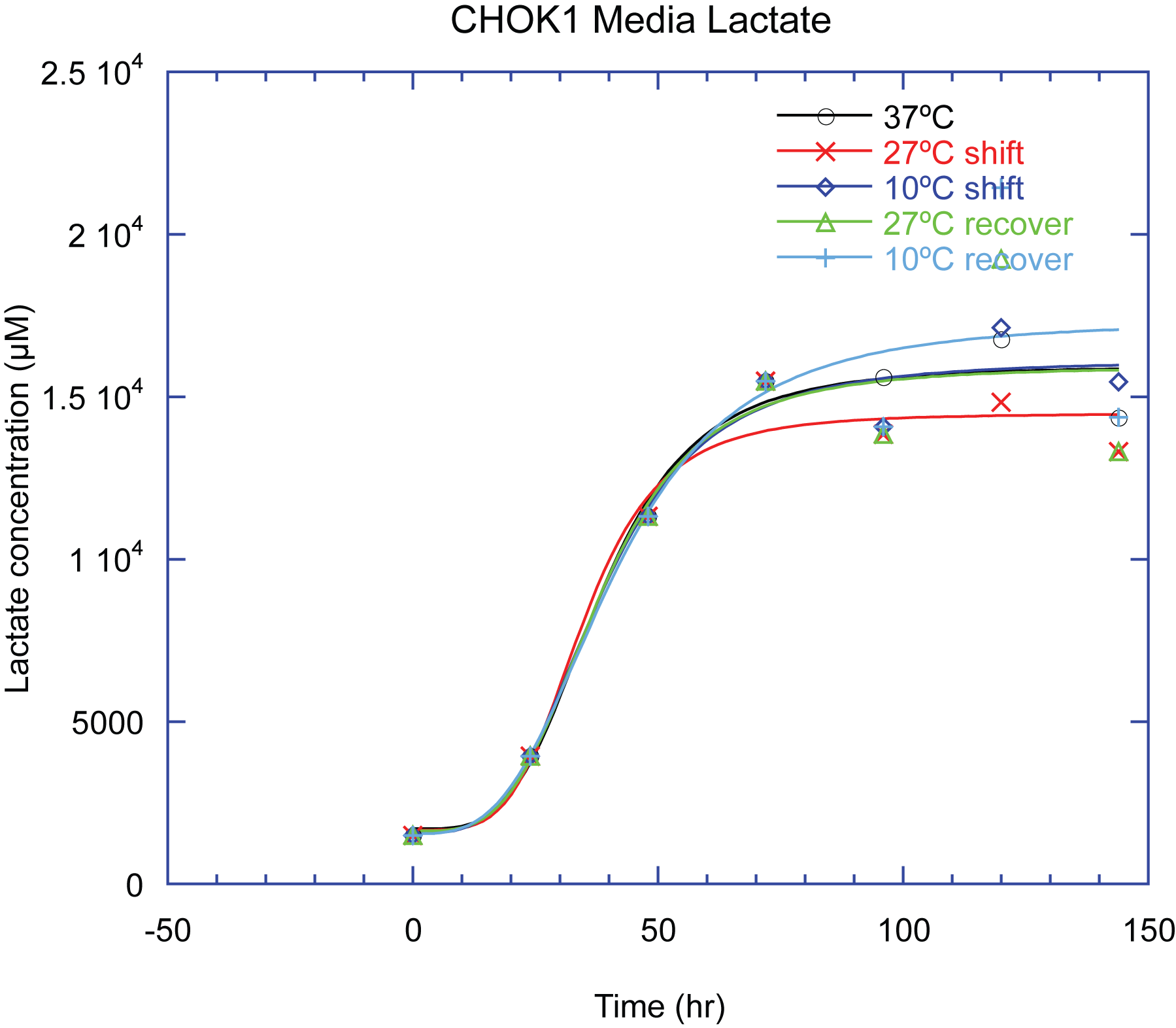


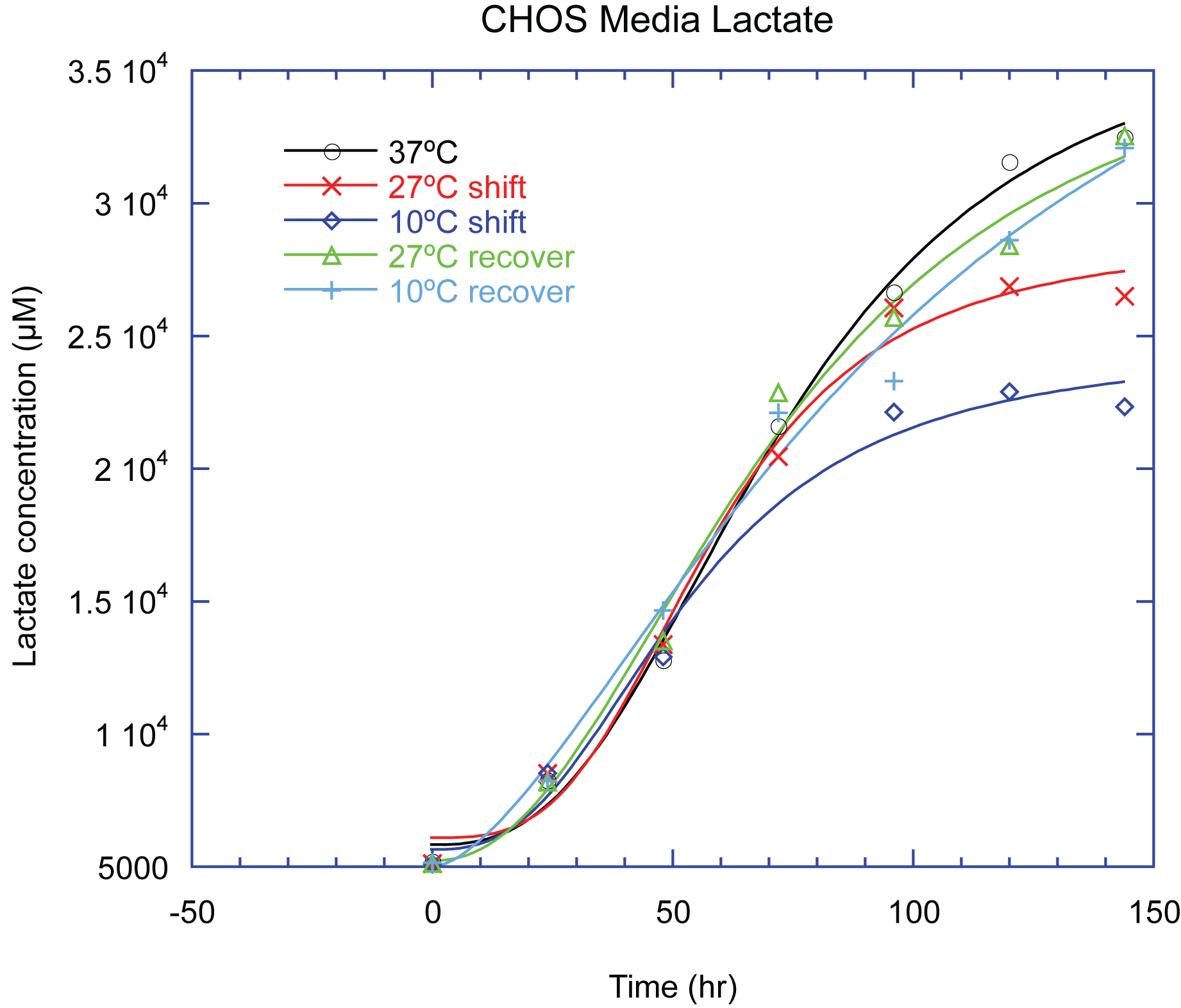


**Figure S3:** Sigmoid curves for alanine in CHOK1 and CHOS Media


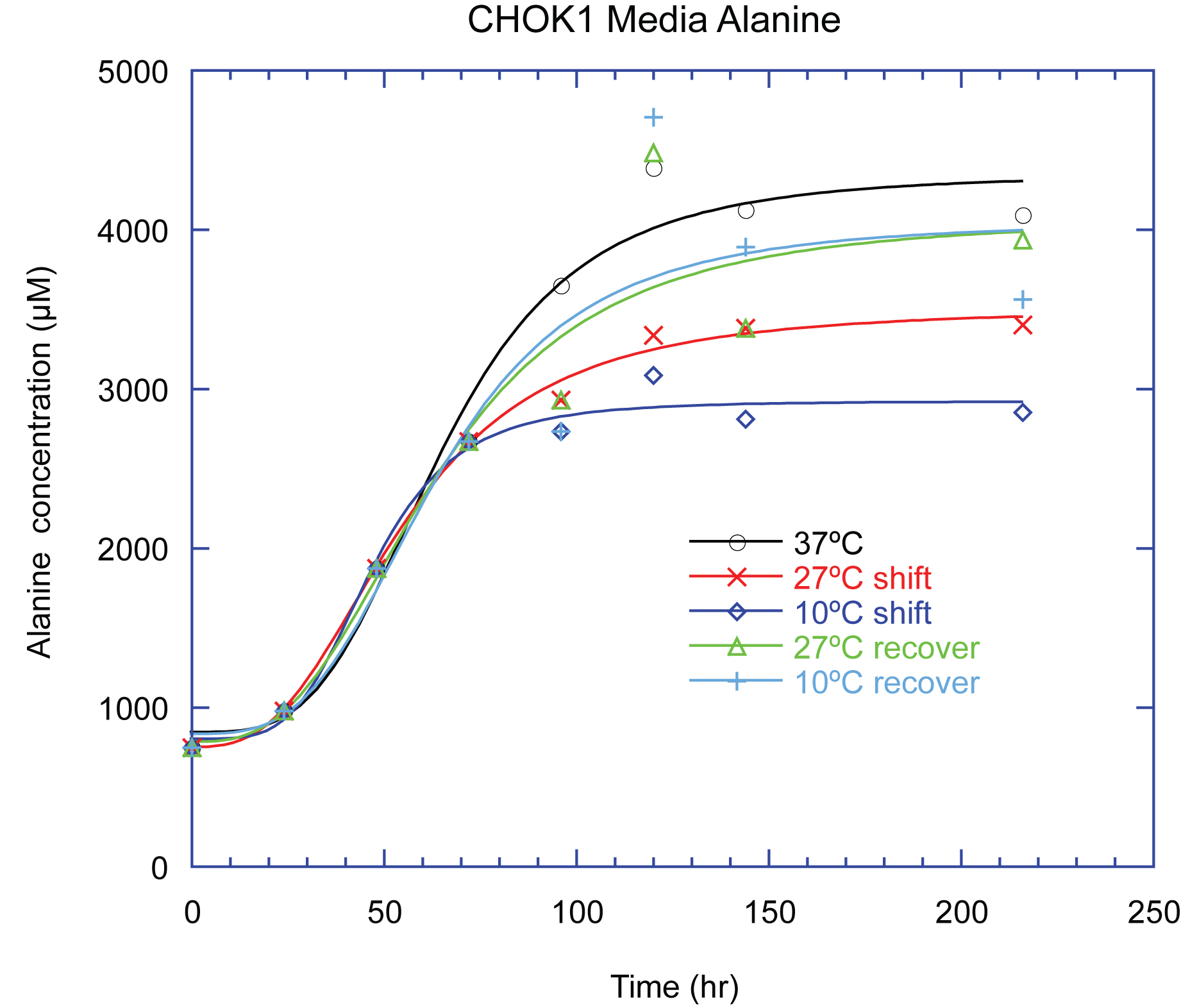


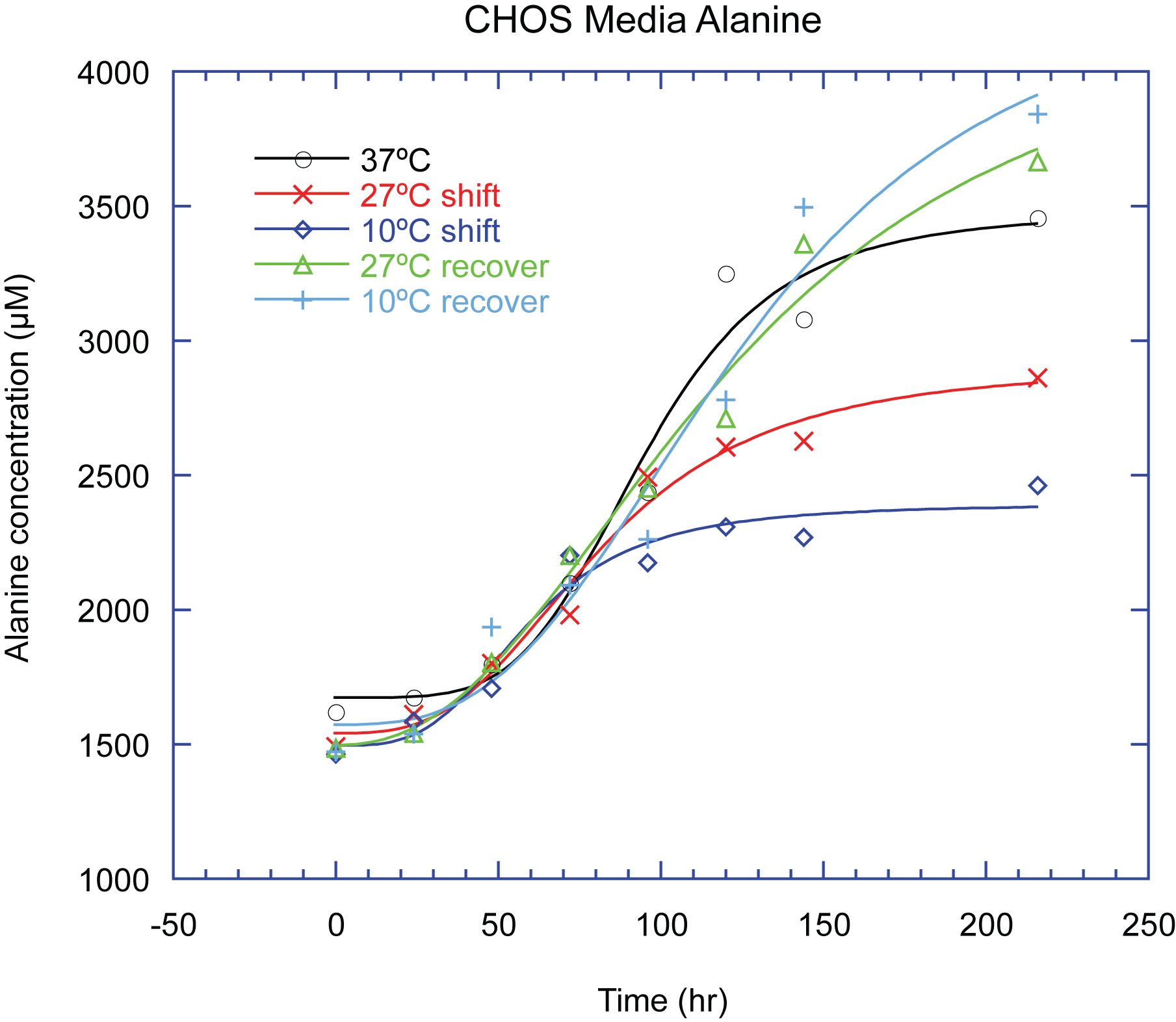


**Figure S4:** Sigmoid curves for isoleucine in CHOK1 and CHOS Media


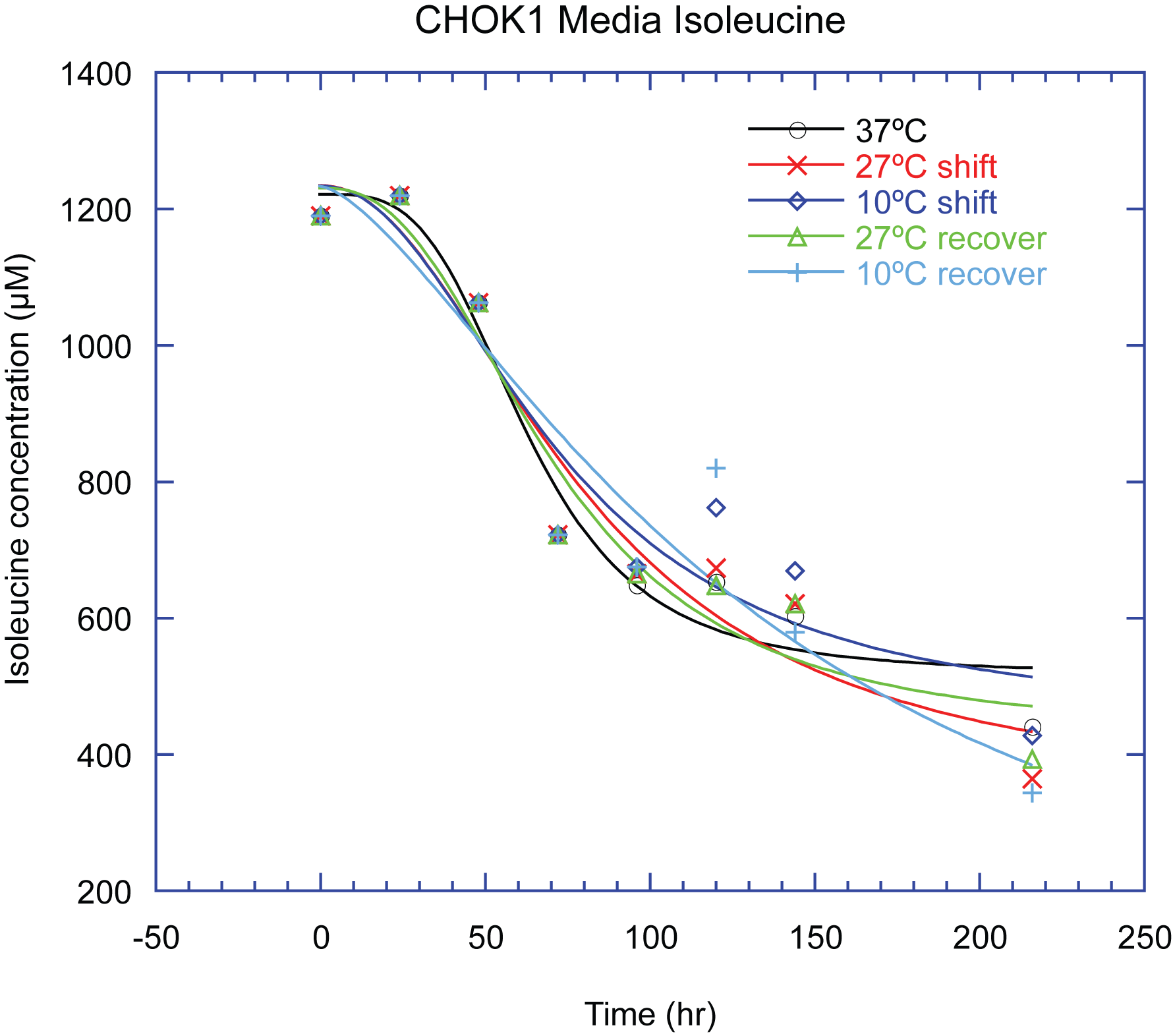


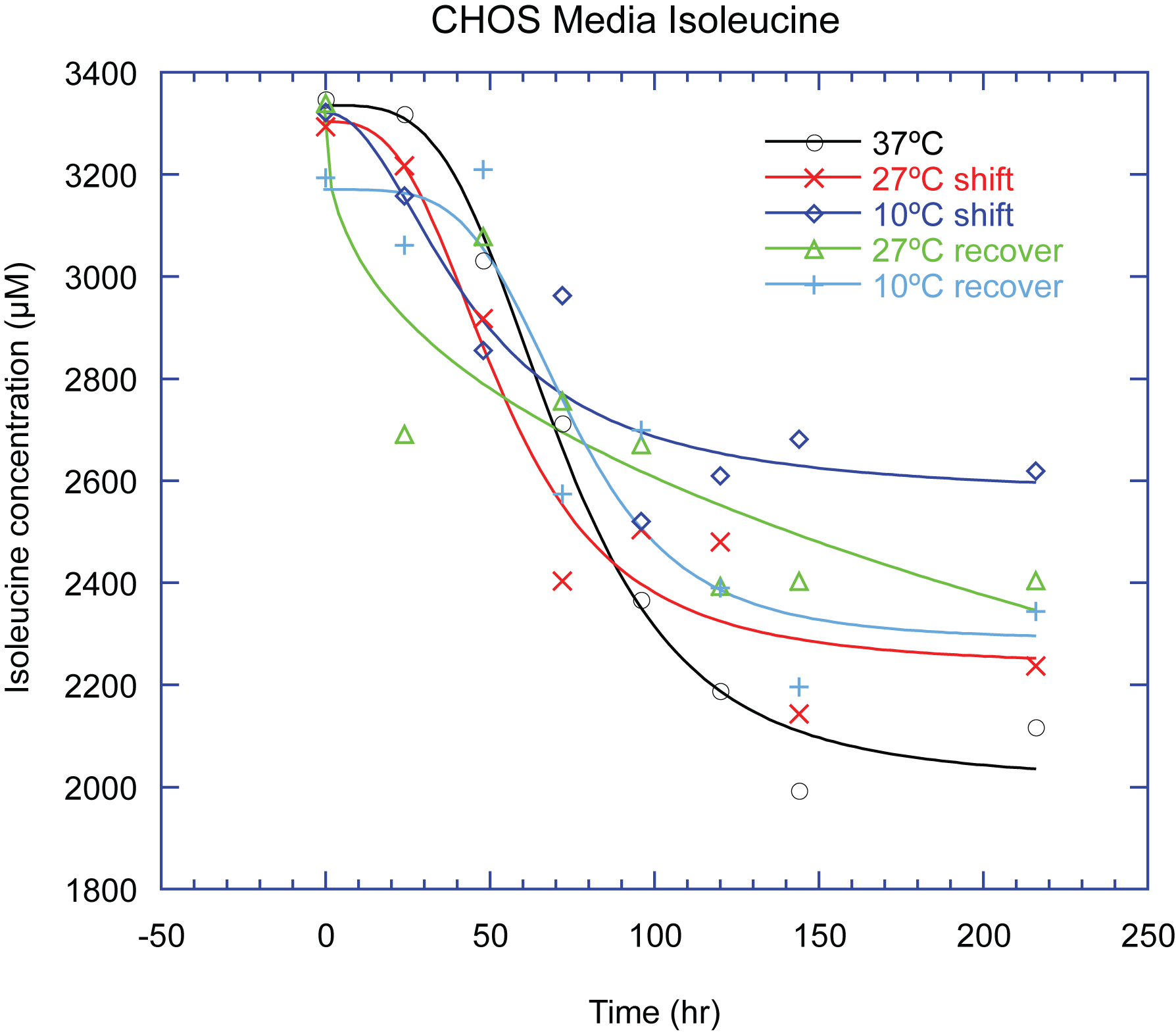


**Figure S5:** Sigmoid curves for glutamic acid in CHOK1 and CHOS Media


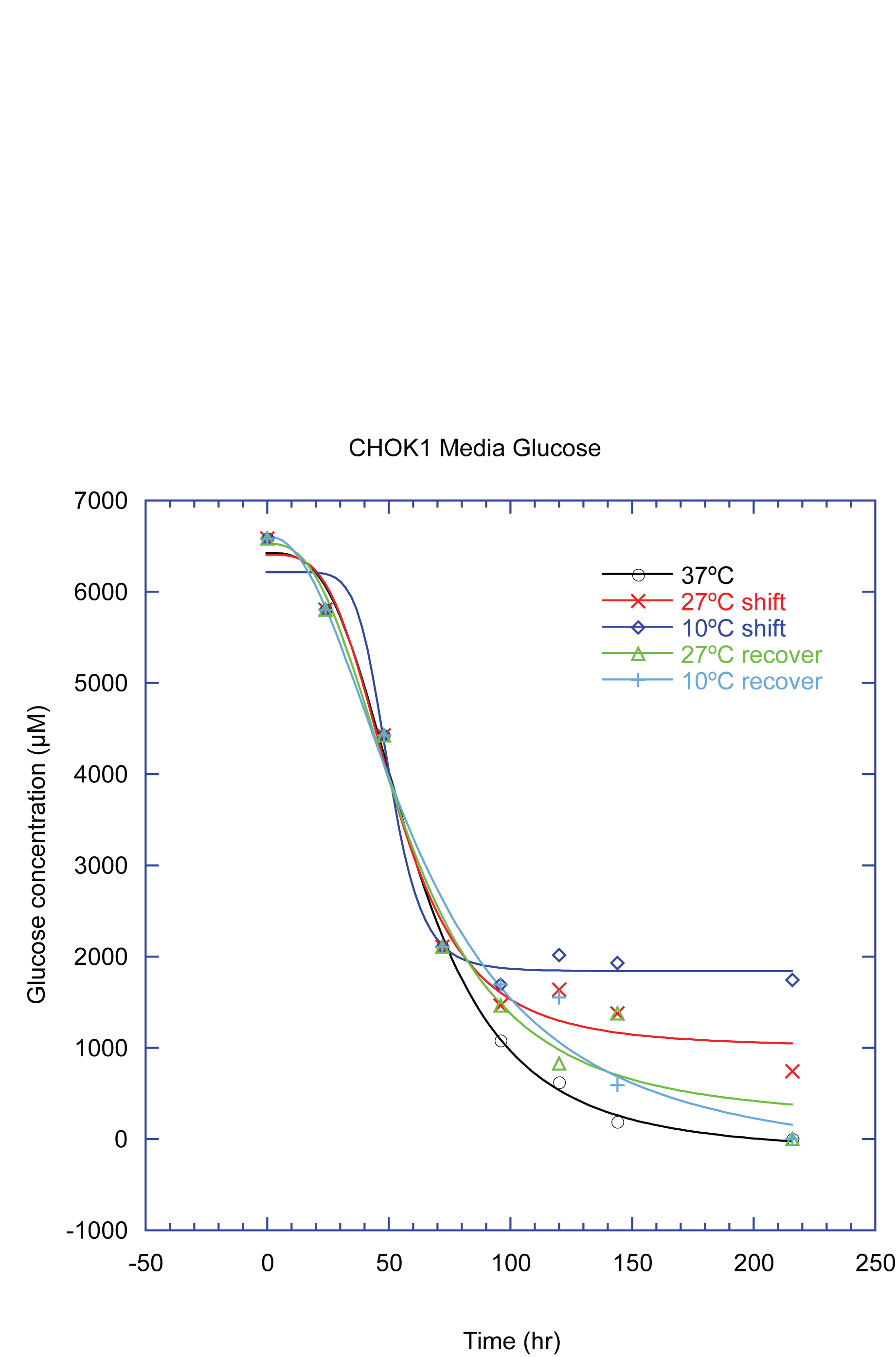


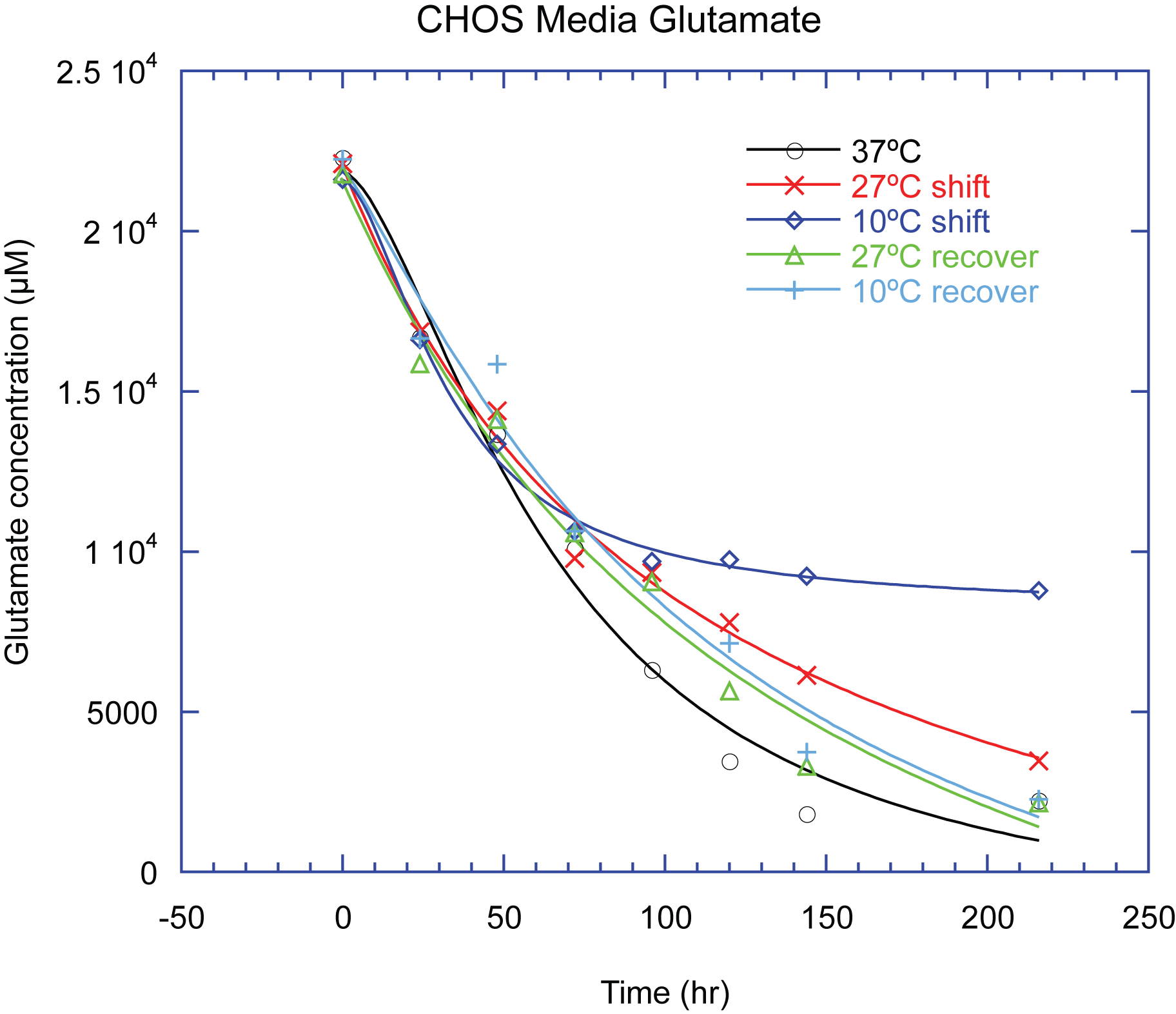


**Figure S6:** Sigmoid curves for pyruvate in CHOS Media


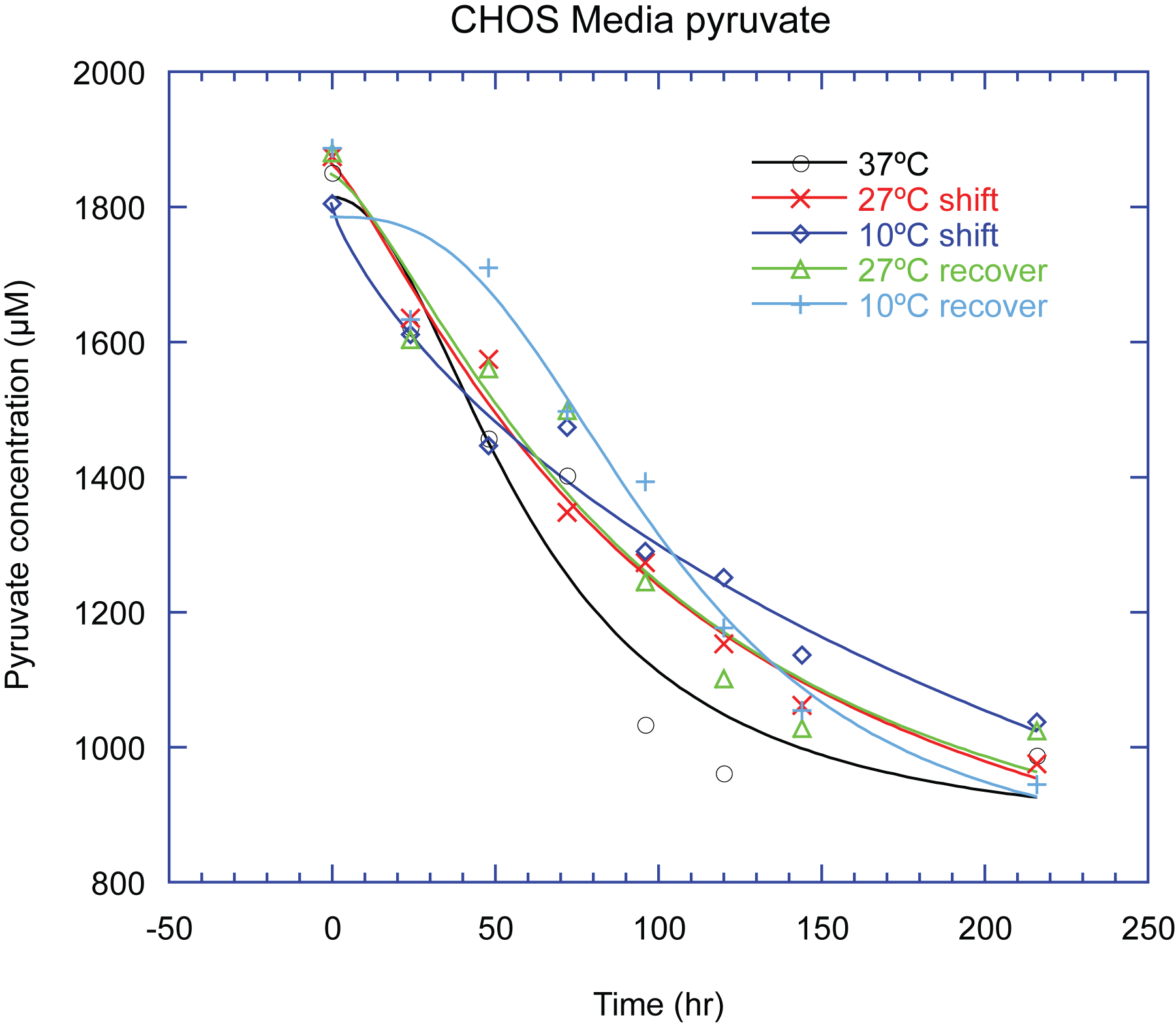


**Figure S7: PCA analysis of intracellular metabolites**

Shown below is the principal component analysis of extracellular metabolites of CHOK1 (A+B) and CHOS (C+D) cultures. The analysis of the identified extracellular metabolites by PCA is plotted by either culturing conditions (A+C) or timepoint (B+D).


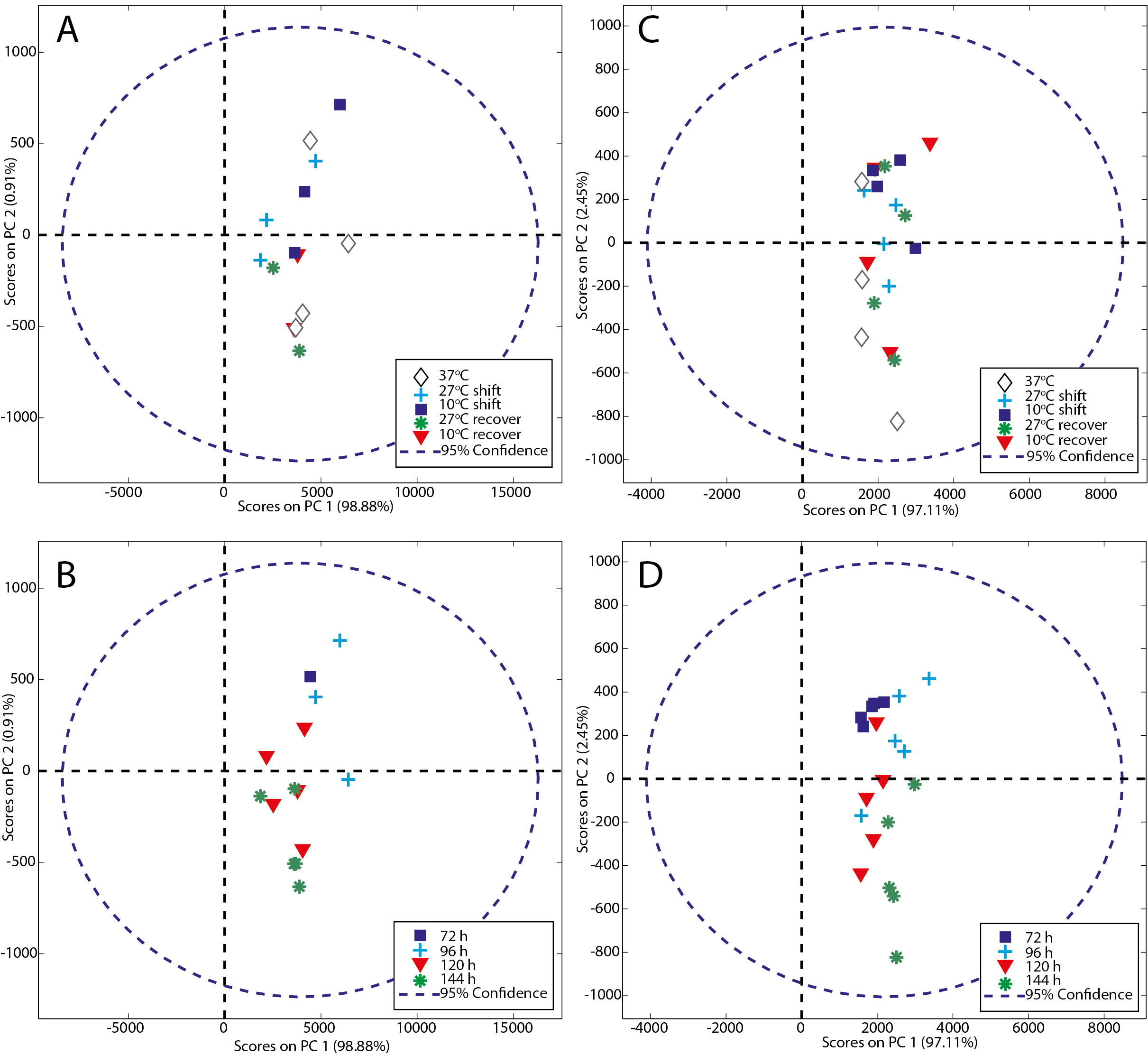


**Figure S8: Extracting protein from the CHOK1 media sample**

Low levels of protein present in the CHOK1 media affect the baseline of 1D proton spectra used for metabolite quantification as shown in the green spectrum below. This is especially noticeable in the amide region of spectrum (highlighted) the expanded region also shows the equivalent region of the protein extracted media (in blue) which has a flatter baseline enabling more accurate metabolite quantification.The additional peak in the extracted media is chloroform.


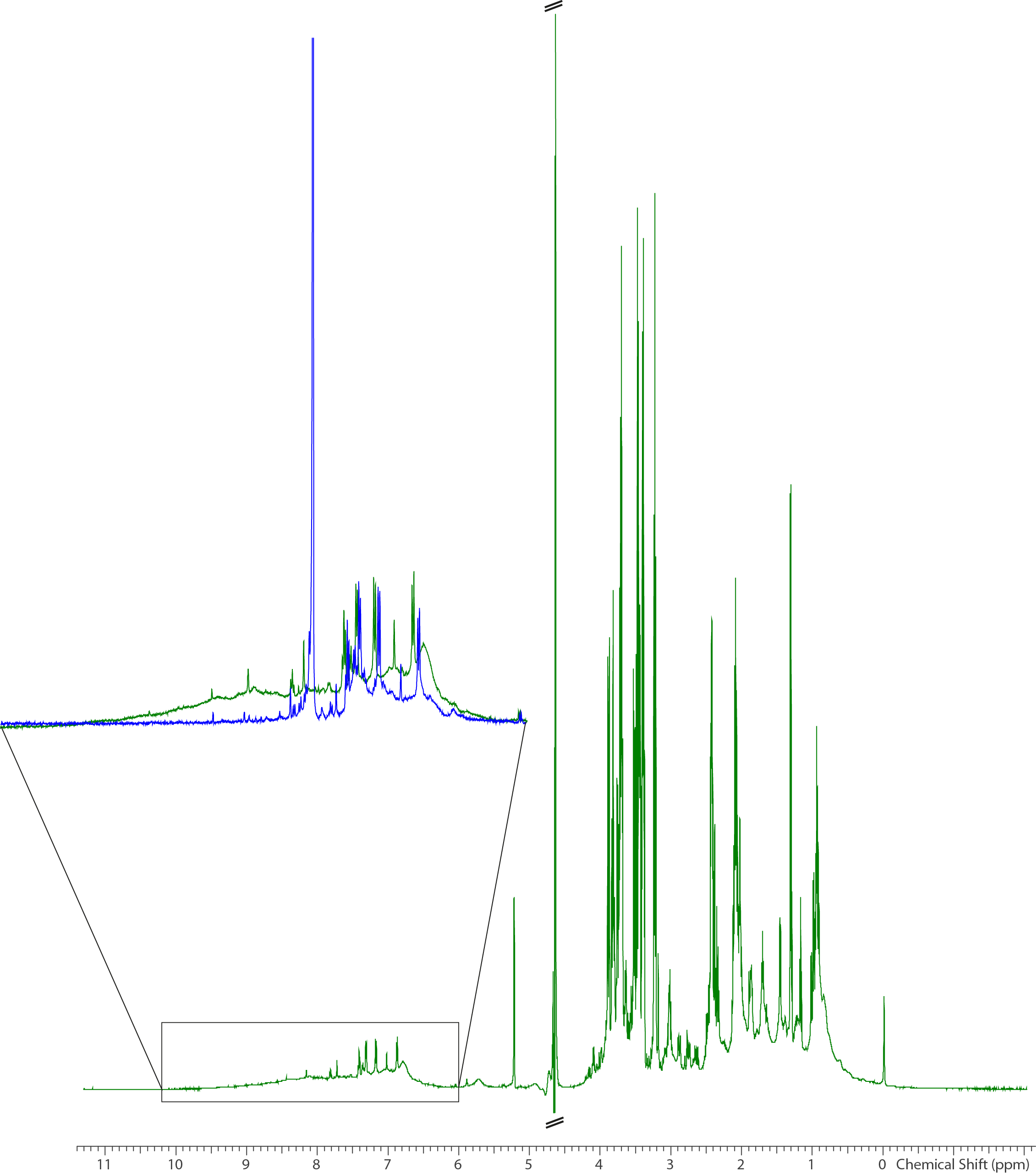


**Supplementary Figure S9: 1D ^1^H spectra of metabolites extracted from quenched cells**

Below are example spectra of extracted cell metabolites from A) CHOK1 and B) CHOS cells cultured at 37°C collected at 72 h after seeding. Spectra are shown at the same scale.

**
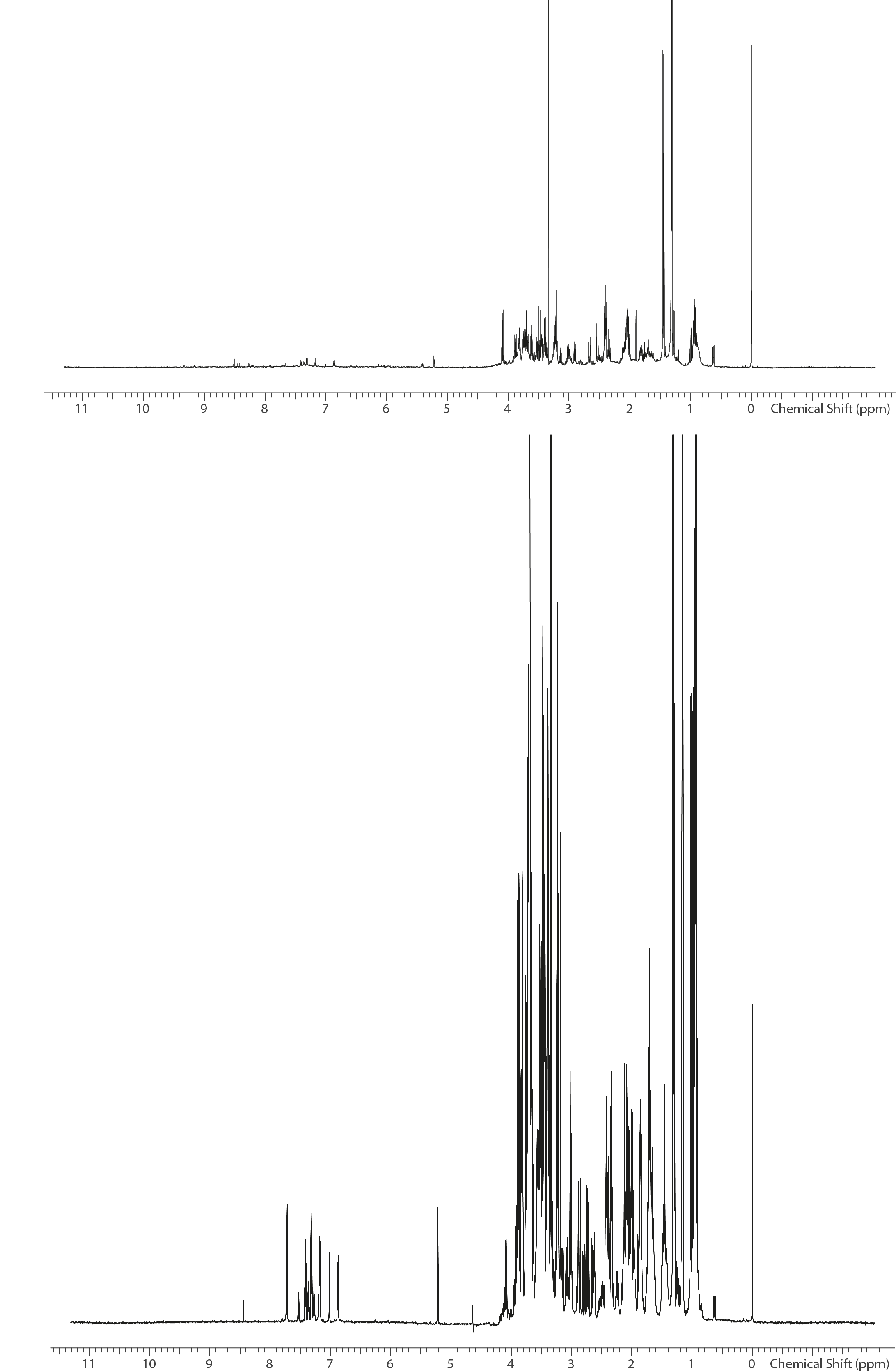
**

B)

A)
